# The early events underlying genome evolution in a localized *Sinorhizobium meliloti* population

**DOI:** 10.1186/s12864-016-2878-9

**Published:** 2016-08-05

**Authors:** Nicolás Toro, Francisco Martínez-Abarca, Manuel Fernández-López

**Affiliations:** Grupo de Ecología Genética, Estación Experimental del Zaidín, Consejo Superior de Investigaciones Científicas, Calle Profesor Albareda 1, 18008 Granada, Spain

**Keywords:** Diversity, Genome-wide sequencing, Genomic islands, Group II introns, Insertion sequences, Illumina technology, Polymorphism, Population genomics, Recombination

## Abstract

**Background:**

Population genetic analyses based on genome-wide sequencing data have been carried out for *Sinorhizobium medicae* and *S. meliloti,* two closely related bacterial species forming nitrogen-fixing symbioses with plants of the genus *Medicago*. However, genome coverage was low or the isolates had a broad geographic distribution, making it difficult to interpret the estimated diversity and to unravel the early events underlying population genetic variations and ecological differentiation.

**Results:**

Here, to gain insight into the early genome level variation and diversification within *S. meliloti* populations, we first used Illumina paired-end reads technology to sequence a new clone of *S. meliloti* strain GR4, a highly competitive strain for alfalfa nodulation. The Illumina data and the GR4 genome sequence previously obtained with 454 technology were used to generate a high-quality reference genome sequence. We then used Illumina technology to sequence the genomes of 13 *S. meliloti* isolates representative of the genomic variation within the GR4-type population, obtained from a single field site with a high degree of coverage. The genome sequences obtained were analyzed to determine nucleotide diversity, divergence times, polymorphism and genomic variation. Similar low levels of nucleotide diversity were observed for the chromosome, pSymB and pSymA replicons. The isolates displayed other types of variation, such as indels, recombination events, genomic island excision and the transposition of mobile elements.

**Conclusions:**

Our results suggest that the GR4-type population has experienced a process of demographic expansion and behaves as a stable genotypic cluster of genome-wide similarity, with most of the genome following a clonal pattern of evolution. Although some of genetic variation detected within the GR4-type population is probably due to genetic drift, others might be important in diversification and environmental adaptation.

**Electronic supplementary material:**

The online version of this article (doi:10.1186/s12864-016-2878-9) contains supplementary material, which is available to authorized users.

## Background

Progress in high-throughput sequencing technologies has facilitated the sequencing of complete genomes for many bacterial isolates, leading to advances in population genomic studies and providing insight into the forces driving adaptation and speciation in bacteria. These forces include natural selection, genetic drift and gene flow, but geographic isolation also acts as an ecological factor, affecting the outcome of the interplay between these evolutionary forces [1–4]. Nevertheless, genome-wide sequence studies generally focus on either isolates with a broad geographic distribution representing the diversity within the species, or on local populations with poorly defined structures, generally due to the sampling methods used [5]. The use of such approaches makes it difficult to interpret the estimated diversity [6] and to unravel the early events underlying the emergence and ecological differentiation of bacterial lineages.

Rhizobia are generally considered to be a group of gram-negative nitrogen-fixing bacteria eliciting the formation of root nodules on leguminous plants, within which they convert the atmospheric nitrogen (N_2_) unavailable to plants into ammonia. This fundamental process is essential for cellular life on Earth. Rhizobia include α-proteobacteria and β-proteobacteria [6]. Within the genus *Sinorhizobium* (syn. *Ensifer*), *S. meliloti* and *S. medicae* are closely related species forming symbioses with plants of the genus *Medicago*. Both *S. meliloti* and *S. medicae* have genomes consisting of a single circular chromosome (~3.65 Mb) plus two large symbiotic (sym) plasmids of ~1.3 (megaplasmids) and ~1.6 Mb (chromids) in size [7–12], and additional smaller accessory plasmids.

Population genetic analyses based on genome-wide sequence data have been carried out for *S. medicae* and *S. meliloti* [13–15]. The study on *S. medicae* [13] was performed on a localized population in symbiosis with *M. lupulina,* by comparison of the partial genome sequences of 12 randomly sampled isolates. However, genome coverage was low (0.8x) for each of the genomes sequenced. Two other studies [14, 15] characterizing genomic diversity with a high degree of genome coverage (100x) were recently carried out on 12 *S. medicae* and 32 *S. meliloti* strains, but with sampling from a germplasm collection representative of different multilocus genotypes, and from natural populations from different geographic locations [16, 17].

*S. meliloti* strain GR4 is a bacterium that is highly competitive for nodulation on alfalfa. It was first isolated over 35 years ago [18], from alfalfa nodules, at the Estación Experimental del Zaidín (Granada, Spain) field site. In addition to the chromosome and the symbiotic megaplasmids pRmeGR4c (pSymA) and pRmeGR4d (pSymB), it harbors two accessory plasmids designated pRmeGR4a and pRmeGR4b. We recently reported the complete 7,139,558 bp genome sequence of strain GR4 [19]. GR4-type isolates were subsequently obtained from alfalfa root nodules growing on plants at the same field site in the fall of 1996 and the summer of 1997 [20]. These isolates accounted for about 49 % of the isolates obtained. They were characterized by the presence of a *S. meliloti* GR4 strain-specific genetic marker (*dapB* gene) located on the accessory plasmid pRmeGR4b [20]. A sample of this population (319 isolates) was further fingerprinted with IS*Rm2011-2* and the group II intron RmInt1 as DNA probes, to confirm the overall genomic structure. Fingerprint analysis showed that 268 of the 319 isolates analyzed (84 %) clustered with the GR4-type group, whereas 51 isolates (16 %) corresponded to a distinct population (EM2-type) obtained from nodules also occupied by GR4-type bacteria. The fingerprints of 209 of the 268 GR4-type isolates (78 %) were identical to that of strain GR4, whereas the other 59 isolates (22 %) displayed genetic variation that could be classified into 34 patterns [20].

For the generation of high-quality GR4 reference genome data for this study, we used Illumina technology to sequence a new clone of strain GR4, to investigate genomic variation and to correct the GR4 genome sequence previously obtained with 454-technology [19]. We then used Illumina technology to sequence the genomes of 13 *S. meliloti* isolates representative of genomic variation within the GR4-type population, obtained from a single field site, with a mean coverage of more than 80x. We analyzed the genome variation within this population, to gain insight into the early forces driving genome evolution in *S. meliloti* populations and likely to give rise to diversification and ecological differentiation.

## Results

### Genome sequences of GR4-type isolates and a new clone of strain GR4

Isolates representative of the genomic variation within the GR4-type population were analyzed by IS and intron fingerprinting, and the dendrograms constructed by the UPGMA method indicated that they could be clustered into two main groups (Additional file 1: Figure S1). Representatives of the two groups, corresponding to 13 variants of the GR4 fingerprint pattern, were chosen at random for genome-wide sequence analysis with Illumina technology. We also sequenced the genome of a new clone of strain GR4, using Illumina technology to compare with the GR4 genome sequence previously obtained with 454 (Roche) technology and to investigate strain variation. The genomes of strain GR4 and the field isolates were sequenced to a mean depth of over 80x.

For the generation of high-quality GR4 reference genome data for this study, the Illumina reads obtained were mapped onto the GR4 reference genome sequence, as a guide. We identified 59 sequence differences due to 454 errors or assembler errors, which were corrected by the pairwise mapping of Illumina reads at 100 % identity, and revised on a Newbler assembly of 454 sequencing reads (Additional file 2: Table S1). The *S. meliloti* GR4 genome was thus stable under laboratory conditions.

### Mapping of Illumina data reads onto the strain GR4 reference genome

The Illumina reads of the isolate genomes were aligned (Additional file 3: Table S2) with the curated *S. meliloti* GR4 reference genome (chromosome, pSymB, pSymA, and accessory plasmids pRmeGR4a and pRmeGR4b). A mean of 99.84 % of the positions in the chromosome, 99.65 % of those in pSymB, 99.97 % in pSymA and 99.99 % in pRmeGR4b were covered, with a mean of 63, 54, 57 and 35 reads per site, respectively. Lower coverage was achieved for the pRmeGR4b plasmid of the G5 isolate (~60 % of the positions) because part of this plasmid, including the *dapB* gene, was missing. This suggests that G5 was obtained from a nodule occupied by more than one GR4-type bacterium. The pRmeGR4a plasmid was absent from most of the GR4-type isolates, but 100 % of the positions in the reference plasmid were covered by reads from the new clone of strain GR4, with a mean of 34 reads per site. By contrast, coverage was lower (81–83 % of positions) for the isolates harboring pRmeGR4a (G7 and G13), reflecting the absence of some regions. No copies of IS*Rm2011-2* or RmInt1 were found in pRmeGR4a, which did not, therefore, contribute to the IS and group II intron fingerprints described above.

The relative numbers of reads mapping to the GR4 chromosome, pSymB and pSymA were similar to expectations based on the relative sizes of these replicons (50, 23 and 19 %). However, far fewer reads than expected on the basis of size mapped to pRmeGR4b (half the number expected) and pRmeGR4a (a quarter the number expected), suggesting that these smaller replicons may have decreased in abundance or been completely lost from some cells during bacterial growth.

### Single-nucleotide polymorphism analysis

Single-nucleotide polymorphisms (SNPs) are among the most sensitive phylogenetic markers for the reconstruction of evolutionary history. We therefore carried out a SNP analysis on the alignment of the GR4-type isolate Illumina reads with the sequences of the three major replicons conserved in *S. meliloti* species: the chromosome, pSymA and pSymB. The accessory pRmeGR4b plasmid was not included in the SNP analysis due to the lower depth of sequence coverage, the small number of SNPs (mean of 3), and the absence of part of this plasmid from isolate G5.

**Fig. 1:**
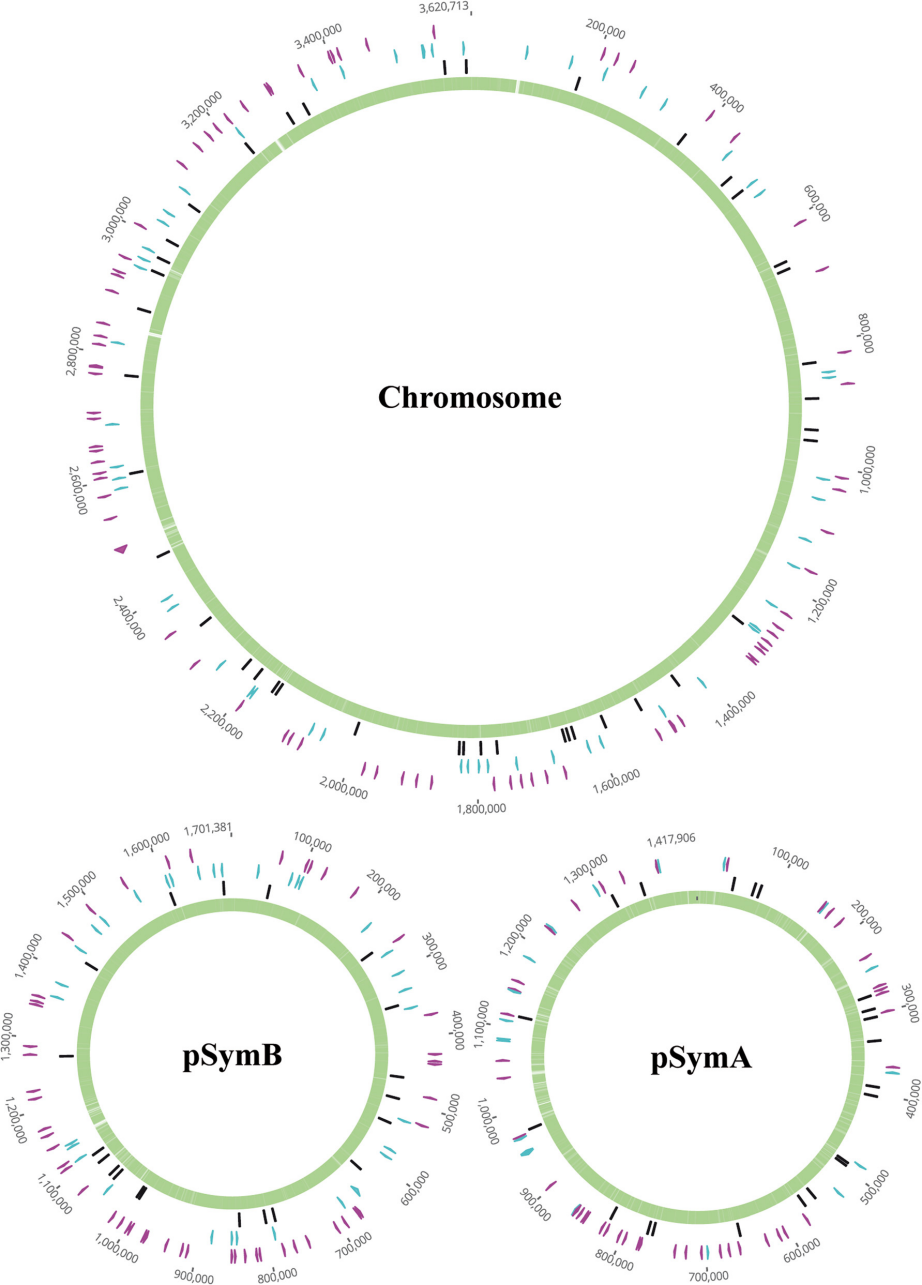
Distribution of SNPs on the replicons. The names of the replicons are indicated. sSNPs are indicated in blue, nsSNPs in pink and iSNPs are shown in black. Light green and white background, respectively; indicate island and non-island regions in the most likely global assignment of CpG islands to the sequence. To improve their visualization the SNPs are placed out of the sequence

We determined the types of nucleotides segregating (Table 1) in coding (synonymous/non-synonymous) and non-coding sequences. We identified 370 SNP sites, 109 (29.5 %) of which were synonymous (sSNPs), 178 (48.1 %) of which were non-synonymous (nsSNPs) and 83 (22.%) of which were present in intergenic regions (iSNPs) (Additional files 4, 5 and 6: Tables S3-S5). We found no differential selection for functional categories of proteins based on GO (Gene Ontology) annotations for the CDS carrying SNPs (Additional file 7: Figure S2). Moreover, mapping of the sequences flanking the nucleotide substitutions (50 nts) to the genome of *S. meliloti* strain 1021 [21] showed that 17 of the 83 iSNPs were located within potential non-coding RNAs and various types of transcriptional start sites (TSSs) (Additional file 6: Table S5). SNP numbers were distributed according to the relative size of the replicon, with a roughly uniform distribution around each replicon (Fig. 1). There were 1.63 times more nsSNPs than sSNPs. Assuming that the probability of mutation was the same for all positions (chromosome, pSymA and pSymB) and that there was no selection pressure, we obtained a nsSNPs/sSNPs ratio of 3.11:1 as the null hypothesis. The small number of SNPs and the ratio actually obtained therefore suggest that the population may have experienced selection and/or demographic processes decreasing diversity.

**Table 1:** Type of nucleotides segregating in the GR4-type isolates

	Total Sites	Chr/sites	pSymB/sites	pSymA/sites	Chr/SNPs^a^	pSymB/SNPs^a^	pSymA/SNPs^a^	Total SNPs
sSNPs	109	56	34	19	126	56	47	229
nSNPs	178	80	53	45	201	129	115	445
iSNPs	83	41	20	22	78	84	78	240
All SNPs	370	177	107	86	405	269	240	914
					Chr	pSymB	pSymA	Total
					Size of Replicon 3,620,713	1,701,381	1,417,906	6,740,001

^a^Total SNPs per replicon in the isolates

**Fig. 2:**
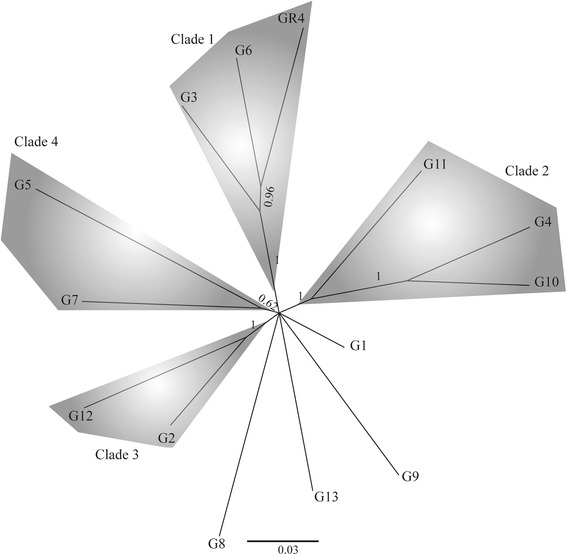
Phylogeny of GR4-type isolates based on alignments of SNP types. Phylogeny based on all SNPs (370 nt positions). Phylogenetic trees were inferred from Bayesian analyses and posterior probabilities (≥50 %) are indicated at the nodes. The names of the isolates and clades are indicated

All sSNPs, nsSNPs and iSNPs were concatenated into a continuous sequence (370 nts), and a phylogenetic tree (Fig. 2) was inferred by Bayesian analysis [22] from the corresponding 14-sequence alignment (Additional file 8: Figure S3). Most of the GR4-type isolates could be clustered into four clades: clade 1, which includes strain GR4, and the G3 and G6 isolates; clade 2, which includes G4, G10 and G11; clade 3, which includes the G2 and G12 isolates; and clade 4, which includes the G5 and G7 isolates. Similar tree topologies were obtained with different phylogenetic reconstruction methods, and for separate analyses of sSNPs, nsSNPs or iSNPs (not shown), but these analyses had a lower resolution, probably due to the smaller number of informative positions.

### Diversity of the isolates and divergence

Tajimas’s *D* was calculated for all sequence pairs for concatenated genes carrying SNPs (sSNPs, Additional file 9: Table S6; nsSNPs, Additional file 10: Table S7) for each replicon (121 genes for the chromosome, over 146,721 nt; 63 genes for pSymB, over 76,977 nt; and 58 genes for pSymA, over 66,945 nts) and revealed similar low levels of nucleotide diversity for the chromosome, pSymB and pSymA. There were 0.000135, 0.000142 and 0.000136 changes per site, and strongly negative *D* values (statistical significance *P* < 0.01) of -2.18, -2.12, and -2.20, respectively, were obtained (Table 2). We observed no significant skewing of nucleotide diversity between the two halves of the chromosome. Moreover, Fu and Li’s D*, Fu and Li’s F*, and Fu’s *Fs* test of neutrality also yielded significant negative values, as did the estimated Tajima’s *D* values for synonymous and non-synonymous sites (Table 2). The highly negative values of these statistics provide possible evidence of a population expansion.

**Table 2:** Neutrality tests

Concatenated coding sequences carrying sSNPs and nsSNPs										
	nt positions	S^a^	π^b^	θ^c^	D^d^	D(Syn)^d^	D(nSyn)^d^	D^*e^	F^*f^	Fs^g^
Chromosome	146,721	123	0.000135	0.000264	−2.18**	−2.23**	−2.09**	−2.79**	−3.01**	−3.78**
1st half	64,742	56	0.000146	0.000272	−2.03*	−2.10**	−1.76	−2.54**	−2.76**	−6.85**
2nd half	81,972	67	0.000127	0.000257	−2.24**	−2.25**	−2.09**	−2.90**	−3.12**	−6.39**
pSymB	76,977	67	0.000142	0.000274	−2.12**	−1.83*	−2.19**	−2.60**	−2.84**	−6.13**
pSymA	66,945	58	0.000136	0.000272	−2.20**	−2.12**	−2.15**	−2.74**	−2.98**	−7.04**

^a^ Number of segregating sites

^b^ The mean number of pairwise nucleotide differences per site

^c^ The number of segregating mutations per site

^d^ Tajima’s *D* statistic, Syn (synonymous sites), nSyn (non-synonymous sites)

^e^ Fu and Li’s D* statistic

^f^ Fu and Li’s F* statistic

^g^ Fu’s *Fs* statistic

* Statically significant result (*P*-value <0.05)

** Statically significant result (*P*-value <0.01 or <0.02)

**Fig. 3:**
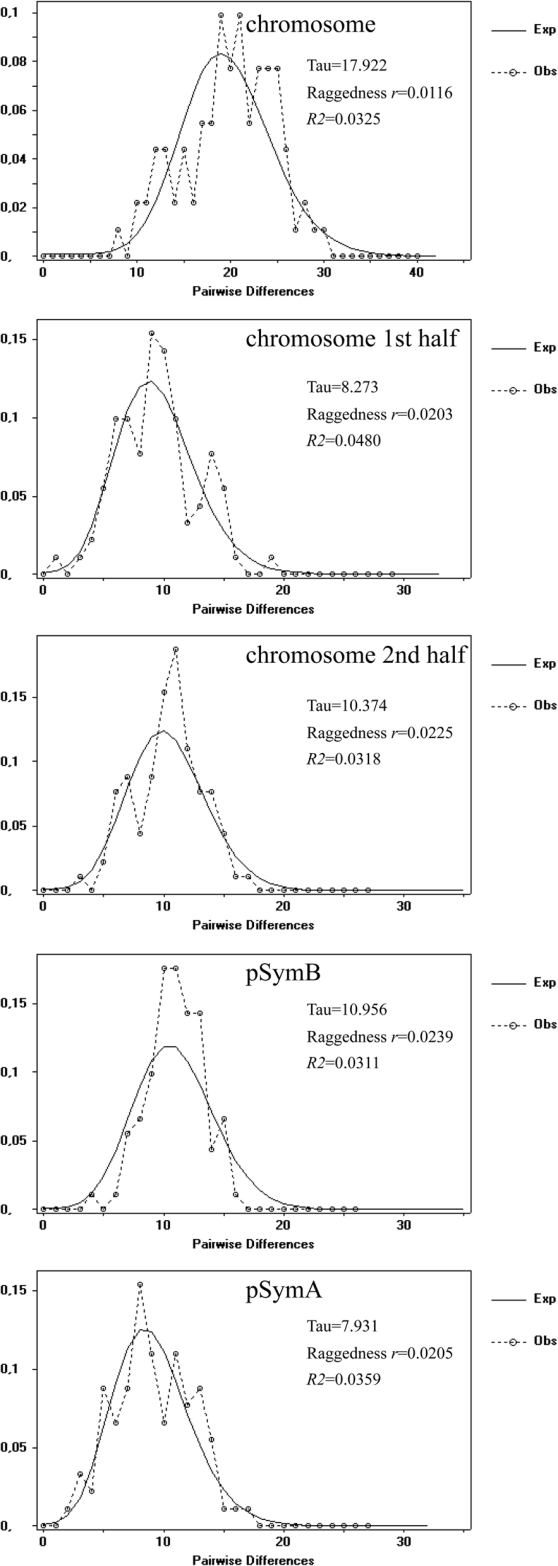
Mismatch distribution analysis for concatenated genes carrying SNPs. Graphs of the mismatch distribution are shown for each replicon and for the two halves of the chromosome. The *x*-axis shows the observed distribution of pairwise nucleotide differences, with frequency plotted on the *y*-axis. The values of the moment estimator Tau (τ), and the Raggedness index *r* and *R*_*2*_ statistics are shown

The unimodal curves of the mismatch distribution analyses for the three replicons and the low values of the Raggedness index *r* and the *R*_*2*_ statistic are consistent with the results of the neutrality test, and further suggest that the population has undergone expansion (Fig. 3). Based on the moment estimator (τ) and assuming an evolutionary rate of 2.03x10^-8^ mutations per site per year (Methods), this expansion was estimated to have begun around 3.0, 3.5 or 2.9 thousand years ago (Kya), depending on the dataset (chromosome, pSymB and pSymA, respectively).

**Fig. 4:**
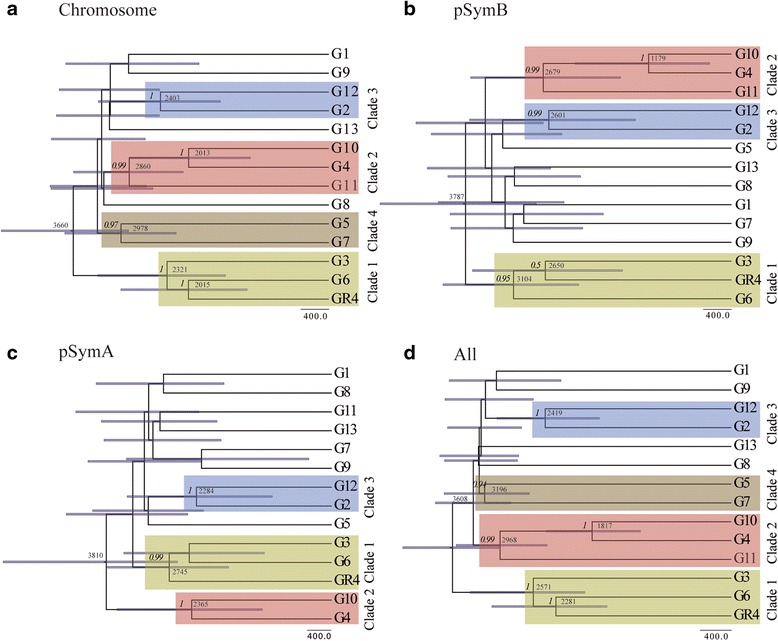
The maximum clade credibility tree estimated with BEAST 2, based on concatenated genes carrying SNPs. **a** Chromosome. **b** pSymB. **c** pSymA. **d** All concatenated genes carrying SNPs. Node bars indicate the 95 % credible intervals (95 % HPD) for node ages, and posterior probabilities (>0.5) are indicated (in italics) on the branches. Relevant node ages (years) and clades are indicated. The root of the unrooted tree was placed at the mid-point of the longest distance between two taxa in the tree

We investigated the demographic history of the GR4-type population further, by carrying out a Bayesian analysis with BEAST 2 software [23] to infer the genealogy of the isolates from the concatenated genes and to evaluate changes in population size over time. The topology of the species trees (Fig. 4) resembled that obtained by phylogenetic analysis with the concatenated SNPs, with four clearly distinguishable clades (Fig. 4a and d). The species trees inferred from pSymB or pSymA sequence data (Fig. 4b and c) had a lower resolution, presumably due to the corresponding concatenated sequences being shorter and, therefore, containing a smaller number of informative positions. Assuming a strict molecular clock and the mutation rate indicated above, we estimated a divergence time for these isolates of ~3.6 Kya (chromosome [Fig. 4a], 2.8–4.6 Kya, 95 % highest posterior density [HPD]; all genes carrying SNPs [Fig. 4d], 3.0–4.2 Kya, 95 % HPD). The GR4 strain lineage may have arisen about 2.4 Kya (chromosome [Fig. 4a], 1.1–2.9 Kya, 95 % HPD; all genes carrying SNPs [Fig. 4d], 1.6–3.1 Kya, 95 % HPD).

**Fig. 5:**
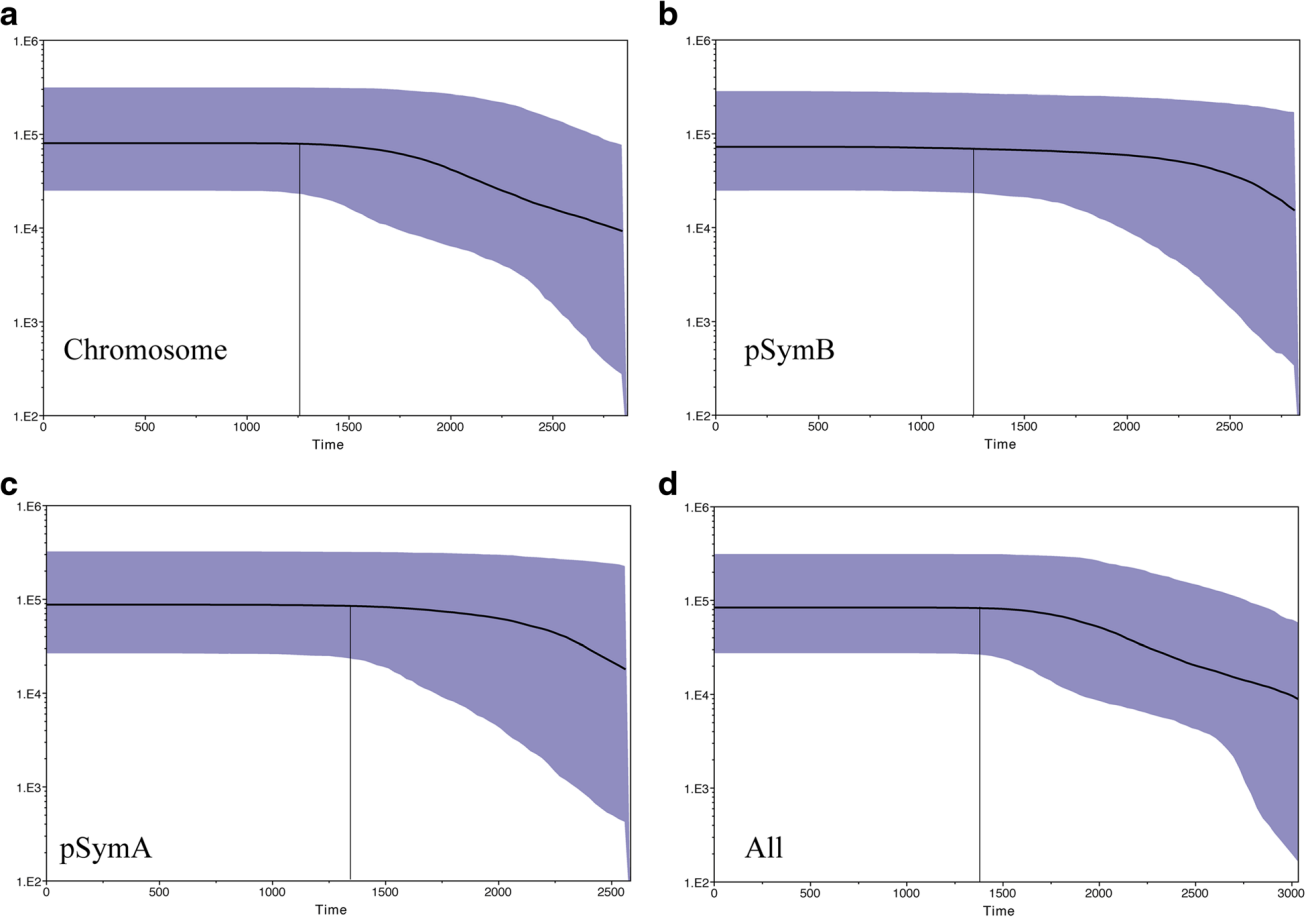
Bayesian skyline plots based on the concatenated genes carrying SNPs for each replicon. **a** Chromosome. **b** pSymB. **c** pSymA. **d** All concatenated genes carrying SNPs. The median estimates are shown as solid lines, and the blue areas indicate the limits of the 95 % HPD. The vertical line indicates the beginning of the stabilization period. Time (*x*-axis) is expressed in years ago. The effective population size is indicated on the *y*-axis

The Bayesian Skyline Plot (BSPs) based on the concatenated sequences for each replicon (Fig. 5) suggests that the GR4-type population increased in size from around 2.5–3.0 Kya. This timing is approximately consistent with that estimated with the moment estimator (τ). This population then began to stabilize about 1.25–1.37 Kya. These changes might be consistent with the early use of alfalfa as a forage crop in Spain [24]. Nevertheless, caution is required when interpreting the time divergence data, because very different estimates are obtained with other possible universal mutation rates [25].

### Detection of genomic variation in GR4-type isolates

We investigated the evolutionary history of this *S. meliloti* population further by analyzing other types of variation in the genomes of the isolates, such as indels (insertion/deletions), recombination, genomic island excision and/or integration, and variation associated with mobile elements.

#### Variation due to indels

The GR4 strain used for genome sequencing is actually a non-mucoid derivative of the original isolate, probably resulting from a frameshift mutation in the *expR* gene (GR4Chr3372) caused by the deletion of 11 nt (CGTCCGGCCAG) from the chromosome between nucleotide positions 3,530,664 and 3,530,665. All the isolates sequenced here had a mucoid phenotype, and, as expected, they carried the wild-type *expR*. An analysis of the isolates revealed that the GR4 strain carried another deletion, in a methyl-accepting internal chemotaxis (*icpA*/*mcpE*) gene (GR4Chr0624); 15 nt (AGCACCAGCGCCAGC) of this gene were deleted, between positions 671,852 and 671,853, resulting in the production of a chemoreceptor protein lacking five amino-acid residues (RQQHQ) after the N-terminal protoglobin region. This locus is the first ORF of the *S. meliloti* chemotaxis operon (*che* operon). The deletions described above are not present in field isolates and therefore appear to have occurred during the culture of the GR4 strain in laboratory conditions. However, other specific deletions (4 to 63 bp, Additional file 11: Table S8) and smaller indels (insertions, deletions) and substitutions (Additional files 12, 13 and 14: Tables S9-S11) were detected in some of the isolates. These types of variation affected coding sequences and intergenic regions, and some generated frameshifts or were located in the 5′and 3′ UTRs.

#### A genomic mid-range signal in the GR4-type population

**Fig. 6:**
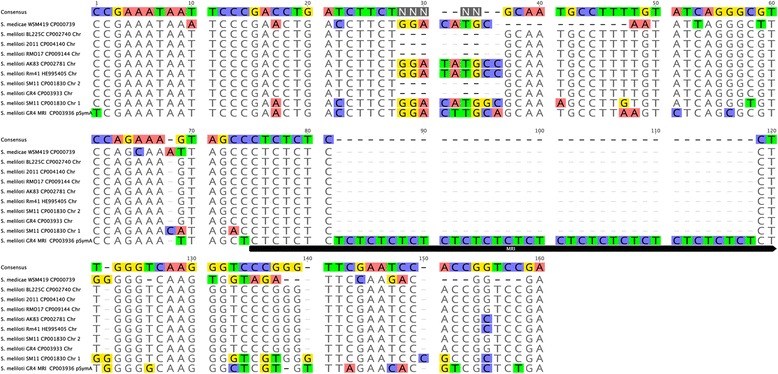
Pyrimidine/purine dinucleotide CT/GA-rich MRI (mid-range inhomogeneity) region. The identified MRI and flanking sequences in the pSymA of GR4-type isolates and that conserved on the chromosomes of *S. meliloti* and *S. medicae* are shown. Residues in color are different from the consensus sequence. The location of the replicon and GenBank accession numbers are shown

The G4, G5, G9 and G13 isolates were found to contain pSymA indels (16, 10, 15 and 8 bp deletions, respectively) in a pyrimidine/purine dinucleotide CT/GA-rich MRI (mid-range inhomogeneity) region [25, 26] of 46 bp in length in strain GR4 (162,000 to 162,045). This MRI is located in an IGR flanked by two ORFs, one encoding a hypothetical protein (GR4pC0152) and the other a DsrE/DsrF-like protein (GR4pC0153). This mid-range genomic signal of unknown function is absent from the sequenced genomes of other *S. meliloti* strains, and therefore appears to be unique to strain GR4. Nevertheless, the flanking regions and a shorter MRI (6 bp) appear to be conserved on the chromosome (positions 1,308,198 to 1,308,307 in GR4) in *S. meliloti* and *S. medicae* (Fig. 6). The acquisition of this region by pSymA in the GR4-type isolates may have been mediated by exchange with the chromosomal region and further replication slippage [27]. These regions are considered to be sites of genetic instability [27] potentially relating to physiology and adaptation to particular environmental niches [28].

#### Variation due to recombination

An analysis of the isolates revealed that the pSymB Ti-type conjugative transfer relaxase *traA2* (GR4pD0907) harbored by isolates G4 and G10 contained high levels of SNP accumulation. GR4 pSymB-*trA2* is 97.1 % identical to the pSymA-*trA1* (GR4pC0962) locus, so homologous recombination is likely to occur between these two long (4.6 kb) loci. Sequence alignments and phylogenetic analyses indicated that the pSymB-*trA2* of G4 and G10 was more closely related to pSymA-*trA1* (Additional file 15: Figure S4), consistent with a recombination event. The G4 and G10 isolates also displayed other common signatures of recombination (high levels of SNP accumulation) at IGR positions 1,116,938 to 1,117,089 of pSymA. This sequence is actually a repeat region in strain GR4, with a second copy (90.7 % identity) at positions 1,145,069 to 1,145,218 of pSymA; this second copy is conserved in other *S. meliloti* strains. In the G4 and G10 isolates, the sequences of both repeats were identical to that of the second repeat in GR4, indicating that recombination and replacement occurred in both isolates (Additional file 16: Figure S5).

The pSymB of G6 contained two long regions (Additional file 17: Figure S6) with features of genetic exchange and replacement that were not predicted to be genomic islands. The first region, spanning 48 kb (800,349 to 848,330), contained a large number of SNPs, but displayed synteny conservation for a block of orthologous genes. The second region encompassed almost 200 kb, but contained a large rearrangement, with many missing CDSs and a large accumulation of SNPs in the genes that appeared to be conserved, from coordinate positions 1,098,191 to 1,296,315. These results suggest that these genomic regions have been replaced with sequences from other sources.

#### Variation due to genomic islands

Genomic islands (GIs) are generally defined as any cluster of genes, typically 10–200 kb in length [29], acquired by horizontal gene transfer (HGT). Two active excised GIs were detected in the isolates (Additional file 18: Figure S7). Part of the larger (80,271 bp) predicted chromosomal GI (IslandViewer methods, [30]) was missing from isolate G5, and its left flanking site was found to be located close to a phage integrase (GR4Chr2369) spanning about 65 kb (positions 2,472,461 to 2,537,427). This region was conserved in all the other 12 isolates and in strain GR4. This GI (hereafter referred to as GI1) is flanked by direct repeats derived from a conserved *tRNA-*^*Met*^ gene (GR4Chr2368 and GR4Chr2412), and represents an ancient insertion resulting from a HGT event in the ancestor of the GR4-type population because: *i*) it was not found in the genomes of any of the other *S. meliloti* strains sequenced, and *ii*) it contains genes, such as the large RHS re-associated core domain protein GR4Chr2382 and a reverse transcriptase (RT) gene GR4Chr2383 for which only distant relatives can be found in databases [31].

The G7 and G12 isolates had a potential second chromosomal GI with a site-specific recombinase XerD gene (GR4Chr2824) on the left and a conserved *tRNA-*^*Met*^ gene (GR4Chr2836) on the right, corresponding to the GI insertion site. This GI (hereafter referred to as GI2) is flanked by 50 bp direct repeats (DRs). It extends from positions 2,951,997 to 2,963,666 (~11 kb) and corresponds to two different potential GIs, of 6661 and 7899 bp in length, as predicted by IslandViewer methods. The presence of genes encoding annotated phage-related proteins in GI2 (GR4Chr2831 and GR4Chr2832) raises the possibility of a phage origin, but this region has no known relatives in phage or prophage databases [32].

#### Variation due to mobile elements

Strain GR4 harbors 10 copies of RmInt1, a mobile group II intron that inserts at specific sites within IS*Rm2011-2* (9 copies) and the related IS*Rm10-1* (1 copy), two insertion sequences from the IS630 family with a low splicing efficiency [33]. These insertions would therefore be expected to generate a knockout mutation. We previously reported the presence of an ectopic site for RmInt1 in the *oxi1* gene of *S. meliloti* [20], which is currently annotated as a gene encoding a short-chain dehydrogenase involved in the D-alanine esterification of lipoteichoic acid and wall teichoic acid (GR4pD0623). This insertion was located between positions 689,289 and 689,290 in pSymB, with the intron inserted in the same orientation as the host gene. This intron insertion was found in isolates G4, G8 and G12. We also detected a new ectopic RmInt1 site in the GR4-type genome. The G3 isolate had an additional copy of RmInt1 inserted into the chromosome between positions 1,885,445 and 1,885,446, in a 3-hydroxyisobutyrate dehydrogenase gene (GR4Chr1817). The intron was in the opposite orientation to the host gene. We found no evidence of any other group II intron retrotransposition events in the other GR4-like strains, but the transposition of other mobile (insertion sequences) elements was detected in all three replicons (Additional file 19: Table S12).

## Discussion

We investigated the genome-scale diversity of *S. melioti* species and the early variations occurring during the evolution of these species, by analyzing closely related isolates from a localized population with a well-defined structure. Whole-genome sequencing data at high coverage and SNP analysis further confirmed that the GR4-type isolates were closely related and that most could be clustered into four clades. The mean diversity of the nucleotides segregating in the GR4-type population was very low, and, by contrast to other recent studies on *S. meliloti* and *S. medicae* populations [14, 15], the diversity of the pSymA and pSymB replicons was no higher. Our results suggest that the GR4-type isolates belong to a single cohesive population, with most of the genome following a clonal pattern of evolution.

Different analyses and statistics strongly suggested that the GR4-type population had undergone expansion. Using a strict molecular clock rate of 2.03x10^-8^, we estimated a divergence time for these isolates of ~3.6 Kya, whereas the GR4 strain and closer relatives (clade 1) diverged ~2.4 Kya. It should be noted that the *Sinorhizobium* genus containing *S. fredii* and *S. meliloti* representatives may have diverged [34] around 201-140 million years ago. Bayesian skyline plots with this molecular clock rate timed the expansion of the population to ~2.5–3.0 Kya, with stabilization occurring ~1.25–1.37 Kya. These estimates suggest that the patterns of demographic change observed might be associated with the introduction of alfalfa by the Romans, who acquired it from the Greek civilization in the second century BC and its use as a forage crop during the Roman Empire (27 BC-395 AD). The stabilization of the population may have begun with the reintroduction of alfalfa into Spain by the Moors at the start of the eighth century [24]. Nevertheless, different methods and mutation rates yield very different estimated divergence dates, so caution is required in the interpretation of the estimates obtained.

Despite the low level of nucleotide diversity, we detected SNPs and different types of variation, including indels of different sizes, recombination events, GI excision and the mobility of ISs and group II introns affecting a relatively small, but diverse fraction of the genome. These events appear to have been the early microevolutionary forces shaping the genomes of these isolates.

Interestingly, we identified a pyrimidine/purine dinucleotide CT/GA-rich mid-range inhomogeneity (MRI) region in the isolates, covering 40 to 46 bp and located within an intergenic region of pSymA constituting a specific signature of the genome of this population. Regions of this type may promote the formation of non-canonical DNA conformations (A-DNA or triple-stranded H-DNA) that may lead to local irregularities in DNA structure [27]. This region, which probably originated from a shorter MRI (6 bp) conserved on the chromosome in *S. meliloti* and *S. medicae* may be a variant of ecological relevance, providing adaptation to particular environmental niches [28]. It would therefore be of interest to determine whether this MRI provides the population with any significant phenotypic diversity and whether it is present in other populations coexisting in the same environment and location.

At the sampled field site, the GR4-type population occupied ~49 % of the alfalfa root nodules. In addition, the various genomic variants of the GR4-type isolates are unequally distributed within alfalfa root nodules [20]. The GR4 strain displayed the highest level of nodule occupancy (78 %), suggesting greater fitness for the establishment of symbiotic interactions with the host plant or a higher population size. Strain GR4 is more closely related to the G3 and G6 isolates (clade 1). The G6 isolate has two large regions out of GIs with recombination and replacement signatures in pSymB, whereas the G3 isolate displays an insertion of a group II intron into a 3-hydroxyisobutyrate-dehydrogenase gene (GR4Chr1817) that probably results in gene knockout. In addition, strain GR4 has specific SNPs generating non-synonymous substitutions in coding sequences (Additional file 5: Table S4) or mutations in regulatory elements in intergenic regions (Additional file 6: Table S5). We expect that many of these mutations are neutral due to genetic drift, but it is also plausible that others may cause phenotypic variation and diversification.

In clade 2, the divergence of isolates G4 and G10 from G11 appears to be associated with recombination between the pSymA-*trA1* and pSymB-*trA2* loci. Similarly, in clade 1, the pSymB harbored by isolate G6 has signatures of recombination and replacement over large regions of the genome, possibly acquired by horizontal gene transfer from a different population. Nevertheless, signatures of gene flow and recombination in the GR4-type population are limited, and this population appears to be evolving in a mostly clonal fashion.

Resources in the soil and rhizosphere environments are presumably distributed in patches, as in many other microbial environments [4], probably resulting in the separation of microgeographic niches containing niche-adapted genotypes and gene flow boundaries. Since usually a single bacterium forms a nodule [6], alfalfa root nodules may be considered to be a microhabitat separating the variants, and therefore purging diversity in a periodic selection event. After the nodules senesce bacteria are released to the soil increasing the host plant-adapted genotypes. Thus, the GR4-type population is the outcome of the interplay of genetic drift, microhabitat separation low levels of gene flow, and strong selection by the host plant.

This work on the GR4-type population and further studies on distinct genotypic clusters co-existing at the same site (e.g. the EM2-type population) will provide insight into genome evolution and ecological differentiation in *S. meliloti* populations.

## Conclusions

When trying to interpret estimated diversity and to uncover the early events contributing to ecological differentiation, it is important to sample closely related isolates from the same geographic location. We sequenced a new clone of *Sinorhizobium meliloti* strain GR4, a nitrogen-fixing bacterium that is highly competitive for alfalfa nodulation. We used Illumina technology to sequence the genomes of 13 *S. meliloti* isolates from the same field site, representative of genomic variation within the GR4-type population. We determined nucleotide diversity, divergence times, demographic history, polymorphism and genomic variation within the population. Significant genomic variation was observed in this population despite the low nucleotide diversity of the three major replicons harbored by the GR4-type isolates, the chromosome, pSymB and pSymA. Our results suggest that this is a single cohesive highly clonal population, and that it probably arose from and is maintained by genetic drift, microhabitat separation, low-level gene flow and plant host selection. These findings contribute to understand early genome evolution in *S. meliloti* populations that may have played an important role in diversification and environmental adaptation.

## Methods

### Bacteria used in this study

*S. meliloti* strain GR4, originally obtained from nodules on *M. sativa* grown at a field site at the Estación Experimental del Zaidín [18], and 21 isolates previously obtained from nodules on *M. sativa* grown at the same field site and representative of the genomic variation within the GR4-type population [20] were fingerprinted with IS*Rm2011-2* and the group II intron RmInt1 as DNA probes (Additional file 1: Figure S1). Bacteria were grown on complete TY medium at 28 °C and used for DNA extraction. Aliquots of DNA were digested with *Eco*RI and subjected to electrophoresis in 1 % Tris-borate agarose gels. The DNA was then vacuum blotted onto positively charged nylon membranes (Roche Diagnostics). The DNA probes used for DNA fingerprinting were based on IS*Rm2011-2* and the group II intron RmInt1 and have been described elsewhere [19]. The GR4-type isolates corresponding to 13 variants of the GR4 fingerprint pattern (3G48, 3D13, 3 F11, 7D33, 5G35, 5 F20, 7G54, 2B2, 1A66, 7A75, 5D25, 1B5 and 2A8, referred to as G1 to G13, respectively, for the sake of simplicity) were chosen for further studies.

### Genome sequencing and SNP calls

The GR4 strain and the isolates were sequenced by Macrogen Inc. (South Korea), with Illumina paired-end technology, using multiplex MiSeq run (2x300 bp). Quality scores Q ≥ 30 (probability of incorrect base calls: 1 in 1000) were obtained for 76.36 to 79.31 % of the bases. For correction of the GR4 reference genome, Illumina reads from the clone of the GR4 strain sequenced were mapped separately to each replicon with Geneious Pro Software v 8.0 (Biomatters Ltd; http://www.geneious.com [35]): the chromosome (NC_019845), pSymB (NC_019849), pSymA (NC_019848), and accessory replicons pRmeGR4b (NC_019847) and pRmeGR4a (NC_019846). Mapping was carried out with minimum identity overlaps of 100, 99 and 90 % over up to five iterations.

A single paired-reads file was created before alignment. For the detection of single-nucleotide polymorphisms (SNPs)/variants, the reads for each of the GR4-type isolates were mapped to each of the replicons of the curated sequenced GR4 reference strain, with a minimum identity overlap of 99 % and trim paired read overhangs. A SNP was called at a site only if that site had a minimum coverage of at least six reads, a maximum variant *P*-value of 6x10^-6^ (0.0001 % chance of seeing the variant by chance), a minimum strand-bias *P*-value of 5x10^-5^ when exceeding 65 % bias, and if the nucleotide concerned was found in ≥95 % of unique reads. We inspected the genome manually and excluded SNPs/variants due to repeated elements and regions that displayed signatures of recombination (high levels of SNP accumulation). We estimated the expected non-synonymous/synonymous polymorphism (nsSNPs/sSNPs) ratio from concatenated CDSs for the chromosome (3254), pSymB (1490) and pSymA (1216), with MEGA 6.0 software [36], and calculated 0-fold, 2-fold and 4-fold degenerate sites as previously described [37].

### Sequence alignments and phylogenetic analyses

MAFFT was used for sequence alignment, with the scoring matrix 200PAM/K = 2. Phylogenetic trees for SNP analyses were inferred by Bayesian analysis, with the parallel version of MrBayes 3.1 [22] implemented in the Geneious program, using the HKY85 substitution model with gamma correction of between-site rate variation for four rate categories. Two independent runs of four chains were completed for 1,100,000 Metropolis-coupled Markov chain Monte Carlo (MCMC) generations, using the default priors for model parameters. Trees were sampled every 200 generations, and 100,000 samples were discarded as the “burn-in” to produce a 50 % majority-rule consensus tree. The neighbor-joining algorithm was used, with the HKY substitution model and 1000 bootstrap replicates, to establish the relationships between pSymA and pSymB *trA* loci and to infer a signature of genetic recombination events in some of the isolates.

### Statistical analyses and divergence time of the isolates

Tajima’s *D* test of neutrality for concatenated CDS was performed with MEGA 6.0 software [36, 38, 39] and DnaSP v5.10.01 [40]. Fu and Li’s D*, Fu and Li’s F*, Fu’s *F*_*s*_ statistics [41, 42], and mismatch distribution analysis were computed with DnaSP v5.10.01. For the mismatch distribution analysis, a model of population growth/decline for expected values was fitted to the data for estimating of the time at which population expansion occurred. The model has three parameters: scaled mutation rates Theta (θ) initial, θ final, and Tau (τ), the time since population expansion measured in units of mutational time (τ = 2*υt*, where *t* is the time in generations, and *υ* is the mutation rate per sequence and per generation; [43, 44]). Rhizobia in the soil may have a generation time of about 200 h per generation, with about 44 generations per year [45]. Assuming a mutation rate of 2.03x10^-8^ per site per year, which is equivalent to a universal mutation rate of 0.0033 per genome per generation, as proposed by Drake et al. [46], the time since expansion was calculated with the formula *t* = τ/2*υL*, where *L* is the length of the concatenated sequence in bp.

BEAST 2 software [23] was used for MCMC analysis on concatenated CDSs carrying both sSNPs and nSNPs. The Bayesian skyline plot (BSP) implemented in BEAST [47, 48] uses a Bayesian coalescent inference of phylogeny and a MCMC algorithm for the simultaneous estimation of a posterior probability distribution for the ancestral genealogy, branch lengths, substitution model parameters, and population parameters over time. The resulting BSP shows the credibility interval for effective population size. We applied a HKY substitution model with gamma correction of between-site rate variation for four rate categories and a strict molecular clock rate of 2.03x10^-8^ per site per year. For the tree prior we used a coalescent Bayesian skyline plot. Each MCMC sample was based on a run of 10,000,000 generations, sampled every 1000 generations, with the first 1,000,000 generations discarded as burn-in. This analysis was carried out twice. We used Tracer v1.6 to analyze the Bayesian runs, to confirm that there was a suitable effective sample size (ESS) for all parameters estimated from the posterior distribution of the trees (i.e. ESS values were greater than 200; [48]), with confirmation of the stationary state of each chain following the removal of a suitable number of burn-in runs (10 %) and convergence of the runs. The trees obtained from the runs were combined, with LogCombiner, and TreeAnnotator, from BEAST 2 software, to summarize the tree output file, obtaining a maximum clade credibility tree with a 10 % burn-in and median branch lengths and their standard deviations. Trees were visualized with Figtree v1.4.2 (tree.bio.ed.ac.uk).

### Functional analyses of CDSs carrying SNPs

Associations of Gene Ontology (GO) terms with concatenated CDS carrying sSNPs and nsSNPs were identified. This method describes gene products in terms of the biological processes, cellular components and molecular functions with which they are associated, in a species-independent manner [49].

### Detection of genomic variants

Regions of the GR4 genome not covered by reads from the various isolates with a minimum identity overlap of 99 % were further investigated, using a minimum identity overlap of 90 %. Mapped reads often include sequences at their ends that do not map to the reference genome, a signature of genomic variation in these particular regions. BLAST searches of non-redundant databases with these sequences not aligned at the boundaries of the uncovered region identified the nature of the genomic variation: large indels caused by mobile genetic elements or GI excision. Recombination events were identified on the basis of an accumulation of SNPs, which were further confirmed in some cases by phylogenetic analyses. Insertion sequences were further identified with the ISfinder database (https://www-is.biotoul.fr/).

## Additional files

Additional file 1: Figure S1. Dendrograms based on GR4-type isolate DNA fingerprints constructed by the UPGMA method. a Based on the group II intron RmInt1 fingerprint. b Based on the IS*Rm2011-2* fingerprint. DNA fingerprint images were acquired with Gel Doc 1000/2000 (Bio-Rad), and the patterns were analyzed with Quantity One software (Bio-Rad), using a similarity level of 70 %. GR4-type isolates are boxed and their names are indicated. The 13 isolates sequenced are labeled G1 to G13. Isolates 5 F10 and 6E9 belong to a different population (EM2) from the same field site. (TIF 2306 kb) Additional file 2: Table S1. Updating of the GR4 genome sequence data on the basis of Illumina reads. (DOC 129 kb) Additional file 3: Table S2. Reads mapped to the GR4 strain reference genome. (PDF 46 kb) Additional file 4: Table S3. sSNP string sequence. (PDF 74 kb) Additional file 5: Table S4. nsSNP string sequence. (PDF 100 kb) Additional file 6: Table S5. iSNP string sequence. (PDF 283 kb) Additional file 7: Figure S2. Functional analysis of genes carrying non-synonymous and synonymous SNPs. a nsSNPs. b sSNPs. Genes containing sSNPs or nsSNPs were separated into functional categories to determine the relationships between gene function and potential SNPs by Gene Ontology analyses. Graph level 3 pie charts are shown. (TIF 1418 kb) Additional file 8: Figure S3. Nucleotide alignment of concatenated SNPs. The consensus sequence is shown above the alignment, with a threshold of 75 %. Mean pairwise identity over all pairs in the column is also shown below the consensus. Green: 100 % identity, khaki: at least 30 % identity but less than 100 % identity, Red: less than 30 % identity. Differences from the consensus are highlighted with Clustal colors. (TIF 2136 kb) Additional file 9: Table S6. Concatenated genes carrying sSNPs. (PDF 72 kb) Additional file 10: Table S7. Concatenated genes carrying nsSNPs. (PDF 77 kb) Additional file 11: Table S8. Deletions (4-63 nts) in the GR4-type isolates. (PDF 66 kb) Additional file 12: Table S9. Small indels in the chromosome of the GR4-type isolates. (PDF 66 kb) Additional file 13: Table S10. Small indels in the pSymB of the GR4-type isolates. (PDF 58 kb) Additional file 14: Table S11. Small indels in the pSymA of the GR4-type isolates. (PDF 58 kb) Additional file 15: Figure S4. Neighbor-joining phylogenetic tree for the pSymA-*trA1* and pSymB-*trA2* of GR4-type isolates. The phylogenetic tree is based on the sequence alignment of the GR4-type isolate and strain GR4 *trA1* and *trA2* gene sequences. The corresponding loci harbored by *S. meliloti* strain 2011 were included as the outgroup. Bootstrap values are indicated at the nodes. For a better visualization, nodes corresponding to GR4-type pSymA-*trA1* and pSymB-*trA2* sequences were collapsed. (TIF 553 kb) Additional file 16: Figure S5. Alignment of repeat sequences. Strain GR4 pSymA IGR positions 1,116,938 to 1,117,089 (repeat 1) and 1,145,069 to 1,145,218 (repeat 2) and those identified in G4 and G10 isolates. Discordant nucleotide positions are highlighted. (PDF 210 kb) Additional file 17: Figure S6. Mapping of G6 reads to the reference GR4 pSymB. a Region spanning 48 kb (800,349 to 848,330) and displaying an accumulation of SNPs (labeled in blue below the region). b Region encompassing almost 200 kb (1,098,191 to 1,296,315), showing the missing areas. (PDF 350 kb) Additional file 18: Figure S7. Excised genomic islands and the CDSs they contain. a Island GI1. b Island GI2. The direct repeats and the tRNAmet sequences identified are indicated. (PDF 617 kb) Additional file 19: Table S12. Variants due to mobile elements in the GR4-type isolates. (PDF 77 kb)
