# Supplementary figures and images for: The early events underlying genome evolution in a localized Sinorhizobium meliloti population
Source: BMC Genomics. 2016 Aug 5;17:556. doi: 10.1186/s12864-016-2878-9 (PMC4974801; doi:10.1186/s12864-016-2878-9)

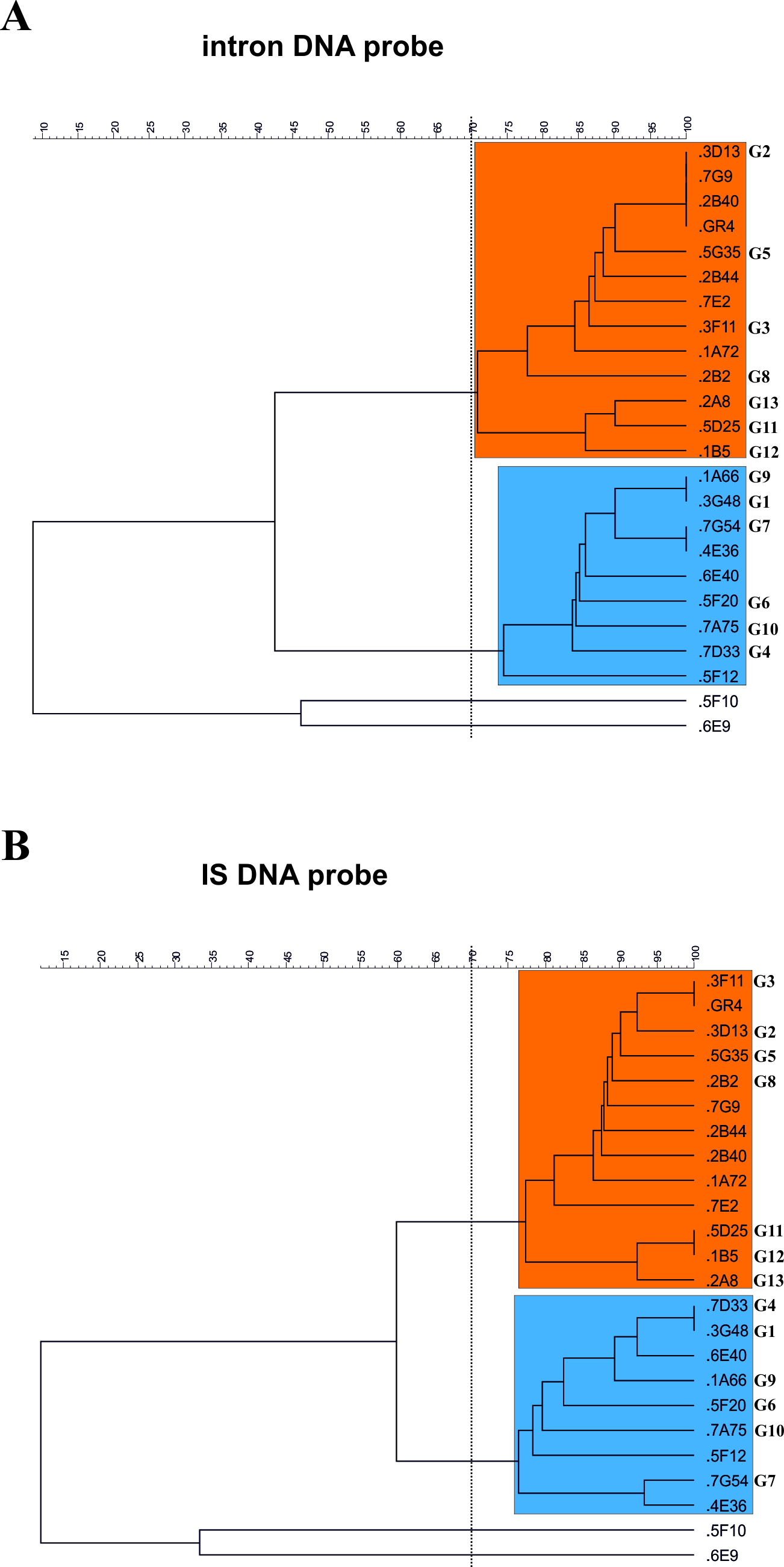

Supplement: Additional file 1: Figure S1. — Dendrograms based on GR4-type isolate DNA fingerprints constructed by the UPGMA method. a Based on the group II intron RmInt1 fingerprint. b Based on the ISRm2011-2 fingerprint. DNA fingerprint images were acquired with Gel Doc 1000/2000 (Bio-Rad), and the patterns were analyzed with Quantity One software (Bio-Rad), using a similarity level of 70 %. GR4-type isolates are boxed and their names are indicated. The 13 isolates sequenced are labeled G1 to G13. Isolates 5 F10 and 6E9 belong to a different population (EM2) from the same field site. (TIF 2306 kb) [file 12864_2016_2878_MOESM1_ESM.tif]

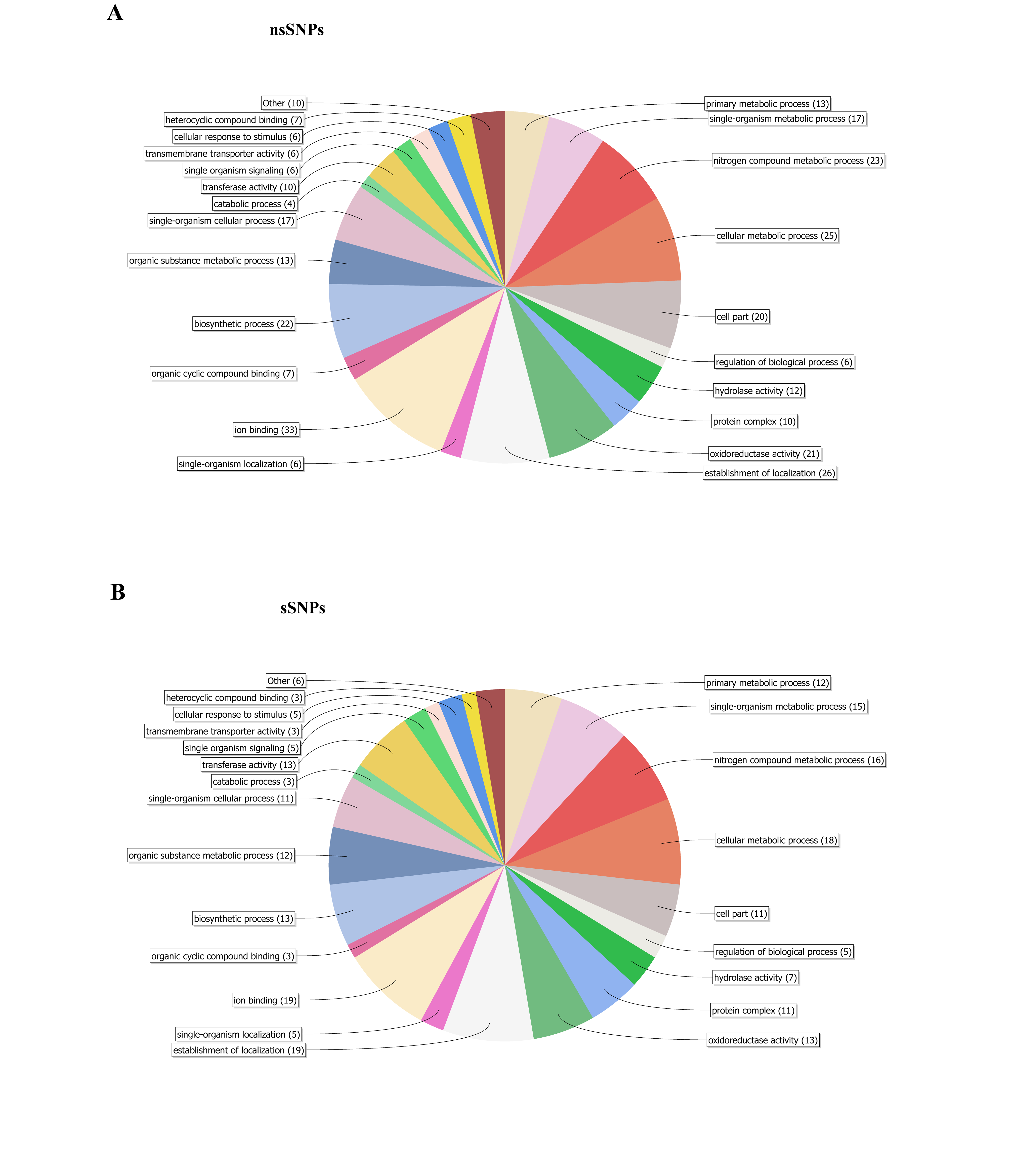

Supplement: Additional file 7: Figure S2. — Functional analysis of genes carrying non-synonymous and synonymous SNPs. a nsSNPs. b sSNPs. Genes containing sSNPs or nsSNPs were separated into functional categories to determine the relationships between gene function and potential SNPs by Gene Ontology analyses. Graph level 3 pie charts are shown. (TIF 1418 kb) [file 12864_2016_2878_MOESM7_ESM.tif]

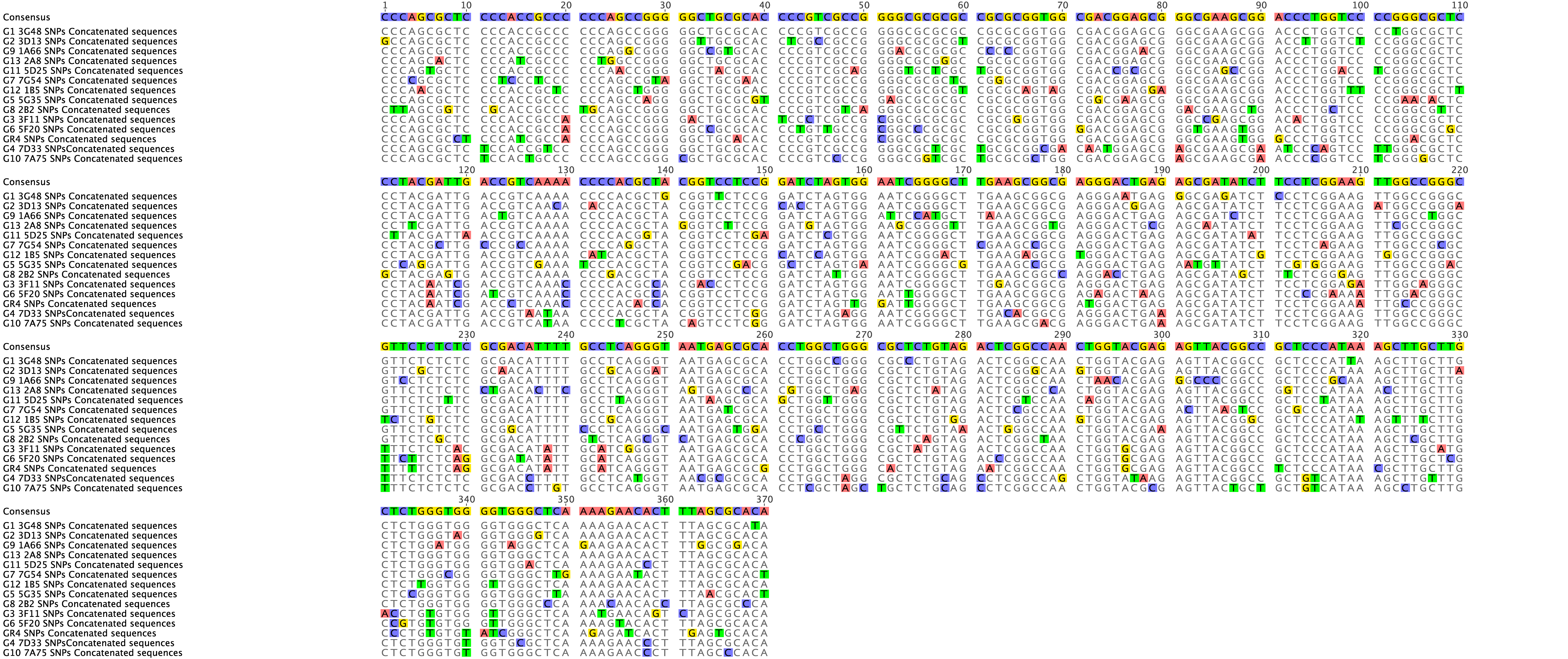

Supplement: Additional file 8: Figure S3. — Nucleotide alignment of concatenated SNPs. The consensus sequence is shown above the alignment, with a threshold of 75 %. Mean pairwise identity over all pairs in the column is also shown below the consensus. Green: 100 % identity, khaki: at least 30 % identity but less than 100 % identity, Red: less than 30 % identity. Differences from the consensus are highlighted with Clustal colors. (TIF 2136 kb) [file 12864_2016_2878_MOESM8_ESM.tif]

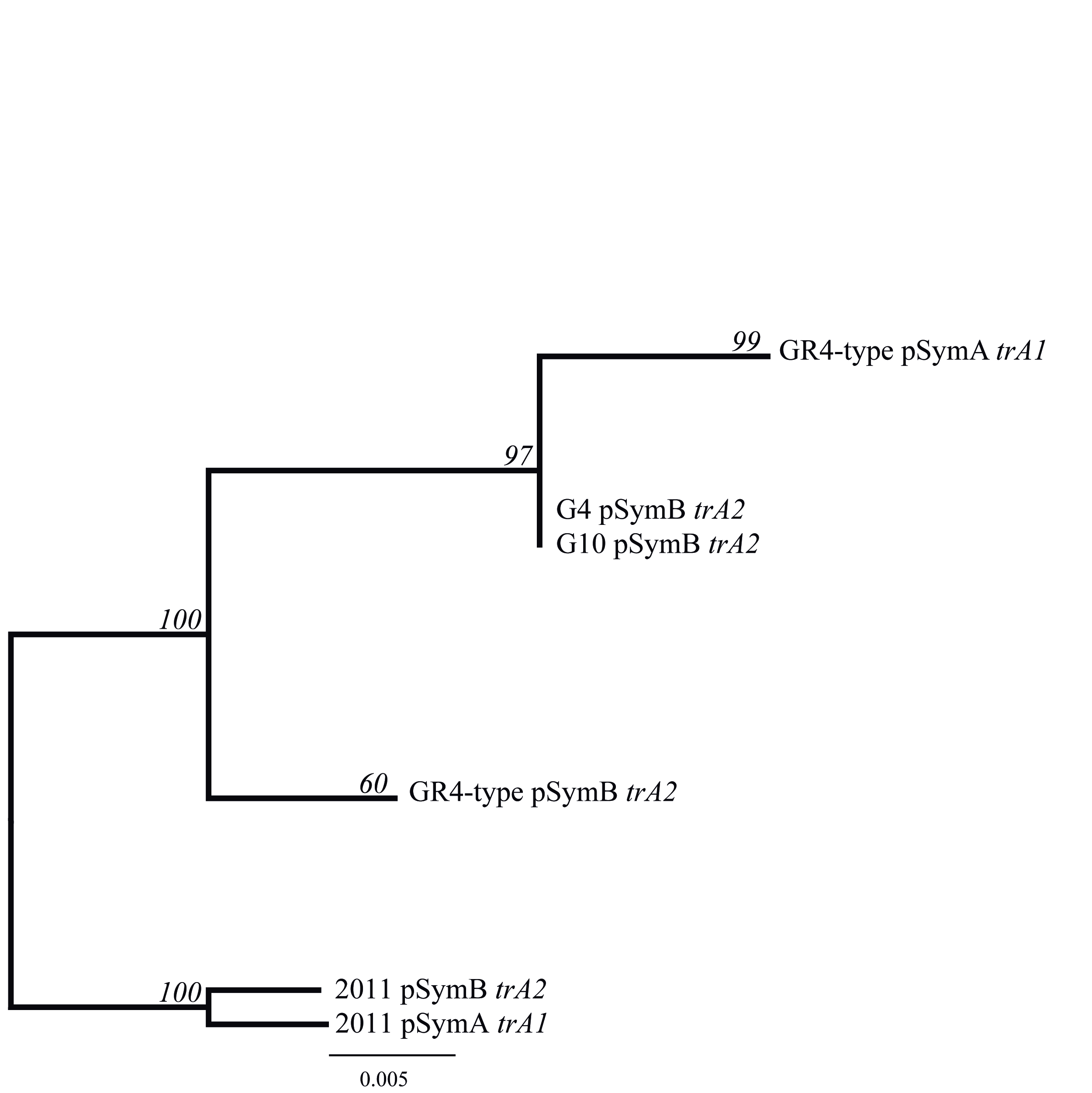

Supplement: Additional file 15: Figure S4. — Neighbor-joining phylogenetic tree for the pSymA-trA1 and pSymB-trA2 of GR4-type isolates. The phylogenetic tree is based on the sequence alignment of the GR4-type isolate and strain GR4 trA1 and trA2 gene sequences. The corresponding loci harbored by S. meliloti strain 2011 were included as the outgroup. Bootstrap values are indicated at the nodes. For a better visualization, nodes corresponding to GR4-type pSymA-trA1 and pSymB-trA2 sequences were collapsed. (TIF 553 kb) [file 12864_2016_2878_MOESM15_ESM.tif]

**A**

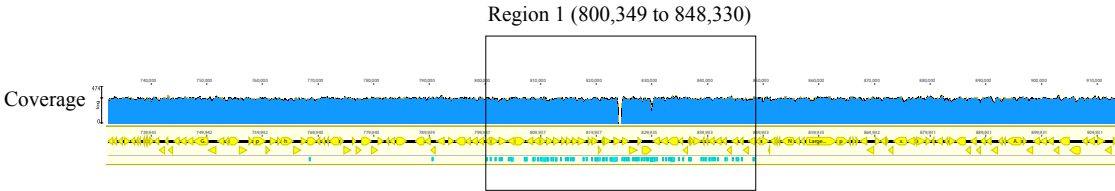

**B**

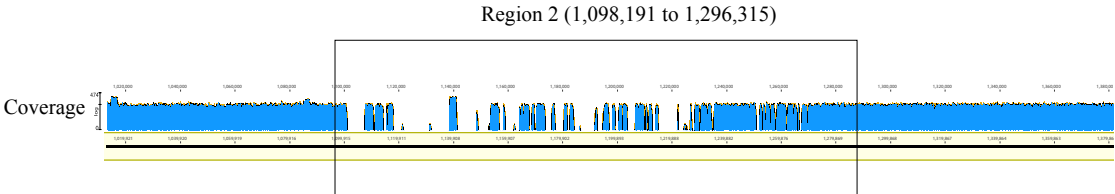

Supplement: Additional file 17: Figure S6. — Mapping of G6 reads to the reference GR4 pSymB. a Region spanning 48 kb (800,349 to 848,330) and displaying an accumulation of SNPs (labeled in blue below the region). b Region encompassing almost 200 kb (1,098,191 to 1,296,315), showing the missing areas. (PDF 350 kb) [file 12864_2016_2878_MOESM17_ESM.pdf]

A

## Genomic island GI1

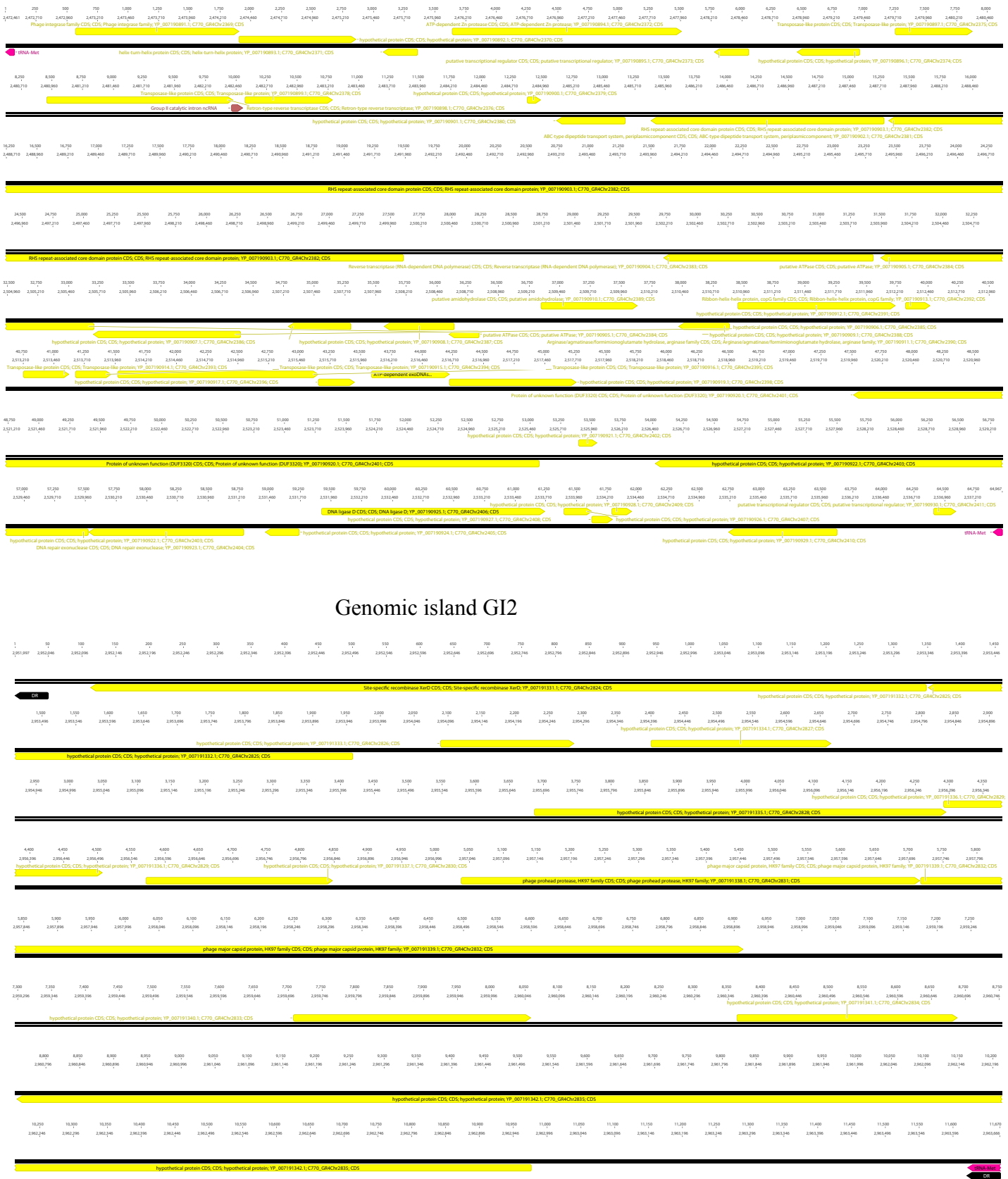

B

## Genomic island GI2

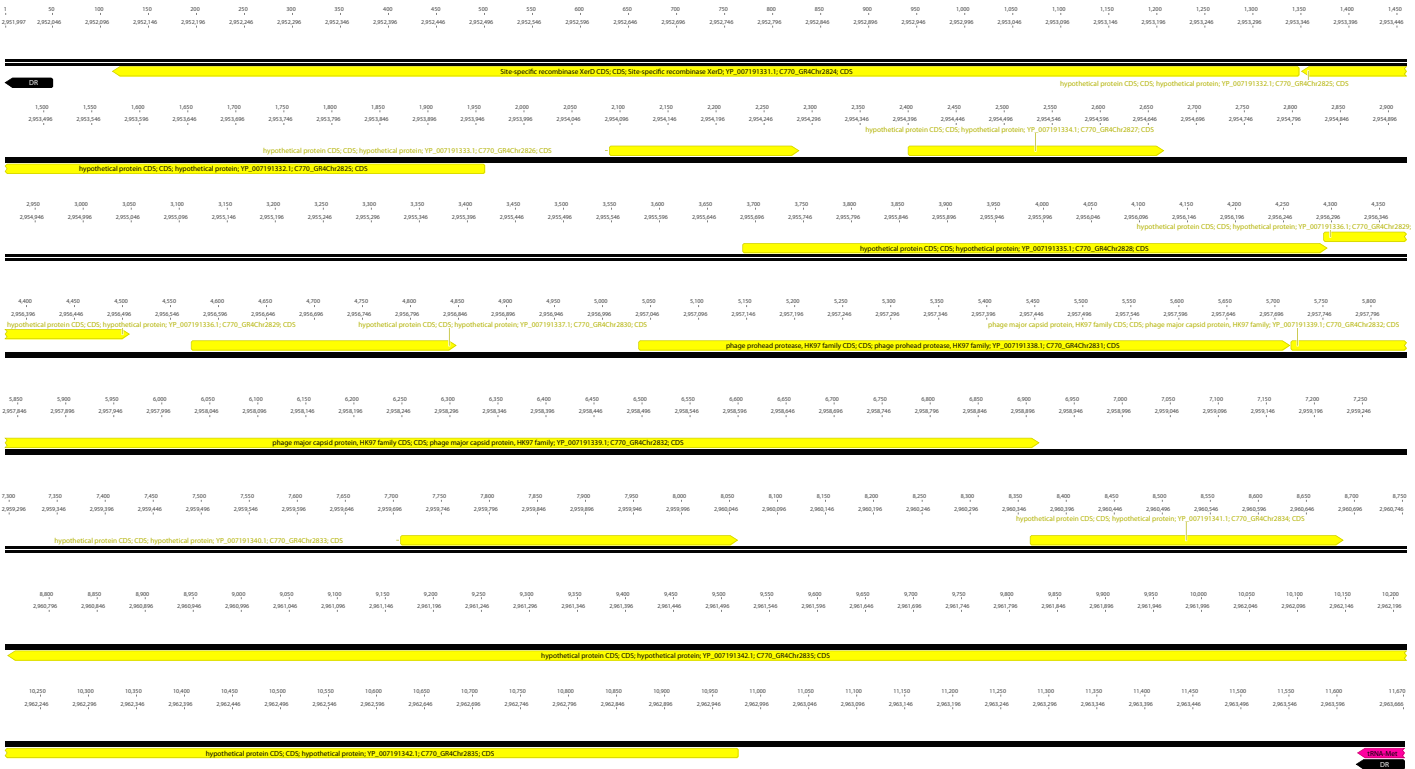

Supplement: Additional file 18: Figure S7. — Excised genomic islands and the CDSs they contain. a Island GI1. b Island GI2. The direct repeats and the tRNAmet sequences identified are indicated. (PDF 617 kb) [file 12864_2016_2878_MOESM18_ESM.pdf]
