# Supplementary material for: The early events underlying genome evolution in a localized Sinorhizobium meliloti population
Source: BMC Genomics. 2016 Aug 5;17:556. doi: 10.1186/s12864-016-2878-9 (PMC4974801; doi:10.1186/s12864-016-2878-9)
Supplement: Additional file 2: Table S1. — Updating of the GR4 genome sequence data on the basis of Illumina reads. (DOC 129 kb) [file 12864_2016_2878_MOESM2_ESM.doc]

**S1 Table. Specific changes for updating the GR4 genome sequence data based on Illumina reads**

| Replicon^a^ |  | Disagreement^b^ |  |  | affected gene(s) | Notes | Accs number |
| --- | --- | --- | --- | --- | --- | --- | --- |
|  | characteristic | Coordinates | Type | size |  |  |  |
|  |  |  |  |  |  |  |  |
| **Chromosome**  3,618,794 (3,620,713) |  |  |  |  |  |  | NC_019845.1 |
|  | Assembler error | 295,056-295,063 | Ins | 1533 | Intergenic: Chr0272 // Chr0273 | ‘NNNNNNNN’ are substituted by a new Trm20 copy |  |
|  | 454 reads error | 824,693-824,694 | Ins | 1 | Chr0776-Chr0777 | ‘TTTTTT’ are transformed to ‘TTTTTTT’of the ribosomal slippage of transposase IS*Rm2011-2* |  |
|  | Assembler error | 1,984,370-1,984,375 | Ins | 221 | Intergenic: Chr1903 // Chr1904 | ‘NNNNNN’ are substituted by 221 nt |  |
|  | 454 reads error | 2,247,687-2,247,688 | Ins | 1 | Intergenic: Chr2165 // Chr2166 | ‘GGG’ are transformed to ‘GGGG’ |  |
|  | 454 reads error | 2,795,705-2,795,715 | Ins | 1 | Intergenic: Chr2668 // Chr2669 | CCCCCCCCCCC are transformed to CCCCCCCCCCCC |  |
|  | Assembler error | 3,094,384-3,094,385 | Ins | 183 | Intergenic: Chr2956 // Chr2957 | -- |  |
|  | 454 reads error | 3,250,569-3,250,570 | Ins | 1 | Intergenic: Chr3099 // Chr3100 | ‘TGC’ are substituted by ‘TGGC’ |  |
|  | 454 reads error | 3,267,286 | Repl | - | Chr3116 | ‘N’ is a ‘A’ defining a Val |  |
|  |  |  |  |  |  |  |  |
| **pRmeGR4d (pSymB)**  1,701,197 (1,701,381) |  |  |  |  |  |  | NC_019849.1 |
|  | 454 reads error | 329,064-329,065 | Ins | 1 | Intergenic: pD0295 // pD0296 | a ‘G’ |  |
|  | Assembler error | 666,709-666,710 | Ins | 182 | Intergenic: pD0605 // pD0606 | -- |  |
|  | 454 reads error | 1,6784,27 | Del | -1 | Intergenic: pD1530 // pD1531 | N is - |  |
|  | Assembler error | 1,681,964-1,681,965 | Ins | 3 | pD1536 | a ‘TGT’ complete the 3’ end of a group II Intron catalytic ncRNA |  |
|  | Assembler error | 1,683,869 | Del | -1 | Intergenic: pD1539 // pD1540 | N is - |  |
|  |  |  |  |  |  |  |  |
| **pRmeGR4c (pSymA)**  1,417,856 (1,417,907) |  |  |  |  |  |  | NC_019848.1 |
|  | Assembler error | 134,812 | Repl | - | Intergenic pC0125//pC0124 | ‘T’ is a ‘G’; a disrupted group II Intron catalytic ncRNA is involved |  |
|  | 454 reads error | 732,255-732,256 | Ins | 1 | pC0727 (frameshift gene) | ‘AA’ are ‘AAA’, putative *FixK*1 gene is now correct in GR4; another copy exist in pC1072 |  |
|  | Assembler error | 947489 | Repl | - | pC0962 | ‘T’ is a ‘C’; synonymous change in a Ti-type conjugative transfer relaxase TraA |  |
|  | Assembler error | 1,117,469-1,118,323 | Repl | 854 | pC1149-pC1150 | 854 nt are assembled in the inverted position. Generate a truncated conserved new gene of encoding a ‘mechanosensitive ion channel protein MscS’(384 aa) |  |
|  | Assembler error | 1,153,976-1,154,830 | Repl | 854 | pC1178-pC1179 | 854 nt are assembled in the inverted position. Generate a conserved new gene of encoding a ‘mechanosensitive ion channel protein MscS’ (694 aa) |  |
|  | Assembler error | 1203565 | Repl | - | pC1229 | ‘N’ is a ‘T’ defining an Ala in a Transposase-like protein, IS5 family |  |
|  |  |  |  |  |  |  |  |
| **pRmeGR4b**  225,725 (225,725) |  |  |  |  |  |  | NC_019847.1 |
|  | Assembler error | 77,726 | Repl | - | pB089 | ‘A’ is a ‘G’ synonymous change in chaperonin GroL |  |
|  | Assembler error | 77,734 | Repl | - | pB089 | ‘T’ is a ‘C’ synonymous change in chaperonin GroL |  |
|  | Assembler error | 77,869 | Repl | - | pB089 | ‘C’ is a ‘G’ Glu change to Gln in chaperonin GroL |  |
|  | Assembler error | 177,675-177,679 | Repl | 1 | pB221 | ‘ACCG’ are ‘CCGA’, Ala-Val change to Val-Gly in a site-specific recombinase, DNA invertase Pin-like protein |  |
|  |  |  |  |  |  |  |  |
| **pRmeGR4a**  175,986 (175,983) |  |  |  |  |  |  | NC_019846.1 |
|  | Assembler error | 3,033 | Repl | - | pA004 | ‘G’ is ‘T’; a Ser change to Ile in the replication protein C |  |
|  | Assembler error | 67,390 | Repl | - | pA060 | ‘G’ is ‘A’; synonymous change in RecA-superfamily ATPases |  |
|  | Assembler error | 67,499 | Repl | - | pA060 | ‘G’ is ‘T’; a Tyr change to Ser in RecA-superfamily ATPases |  |
|  | Assembler error | 67,525 | Repl | - | pA060 | ‘G’ is ‘C’; synonymous change in RecA-superfamily ATPases |  |
|  | Assembler error | 67,579 | Repl | - | pA060 | ‘G’ is ‘T’; synonymous change in RecA-superfamily ATPases |  |
|  | Assembler error | 67,592 | Repl | - | pA060 | ‘C’ is ‘G’; a Gly change to Ala in RecA-superfamily ATPases |  |
|  | Assembler error | 67,669 | Repl | - | pA060 | ‘G’ is ‘C’; synonymous change in RecA-superfamily ATPases |  |
|  | Assembler error | 67,671-67,672 | Repl | - | pA060 | ‘TC’ is ‘CT’; a Thr change to Ala in RecA-superfamily ATPases |  |
|  | Assembler error | 67,978 | Repl | - | pA060 | ‘G’ is ‘A’; synonymous change in RecA-superfamily ATPases |  |
|  | 454 reads error | 68,292-68,293 | Ins | 1 | Intergenic pA060//pA061 | ‘TTT’ is ‘TTTT’ |  |
|  | Assembler error | 68,638 | Repl | - | pA061 | ‘C’ is ‘T’; synonymous change in K+ transporter CDS |  |
|  | Assembler error | 68,849-6851 | Repl | - | pA061 | ‘CAC’ is ‘GAA’; a Asp change to Glu in K+ transporter CDS |  |
|  | Assembler error | 68,992 | Repl | - | pA061 | ‘T’ is ‘C’; synonymous change in K+ transporter CDS |  |
|  | Assembler error | 69,124 | Repl | - | pA061 | ‘C’ is ‘T’; synonymous change in K+ transporter CDS |  |
|  | Assembler error | 69,136 | Repl | - | pA061 | ‘G’ is ‘A’; synonymous change in K+ transporter CDS |  |
|  | Assembler error | 69,151 | Repl | - | pA061 | ‘T’ is ‘C’; synonymous change in K+ transporter CDS |  |
|  | Assembler error | 69,177 | Repl | - | pA061 | ‘C’ is ‘T’; synonymous change in K+ transporter CDS |  |
|  | Assembler error | 69,193 | Repl | - | pA061 | ‘C’ is ‘A’; synonymous change in K+ transporter CDS |  |
|  | Assembler error | 69,517 | Repl | - | pA061 | ‘T’ is ‘C’; synonymous change in K+ transporter CDS |  |
|  | Assembler error | 69,568 | Repl | - | pA061 | ‘C’ is ‘T’; synonymous change in K+ transporter CDS |  |
|  | Assembler error | 69,676 | Repl | - | pA061 | ‘T’ is ‘C’; synonymous change in K+ transporter CDS |  |
|  | Assembler error | 69,739 | Repl | - | pA061 | ‘C’ is ‘G’; a Ile change to Met in K+ transporter CDS |  |
|  | Assembler error | 69,785 | Repl | - | pA061 | ‘A’ is ‘G’; a thr change to Ala in K+ transporter CDS |  |
|  | Assembler error | 69,823 | Repl | - | pA061 | ‘G’ is ‘A’; a Met change to Ile in K+ transporter CDS |  |
|  | Assembler error | 69,997 | Repl | - | pA061 | ‘T’ is ‘C’; synonymous change in K+ transporter CDS |  |
|  | Assembler error | 129,619 | Repl | - | pA138* | ‘G’ is ‘C’; |  |
|  | Assembler error | 129,649 | Repl | - | pA138* | ‘G’ is ‘A’; |  |
|  | Assembler error | 129,961-129,964 | Del | -4 | pA138* | Generate a frameshift in a transposase |  |
|  | Assembler error | 130,094 | Repl | - | pA138 | ‘G’ is ‘C’; a Ser change to Cys in a transposase |  |
|  | Assembler error | 130,141 | Repl | - | pA138 | ‘T’ is ‘C’; synonymous change in a transposase |  |
|  | Assembler error | 130,257 | Repl | - | pA138 | ‘A’ is ‘G’; a Ser change to Pro in a transposase |  |
|  | Assembler error | 130,645 | Repl | - | pA138 | ‘G’ is ‘C’; synonymous change in a transposase |  |
|  | Assembler error | 130,728 | Repl | - | Intergenic pA138//pA139 | ‘T’ is ‘C’; |  |
|  | Assembler error | 130,762 | Repl | - | Intergenic pA138//pA139 | ‘C’ is ‘T’; |  |
|  | Assembler error | 174,534-174,543 | Del | -10 | Intergenic pA184//pA185 | -- |  |
|  | Assembler error | 174,667-174,668 | Ins | 10 | Intergenic pA184//pA185 | ‘CCGGCTCTTT’ |  |
|  |  |  |  |  |  |  |  |

^a^Size of the annotated replicon (size update) in bp is indicated

^b^Sequence disagreements (based on 454 errors or assembler errors) were corrected based on pairwise mapping of Illumina reads at 100% of identity on reference GR4 genome and revised on Newbler assembler of 454 reads sequence data (Ins: insertion; Del: deletion; Repl: replacement).
