# Supplementary material for: The early events underlying genome evolution in a localized Sinorhizobium meliloti population
Source: BMC Genomics. 2016 Aug 5;17:556. doi: 10.1186/s12864-016-2878-9 (PMC4974801; doi:10.1186/s12864-016-2878-9)
Supplement: Additional file 3: Table S2. — Reads mapped to the GR4 strain reference genome. (PDF 46 kb) [file 12864_2016_2878_MOESM3_ESM.pdf]

S2 Table. Mapped reads

Strain/isolates	Replicon	Mapped reads	% of mapped reads	Mean reads coverage	Ref-Seq coverage %
	Chromosome	891.632,00	46,37	70,70	99.999
	pSymB	374.379,00	19,47	60,00	100.000
GR4	pSymA	295.180,00	15,35	63,20	100.000
	pRmeGR4a	12.633,00	0,66	20,70	100.000
	pRmeGR4b	26.672,00	1,39	34,10	100.000
x80	Total	1.922.866,00			
	Chromosome	820.589,00	38,50	68,10	99.996
	pSymB	355.197,00	16,67	62,70	99.999
G1	pSymA	309.279,00	14,51	65,50	99.998
	pRmeGR4a	4.492,00	0,21		20,5
	pRmeGR4b	40.614,00	1,91	54,00	100.000
x89	Total	2.131.222,00			
	Chromosome	847.810,00	38,70	70,40	99.998
	pSymB	364.561,00	16,64	64,40	99.996
G2	pSymA	312.463,00	14,26	66,20	99.999
	pRmeGR4a	4.323,00	0,20		20,2
	pRmeGR4b	30.915,00	1,41	41,10	100.000
x92	Total	2.190.878,00			
	Chromosome	672.917,00	30,58	55,90	99.998
	pSymB	272.883,00	12,40	48,20	99.998
G3	pSymA	229.545,00	10,43	48,70	99.600
	pRmeGR4a	1.666,00	0,08		4,7
	pRmeGR4b	23.058,00	1,05	30,70	100.000
x92	Total	2.200.748,00			
	Chromosome	887.002,00	38,42	73,60	99.997
	pSymB	387.906,00	16,80	68,50	100.000
G4	pSymA	332.069,00	14,38	70,40	99.990
	pRmeGR4a	5.061,00	0,22		19,4
	pRmeGR4b	36.727,00	1,59	48,90	100.000
x97	Total	2.308.700,00			
	Chromosome	797.256,00	41,65	66,20	98.400
	pSymB	314.614,00	16,44	55,60	99.998
G5	pSymA	260.340,00	13,60	55,20	99.997
	pRmeGR4a	2.273,00	0,12		9,1
	pRmeGR4b	15.540,00	0,81	20,40	60
x80	Total	1.914.034,00			
	Chromosome	939.502,00	40,51	77,80	99.999
	pSymB	356.404,00	15,37	62,80	95.200
G6	pSymA	322.246,00	13,90	68,20	99.999
	pRmeGR4a	2.362,00	0,10		5,5
	pRmeGR4b	32.134,00	1,39	42,70	100.000
x97	Total	2.319.108,00			
	Chromosome	585.172,00	36,25	48,50	99.700
	pSymB	248.318,00	15,38	43,80	99.990
G7	pSymA	214.492,00	13,29	45,40	99.998
	pRmeGR4a	9.264,00	0,57	15,80	81.100
	pRmeGR4b	20.416,00	1,26	27,10	99.960
x68	Total	1.614.110,00			
	Chromosome	923.750,00	40,67	76,60	99.998
	pSymB	373.255,00	16,43	65,90	99.999
G8	pSymA	312.355,00	13,75	66,10	99.995
	pRmeGR4a	3.771,00	0,17		18,8
	pRmeGR4b	32.014,00	1,41	42,60	99.996
x95	Total	2.271.112,00			
	Chromosome	646.724,00	39,43	53,60	99.990
	pSymB	262.274,00	15,99	46,30	99.996
G9	pSymA	226.362,00	13,80	47,90	99.997
	pRmeGR4a	1.583,00	0,10		4,6
	pRmeGR4b	19.991,00	1,22	26,60	100.000
x69	Total	1.640.058,00			
	Chromosome	484.783,00	37,32	40,20	99.990
	pSymB	203.981,00	15,70	36,00	99.980
G10	pSymA	185.582,00	14,29	39,30	99.990
	pRmeGR4a	3.474,00	0,27		33,1
	pRmeGR4b	17.702,00	1,36	23,50	100.000
x55	Total	1.299.000,00			
	Chromosome	698.520,00	39,58	57,90	99.990
	pSymB	292.158,00	16,55	41,00	100.000
G11	pSymA	256.526,00	14,53	54,30	99.999
	pRmeGR4a	1.795,00	0,10		4,9
	pRmeGR4b	25.151,00	1,42	33,40	100.000
x74	Total	1.765.034,00			
	Chromosome	774.381,00	39,79	64,20	99.700
	pSymB	314.596,00	16,17	55,50	99.999
G12	pSymA	259.050,00	13,31	54,80	99.998
	pRmeGR4a	1.886,00	0,10		4,6
	pRmeGR4b	25.658,00	1,32	34,10	100.000
x82	Total	1.946.084,00			
	Chromosome	673.962,00	38,90	55,90	99.999
	pSymB	281.791,00	16,27	49,70	99.998
G13	pSymA	252.301,00	14,56	53,40	99.997
	pRmeGR4a	12.696,00	0,73	21,60	82,9
	pRmeGR4b	26.511,00	1,53	35,30	100.000
x73	Total	1.732.428,00			

Mean coverage are indicated as xfold for each isolate
