# Supplementary material for: The early events underlying genome evolution in a localized Sinorhizobium meliloti population
Source: BMC Genomics. 2016 Aug 5;17:556. doi: 10.1186/s12864-016-2878-9 (PMC4974801; doi:10.1186/s12864-016-2878-9)
Supplement: Additional file 4: Table S3. — sSNP string sequence. (PDF 74 kb) [file 12864_2016_2878_MOESM4_ESM.pdf]

S3 Table. sSNPs string sequence

Chromosomal sSNPs

GR4	G1	G2	G3	G4	G5	G6	G7	G8	G9	G10	G11	G12	G13	CDS Codon Number	CDS position	product	Position (original sequence)	CDS position Within Codon	Codon Change
C	C	G	C	C	C	C	C	C	C	C	C	C	C	98	294	Transcriptional regulator	92,276	3	CTC -> CTG
C	C	C	C	C	C	C	C	T	C	C	C	C	C	182	544	Flp pilus assembly protein TadB	165,554	1	CTG -> TTG
C	C	C	C	C	C	C	C	T	C	C	C	C	C	332	996	putative dehydrogenase-related protein	221,771	3	GCG -> GCA
A	A	A	A	A	A	A	C	A	A	A	A	A	A	21	63	BA14K-like protein	289,757	3	CCT -> CCG
G	G	G	G	G	G	G	G	G	G	G	G	A	G	451	1,353	exodeoxyribonuclease VII, large subunit	328,836	3	CAG -> CAA
C	C	C	C	C	C	C	C	C	C	C	T	C	C	416	1,248	The (Largely Gram-negative Bacterial) Hydrophobe/Amphiphile Efflux-1 (HAE1) Family	458,744	3	GTG -> GTA
G	G	G	G	G	G	G	G	G	G	G	G	G	A	89	267	Transcriptional regulator/sugar kinase	518,977	3	TCC -> TCT
C	C	C	C	C	C	C	C	G	C	C	C	C	C	178	534	phosphate regulon sensor kinase PhoR	540,831	3	GGC -> GGG
C	T	T	T	T	T	T	T	T	T	T	T	T	T	13	39	putative esterase of the alpha-beta hydrolase superfamily	850,884	3	GGC -> GGT
T	C	C	C	C	C	C	C	C	C	C	C	C	C	333	999	tetraacyldisaccharide 4-kinase	860,531	3	GCT -> GCC
C	C	C	C	T	C	C	C	C	C	T	C	C	C	717	2,151	PAS domain S-box/diguanylate cyclase (GGDEF) domain protein	1,024,636	3	ATC -> ATT
C	C	C	C	C	C	C	C	G	C	C	C	C	C	613	1,839	NADH:ubiquinone oxidoreductase subunit 5 (chain L)/Multisubunit Na+/H+ antiporter, MnhA subunit	1,056,869	3	GCC -> GCG
C	C	C	C	C	C	C	T	C	C	C	C	C	C	16	48	Soluble lytic murein transglycosylase and related regulatory proteins (some containing LysM/invasin domains)	1,124,870	3	GGG -> GGA
A	A	A	A	A	A	A	C	A	A	A	A	A	A	3	9	type I secretion membrane fusion protein, HlyD family	1,171,246	3	CGA -> CGC
T	C	C	C	C	C	C	C	C	C	C	C	C	T	189	567	Branched-chain amino acid ABC-type transport system, permease component	1,242,101	3	GCT -> GCC
C	C	C	C	C	C	C	C	C	C	T	C	C	C	241	723	Delta-aminolevulinic acid dehydratase	1,285,869	3	GAG -> GAA
G	G	G	G	G	G	G	T	G	G	G	G	G	G	143	429	riboflavin synthase, alpha subunit	1,293,226	3	GTG -> GTT
C	C	C	C	T	C	C	C	C	C	C	C	C	C	64	192	Protein-disulfide isomerase	1,409,691	3	CGG -> CGA
C	C	C	C	C	C	C	C	C	C	C	C	T	C	220	660	hypothetical protein	1,487,651	3	GGG -> GGA
A	C	C	A	C	C	A	C	C	C	C	C	C	C	40	120	Protein-L-isoaspartate carboxylmethyltransferase	1,599,952	3	GCA -> GCC
C	C	C	C	C	C	C	C	T	C	C	C	C	C	119	357	ABC-type spermidine/putrescine transport systems, ATPase component	1,621,428	3	AAC -> AAT
C	C	C	C	C	C	C	C	G	C	C	C	C	C	483	1,449	carbamoyl-phosphate synthase, large subunit	1,680,606	3	GCG -> GCC
C	C	C	C	C	C	C	C	C	C	C	C	C	T	41	123	Trk-type K+ transport systems, membrane component	1,738,975	3	GTC -> GTT
A	A	A	A	A	A	A	A	A	A	A	A	A	G	89	267	hypothetical protein	1,740,658	3	GTA -> GTG
G	G	G	G	G	G	G	G	G	G	G	A	G	G	35	105	ABC-type uncharacterized transport system, permease component	1,785,170	3	TTC -> TTT
C	C	C	C	C	C	C	C	C	G	C	C	C	C	163	489	hypothetical protein	1,800,697	3	ACG -> ACC
C	C	C	C	C	C	C	C	C	C	C	C	T	C	270	810	Transcriptional regulator	1,818,248	3	TAC -> TAT
G	G	G	G	G	A	G	G	G	G	G	G	G	G	153	459	Transcriptional regulator	1,828,932	3	GCG -> GCA
G	G	G	G	G	G	G	T	G	G	G	G	G	G	296	888	putative oxidoreductases (related to aryl-alcohol dehydrogenases)	2,056,580	3	ATC -> ATA
G	G	G	G	G	G	G	A	G	G	G	G	G	G	29	87	3-oxoacyl-(acyl-carrier-protein) synthase	2,077,485	3	CTC -> CTT
G	G	G	G	G	G	G	G	G	G	C	G	G	G	503	1,509	putative integral membrane protein	2,187,647	3	GCG -> GCC
G	G	G	A	G	G	G	G	G	G	G	G	G	G	114	342	ABC-type sugar transport systems, ATPase component	2,192,267	3	GGC -> GGT
C	C	T	C	C	C	C	C	C	C	C	C	C	C	131	393	Acetyltransferase, including N-acetylase of ribosomal protein	2,257,482	3	GCC -> GCT
T	T	T	T	T	T	C	T	T	C	T	T	T	T	499	1,497	TonB-dependent siderophore receptor	2,376,972	3	GCT -> GCC
G	G	G	G	G	G	G	G	G	G	G	A	G	G	193	577	ABC-type sugar transport systems, permease component	2,394,225	1	CTG -> TTG
C	C	C	C	C	C	C	C	C	T	C	C	C	C	130	390	Acyl dehydratase	2,587,145	3	ATC -> ATT
A	G	G	G	G	G	G	G	G	G	G	G	G	G	172	515	RNA polymerase sigma factor, sigma-70 family	2,603,354	2	TAA -> TGA
C	C	C	C	C	C	C	A	C	C	C	C	C	C	348	1,044	hypothetical protein	2,620,338	3	CTG -> CTT
A	A	A	A	A	G	A	A	A	A	A	A	A	A	455	1,365	Cation/multidrug efflux pump	2,691,912	3	GGA -> GGG
C	C	C	C	C	T	C	C	C	C	C	C	C	C	223	669	ABC-type dipeptide transport system, periplasmiccomponent	2,822,673	3	CTC -> CTT
C	C	C	T	C	C	C	C	C	C	C	C	C	C	57	171	hypothetical protein	2,948,779	3	CTG -> CTA
C	C	T	C	C	C	C	C	C	C	C	C	C	C	34	102	hypothetical protein	2,948,848	3	CGG -> CGA
C	C	C	C	C	C	T	C	C	C	C	C	C	C	31	93	hypothetical protein	2,949,099	3	AAG -> AAA
G	G	G	C	G	G	G	G	G	G	G	G	G	G	732	2,196	hypothetical protein	2,960,778	3	GCC -> GCG
T	T	C	T	T	T	T	T	T	T	T	T	T	T	10	30	Permeases of the drug/metabolite transporter (DMT) superfamily	2,980,370	3	GGT -> GGC
C	C	C	C	C	C	T	C	C	C	C	C	C	C	211	633	Arabinose efflux permease	3,030,279	3	GGC -> GGT
G	G	G	G	G	G	G	G	G	G	C	G	G	G	164	492	pyruvate kinase	3,046,922	3	ACC -> ACG
C	C	C	C	C	C	C	C	T	C	C	C	C	C	40	120	Response regulator containing CheY-like receiver, AAA-type ATPase, and DNA-binding domains	3,092,319	3	GAC -> GAT
C	C	C	C	C	C	C	C	C	C	C	A	C	C	154	462	Transcriptional regulator	3,219,241	3	CCG -> CCT
G	G	G	G	G	G	G	G	A	G	G	G	G	G	115	345	succinyl-CoA synthetase, beta subunit	3,361,218	3	GAC -> GAT
C	G	G	C	G	G	C	G	G	G	G	G	G	G	340	1,02	C-terminal peptidase (prc)	3,412,118	3	ACC -> ACG
G	G	G	G	G	A	G	G	G	G	G	G	G	G	143	429	thiamine ABC transporter, permease protein	3,500,599	3	CCG -> CCA
G	G	G	G	G	G	G	G	G	A	G	G	G	G	47	141	hypothetical protein	3,545,779	3	TCG -> TCA
C	C	C	C	C	C	C	C	C	C	C	T	C	C	438	1,314	FAD/FMN-containing dehydrogenase	3,548,363	3	CGC -> CGT
G	G	G	G	G	G	C	G	G	G	G	G	G	G	167	501	Choline dehydrogenase-related flavoprotein	3,559,782	3	GCC -> GCG
C	C	C	C	C	C	C	C	C	C	G	C	C	C	177	531	DNA polymerase III, delta subunit	3,611,109	3	GGC -> GGG

pSymB sSNPs

GR4	G1	G2	G3	G4	G5	G6	G7	G8	G9	G10	G11	G12	G13	CDS Codon Number	CDS Position	product	Min (original sequence)	CDS Position Within Codon	Codon Change
G	G	G	G	T	G	G	G	G	G	T	T	G	G	368	1,104	putative dehydrogenase-related protein	39,808	3	CCG -> CCT
C	C	C	C	C	C	C	C	C	C	C	C	C	G	74	222	ABC-type sugar transport system, permease component	88,537	3	GCC -> GCG
G	G	G	G	G	G	G	T	G	G	G	G	G	G	182	546	Ribose/xylose/arabinose/galactoside ABC-type transport systems, permease component	100,115	3	GGG -> GGT
C	C	T	C	C	C	C	C	C	C	C	C	T	C	61	183	thiamine biosynthesis protein ThiS	104,199	3	CCG -> CCA
C	C	C	C	T	C	C	C	C	C	T	T	C	C	273	819	Transcriptional regulator	223,297	3	TCC -> TCT
G	G	G	G	G	G	G	G	G	C	G	G	G	G	6	18	Hydroxypyruvate isomerase	271,987	3	GCC -> GCG
C	C	C	C	C	C	C	G	C	C	C	C	C	C	127	381	hypothetical protein	305,017	3	ACC -> ACG
G	G	G	G	G	G	G	G	G	C	G	G	G	G	306	918	cytochrome o ubiquinol oxidase, subunit I	331,199	3	GGC -> GGG
C	C	C	G	C	C	C	C	C	C	C	C	C	C	207	621	hypothetical protein	358,743	3	CGC -> CGG
G	G	G	G	G	G	G	G	G	G	G	G	A	G	148	444	4-aminobutyrate aminotransferase and related aminotransferase	527,24	3	GGG -> GGA
G	G	G	G	G	G	G	G	G	G	C	G	G	G	390	1,17	PAS domain S-box	574,83	3	GGC -> GGG
T	T	T	T	C	T	T	T	T	T	T	T	T	T	139	417	ABC-type sugar transport systems, ATPase component	586,363	3	CGT -> CGC
G	G	G	G	G	G	G	G	G	G	G	G	A	G	1,601	4,803	hypothetical protein	650,927	3	GGC -> GGT
G	G	G	G	A	G	G	G	G	G	G	G	G	G	164	492	Glycine/D-amino acid oxidases (deaminating)	682,064	3	GAC -> GAT
C	C	C	C	C	C	G	C	C	C	C	C	C	C	172	516	ABC-type dipeptide/oligopeptide/nickel transportsystems, permease component	790,565	3	CGG -> CGC
G	G	G	G	A	G	G	G	G	G	G	G	G	G	230	690	ABC-type proline/glycine betaine transport systems, ATPase component	846,602	3	CCC -> CCT
A	A	A	A	A	G	A	A	A	A	A	A	A	A	138	414	NAD-dependent aldehyde dehydrogenase	855,118	3	CAT -> CAC
C	C	C	C	T	C	C	C	C	C	C	C	C	C	191	573	amidohydrolase	883,953	3	CGC -> CGT
G	G	G	G	G	G	G	G	G	G	G	C	G	G	198	594	nucleotide sugar dehydrogenase	1,054,847	3	CTG -> CTC
G	G	G	G	G	A	G	G	G	G	G	G	G	G	38	114	Ribose/xylose/arabinose/galactoside ABC-type transport systems, permease component	1,115,162	3	CCG -> CCA
A	A	A	A	A	A	A	A	A	A	A	C	A	A	159	477	Capsule polysaccharide export protein	1,136,420	3	CGA -> CGC
G	G	G	G	G	G	G	G	G	A	G	G	G	G	186	558	GSCFA family	1,141,488	3	CTC -> CTT
C	C	C	C	C	C	C	C	C	C	C	C	G	C	60	180	ribose 5-phosphate isomerase B	1,362,935	3	CGC -> CGG
G	G	G	G	G	G	G	G	G	G	G	G	A	G	58	174	Protein of unknown function (DUF3365)	1,382,509	3	ATC -> ATT
G	G	G	G	A	G	G	G	G	G	A	G	G	G	314	942	TRAP transporter, DctM subunit	1,441,115	3	ATC -> ATT
G	G	G	G	G	G	G	G	A	G	G	G	G	G	306	918	NAD-dependent aldehyde dehydrogenase	1,477,753	3	GCC -> GCT
C	C	C	C	C	C	T	C	C	C	C	C	C	C	63	189	Glycosyltransferase	1,496,925	3	TCC -> TCT
G	G	G	C	G	G	G	G	G	G	G	G	G	G	199	597	bifunctional S-(hydroxymethyl)glutathione dehydrogenase/class III alcohol dehydrogenase	1,556,530	3	GGC -> GGG
A	A	A	G	A	A	A	A	A	A	A	A	A	A	194	582	bifunctional S-(hydroxymethyl)glutathione dehydrogenase/class III alcohol dehydrogenase	1,556,545	3	GGT -> GGC
A	A	A	A	A	A	A	A	A	A	A	G	A	A	681	2,043	Aerobic-type carbon monoxide dehydrogenase, large subunit CoxL/CutL-like protein	1,605,960	3	CTT -> CTC
G	G	G	G	G	G	G	G	G	G	G	C	G	G	10	30	ABC-type uncharacterized transport system, permease component	1,613,155	3	CTG -> CTC
T	C	C	C	C	C	T	C	C	C	C	C	C	C	141	423	Erythromycin esterase-like protein	1,655,309	3	GAA -> GAG
G	G	G	G	G	G	G	G	T	G	G	G	G	G	23	69	hypothetical protein	1,676,833	3	ATC -> ATA
G	G	G	G	A	G	G	G	G	G	A	G	G	G	212	636	ABC-type Fe3+-siderophore transport system, permease component	1,688,119	3	GCC -> GCT

pSymA sSNPs

GR4	G1	G2	G3	G4	G5	G6	G7	G8	G9	G10	G11	G12	G13	CDS Codon Number	CDS Position	product	Min (original sequence)	CDS Position Within Codon	Codon Change
G	A	A	A	A	A	A	A	A	A	A	A	A	A	26	78	Short-chain alcohol dehydrogenase of unknown specificity	32,129	3	GGC -> GGT
C	C	C	C	T	C	C	C	C	C	C	C	C	C	32	96	putative redox protein, regulator of disulfide bond formation	159,713	3	GGG -> GGA
C	C	C	A	C	C	C	C	C	C	C	C	C	C	225	675	GTPase subunit of restriction endonuclease	250,248	3	CGG -> CGT
C	C	T	C	C	C	C	C	C	C	C	C	C	C	390	1,17	ABC-type dipeptide transport system, periplasmiccomponent	373,935	3	GCG -> GCA
T	T	T	T	C	T	T	T	T	T	C	T	T	T	190	570	hypothetical protein	490,122	3	CAT -> CAC
G	G	G	G	A	G	G	G	G	G	G	G	G	G	160	480	Flp pilus assembly protein, ATPase CpaF	528,638	3	GAG -> GAA
G	G	G	G	G	G	G	G	C	G	G	G	G	G	228	684	Collagenase and related protease	698,557	3	CTC -> CTG
T	T	T	T	T	T	T	T	T	T	T	A	T	T	251	753	Ornithine carbamoyltransferase	864,279	3	CCT -> CCA
C	C	C	C	C	C	C	C	C	C	C	C	T	C	429	1,287	Ti-type conjugative transfer system protein TraG	953,37	3	ATC -> ATT
C	C	T	C	C	C	C	C	C	C	C	C	T	C	913	2,739	DNA-directed DNA polymerase III (polc)	967,124	3	CTG -> CTA
C	C	C	C	T	C	C	C	C	C	T	T	C	C	444	1,332	DNA-directed DNA polymerase III (polc)	968,531	3	CGG -> CGA
C	C	C	C	T	C	C	C	C	C	C	C	C	C	251	753	2-keto-3-deoxy-galactonokinase	1,085,437	3	TTC -> TTT
G	T	G	G	G	G	G	G	G	G	G	G	G	G	63	189	hypothetical protein	1,087,226	3	GCC -> GCA
G	G	G	G	G	A	G	G	G	G	G	G	G	G	344	1,032	Multidrug resistance efflux pump	1,108,260	3	TTC -> TTT
A	G	G	G	G	A	G	G	G	G	G	G	G	G	213	639	Zn-dependent alcohol dehydrogenase	1,142,894	3	GCT -> GCC
C	C	C	C	C	C	C	C	C	C	G	C	C	C	39	117	hypothetical protein	1,185,966	3	GTC -> GTG
G	G	G	G	G	A	G	G	G	G	G	G	G	G	143	429	Transcriptional regulator containing an amidase domain and an AraC-type DNA-binding HTH domain	1,229,677	3	GTG -> GTA
C	C	C	C	C	C	C	C	T	C	C	C	C	C	145	435	Ribose/xylose/arabinose/galactoside ABC-type transport systems, permease component	1,298,013	3	GTC -> GTT
T	T	T	T	T	T	G	T	T	T	T	T	T	T	217	651	amine acid ABC transporter, permease protein, 3-TM region, His/Glu/Gln/Arg/opine family	1,374,862	3	GCA -> GCC
