# Supplementary material for: The early events underlying genome evolution in a localized Sinorhizobium meliloti population
Source: BMC Genomics. 2016 Aug 5;17:556. doi: 10.1186/s12864-016-2878-9 (PMC4974801; doi:10.1186/s12864-016-2878-9)
Supplement: Additional file 5: Table S4. — nsSNP string sequence. (PDF 100 kb) [file 12864_2016_2878_MOESM5_ESM.pdf]

S4 Table. nsSNPs string sequence

Chromosome nsSNPs

GR4	G1	G2	G3	G4	G5	G6	G7	G8	G9	G10	G11	G12	G13	CDS Codon Number	CDS Position	product	Position (original sequence)	Amino Acid Change	CDS Position Within Codon	Codon Change
C	C	C	C	C	C	C	C	C	C	C	C	T	C	148	442	putative metal-binding integral membrane protein	209,63	A -> T	1	GCG -> ACG
C	C	C	C	C	C	C	C	G	C	C	C	C	C	256	767	N-acetylglucosamine-6-phosphate deacetylase	230,766	P -> R	2	CCG -> CGG
C	C	C	C	C	C	C	C	C	C	C	T	C	C	439	1,316	DNA polymerase III, subunit gamma and tau	257,348	P -> L	2	CCA -> CTA
T	T	T	T	T	C	T	T	T	T	T	T	T	T	237	710	Ketosteroid isomerase-like protein	399,584	V -> A	2	GTC -> GCC
A	A	A	A	A	A	A	A	A	A	A	A	A	T	423	1,268	Methyl-accepting chemotaxis protein	447,247	L -> Q	2	CTG -> CAG
C	C	C	C	C	G	C	C	C	C	C	C	C	C	354	1,062	Capsule polysaccharide export protein	610,579	Q -> H	3	CAG -> CAC
A	G	G	A	G	G	A	G	G	G	G	G	G	G	191	573	flagellar basal-body rod protein FlgF	692,094	I -> M	3	ATA -> ATG
A	A	A	A	A	A	A	C	A	A	A	A	A	A	319	955	FAD/FMN-containing dehydrogenase	822,359	M -> L	1	ATG -> CTG
T	T	T	T	T	T	T	T	G	T	T	T	T	T	200	598	hypothetical protein	870,048	T -> P	1	ACC -> CCC
C	T	T	C	T	T	C	T	T	T	T	T	T	T	163	487	hypothetical protein	870,159	A -> T	1	GCG -> ACG
G	G	G	G	G	G	G	G	G	G	G	A	G	G	248	742	putative NAD(P)H quinone oxidoreductase, PIG3 family	1,017,176	G -> S	1	GGC -> AGC
A	A	A	A	A	A	A	C	A	A	A	A	A	A	16	47	putative lactoylglutathione lyase	1,035,577	V -> G	2	GTC -> GGC
C	C	C	C	C	C	T	C	C	C	C	C	C	C	434	1,301	Dehydrogenases (flavoproteins)	1,101,589	S -> L	2	TCG -> TTG
C	C	C	C	C	C	C	C	C	T	C	C	C	C	45	134	Cu/Zn superoxide dismutase	1,168,167	T -> M	2	ACG -> ATG
C	G	G	G	G	G	G	G	G	G	G	G	G	G	149	445	Molybdopterin converting factor, large subunit	1,242,587	G -> R	1	GGC -> CGC
T	T	T	T	T	T	T	C	T	T	T	T	T	T	359	1,075	bifunctional exopolyphosphatase	1,265,228	Y -> H	1	TAC -> CAC
C	C	C	C	A	C	C	C	C	C	C	C	C	C	236	706	hypothetical protein	1,279,363	V -> F	1	GTC -> TTC
A	A	A	A	A	G	A	A	A	A	A	A	A	A	8	22	hypothetical protein	1,293,834	S -> P	1	TCC -> CCC
A	A	A	A	T	A	A	A	A	A	T	A	A	A	44	131	3-oxoacyl-(acyl-carrier-protein) synthase III	1,303,381	Y -> F	2	TAT -> TTT
A	A	C	A	A	A	A	A	A	A	A	A	A	A	218	653	Glycosyltransferase	1,308,917	L -> R	2	CTC -> CGC
C	A	A	C	A	A	C	A	A	A	A	A	A	A	20	58	putative CoA-binding protein	1,326,918	P -> T	1	CCC -> ACC
C	C	C	C	C	T	C	C	C	C	C	C	C	C	294	880	Cytochrome P450	1,329,846	E -> K	1	GAG -> AAG
C	C	A	C	C	C	C	C	C	C	C	C	A	C	12	34	protein CrcB	1,472,491	A -> S	1	GCC -> TCC
C	C	C	C	C	C	C	C	C	C	C	C	T	C	6	16	Putative protein-S-isoprenylcysteine methyltransferase	1,487,269	A -> T	1	GCC -> ACC
C	C	C	C	C	C	C	C	G	C	C	C	C	C	213	637	hypothetical protein	1,487,674	A -> P	1	GCC -> CCC
A	A	A	A	A	A	A	A	A	A	T	A	A	A	1	2	hypothetical protein	1,512,108	M -> K	2	ATG -> AAG
C	C	C	C	C	C	C	G	C	C	C	C	C	C	360	1,078	NADH:flavin oxidoreductase, Old Yellow Enzyme family	1,666,779	A -> P	1	GCC -> CCC
A	G	G	G	G	G	G	G	G	G	G	G	G	G	8	22	hypothetical protein	1,696,288	F -> L	1	TTT -> CTT
C	C	C	C	C	C	C	C	C	C	C	G	C	C	164	490	hypothetical protein	1,719,243	L -> V	1	CTC -> GTC
C	T	T	C	T	T	C	T	T	T	T	T	T	T	499	1,496	5-nucleotidase/2,3-cyclic phosphodiesterase and related esterase	1,736,263	P -> L	2	CCG -> CTG
A	G	A	A	A	A	A	A	A	A	A	A	A	A	561	1,682	ABC-type oligopeptide transport system, periplasmic component	1,752,626	N -> S	2	AAC -> AGC
C	C	C	C	C	C	C	C	C	C	C	C	C	G	185	555	Molybdopterin biosynthesis enzyme	1,778,354	D -> E	3	GAC -> GAG
G	G	G	G	G	G	G	G	G	G	A	G	G	G	18	53	MoxR-like ATPase	1,873,700	A -> V	2	GCG -> GTG
G	G	G	A	G	G	G	G	G	G	G	G	G	G	130	390	phosphoribosylformylglycinamidine synthase II	1,896,184	M -> I	3	ATG -> ATA
T	T	T	C	T	T	T	T	T	T	T	T	T	T	140	419	hypothetical protein	1,916,986	V -> A	2	GTG -> GCG
C	T	C	C	C	C	C	C	C	C	C	C	C	C	113	337	putative flavoprotein	1,958,645	G -> S	1	GGC -> AGC
C	C	C	C	C	C	C	C	C	C	C	C	C	T	90	268	Dehydrogenases with different specificities (related to short-chain alcohol dehydrogenases)	1,978,051	V -> I	1	GTC -> ATC
T	T	T	T	T	G	T	T	T	T	T	T	T	T	390	1,169	bifunctional cobyrinic acid a,c-diamide synthase	2,081,983	E -> A	2	GAG -> GCG
C	C	C	C	C	A	C	C	C	C	C	C	C	C	41	123	hypothetical protein	2,100,940	S -> R	3	AGC -> AGA
C	C	C	C	G	C	C	C	C	C	G	G	C	C	76	226	putative addiction module antidote protein, CC2985 family	2,109,946	R -> G	1	CGT -> GGT
G	G	G	G	G	G	G	G	G	G	G	A	G	G	72	215	HAD-superfamily hydrolase, subfamily IIB	2,191,195	T -> M	2	ACG -> ATG
G	G	C	G	G	G	G	G	G	G	G	G	C	G	43	127	diguanylate cyclase (GGDEF) domain protein	2,286,294	G -> R	1	GGC -> CGC
A	A	A	A	A	C	A	A	A	A	A	A	A	A	384	1,151	lytic murein transglycosylase	2,345,472	K -> T	2	AAG -> ACG
T	T	C	T	T	T	T	T	T	T	T	T	T	T	1,061	3,182	RHS repeat-associated core domain protein	2,496,889	Q -> R	2	CAA -> CGA
C	C	C	C	C	C	C	C	C	C	C	C	C	G	183	547	L-threonine 3-dehydrogenase	2,544,991	G -> R	1	GGG -> CGG
T	T	T	T	T	T	T	T	T	T	T	T	C	T	87	260	L-threonine 3-dehydrogenase	2,545,278	E -> G	2	GAG -> GGG
A	A	A	A	A	A	A	A	A	A	A	C	A	A	211	632	Transcriptional activator of acetoin/glycerol metabolism	2,580,136	V -> G	2	GTC -> GGC
G	G	G	G	G	G	G	G	G	T	G	G	G	G	816	2,446	Outer membrane receptor for Fe3+-dicitrate	2,610,749	V -> F	1	GTC -> TTC
T	T	T	T	A	T	T	T	T	T	T	T	T	T	568	1,703	TonB-dependent heme/hemoglobin receptor family protein/TonB-dependent hemoglobin/transferrin/lactoferrin receptor family protein	2,615,415	N -> I	2	AAT -> ATT
T	G	G	G	G	G	G	G	G	G	G	G	G	G	27	80	putative transcriptional regulator	2,633,947	L -> R	2	CTC -> CGC
G	G	G	G	G	A	G	G	G	G	G	G	G	G	89	266	Uncharacterized protein conserved in bacteria (DUF2325)	2,651,781	P -> L	2	CCG -> CTG
G	A	A	A	A	A	A	A	A	A	A	A	A	A	7	19	ABC-type hemin transport system, ATPase component	2,657,237	V -> I	1	GTT -> ATT
A	A	A	A	A	A	A	A	A	T	A	A	A	A	247	740	urease, alpha subunit	2,699,283	I -> N	2	ATC -> AAC
T	T	T	T	T	T	T	T	T	T	T	T	T	G	70	208	ABC-type branched-chain amino acid transport systems, ATPase component	2,708,954	I -> L	1	ATC -> CTC
T	C	C	C	C	C	T	C	C	C	C	C	C	C	12	35	ABC-type dipeptide/oligopeptide/nickel transportsystems, permease component	2,769,803	D -> G	2	GAC -> GGC
G	G	G	G	G	G	G	G	G	C	G	G	G	G	145	434	Dehydrogenases with different specificities (related to short-chain alcohol dehydrogenases)	2,780,169	G -> A	2	GGG -> GCG
G	G	G	G	G	G	G	G	G	A	G	G	G	G	218	652	Glycerol dehydrogenase-related enzyme	2,781,217	A -> T	1	GCG -> ACG
G	G	G	G	G	G	G	G	G	T	G	G	G	G	28	82	Short-chain alcohol dehydrogenase of unknown specificity	2,781,759	A -> S	1	GCC -> TCC
G	G	G	G	G	G	G	G	G	G	G	G	A	G	327	979	ABC-type branched-chain amino acid transport systems, periplasmic component	2,814,717	A -> T	1	GCC -> ACC
C	C	C	C	C	C	C	C	C	C	C	C	C	T	262	784	ABC-type dipeptide/oligopeptide/nickel transportsystems, permease component	2,828,883	P -> S	1	CCG -> TCG
T	T	T	T	T	G	T	T	T	T	T	T	T	T	359	1,076	Membrane proteins related to metalloendopeptidase	2,847,767	L -> R	2	CTC -> CGC
T	T	T	T	T	T	T	C	T	T	T	T	T	T	194	581	Cell division protein	2,899,455	V -> A	2	GTC -> GCC
G	G	G	G	G	G	G	G	G	A	G	G	G	G	18	52	hypothetical protein	2,923,118	G -> S	1	GGC -> AGC
A	A	A	G	A	A	A	A	A	A	A	A	A	A	76	227	Zn-dependent alcohol dehydrogenase	2,930,507	V -> A	2	GTC -> GCC
A	A	A	A	C	A	A	A	A	A	A	A	A	A	90	269	hypothetical protein	2,953,687	L -> R	2	CTT -> CGT
G	G	G	G	A	G	G	G	G	G	G	G	G	G	126	376	luciferase family oxidoreductase, group 1	3,007,978	A -> T	1	GCG -> ACG
C	C	C	C	C	C	C	C	C	C	C	C	A	C	40	120	glycogen debranching enzyme GlgX	3,119,021	N -> K	3	AAC -> AAA
G	G	G	G	G	C	G	C	G	G	G	G	G	G	155	463	hypothetical protein	3,157,427	D -> H	1	GAC -> CAC
G	G	G	G	G	G	G	G	G	G	A	G	G	G	180	538	Inosine-uridine nucleoside N-ribohydrolase	3,181,864	V -> M	1	GTG -> ATG
C	C	C	C	C	C	C	C	C	C	C	C	C	T	245	733	bifunctional precorrin-6y C5,15-methyltransferase (decarboxylating), CbiE subunit/precorrin-6Y C5,15-methyltransferase (decarboxylating), CbiT subunit	3,197,243	G -> S	1	GGC -> AGC
G	G	G	G	G	G	G	G	C	G	G	G	G	G	5	15	Transcriptional regulator	3,219,688	D -> E	3	GAC -> GAG
A	A	A	A	A	A	A	A	A	A	A	A	T	A	381	1,141	ABC-type dipeptide transport system, periplasmiccomponent	3,249,929	Y -> N	1	TAC -> AAC
T	G	G	G	G	G	G	G	G	G	G	G	G	G	1,063	3,189	hypothetical protein	3,296,058	N -> K	3	AAT -> AAG
G	G	G	G	G	G	G	A	G	G	G	G	G	G	150	448	Aminoglycoside phosphotransferase	3,299,561	V -> I	1	GTT -> ATT
G	G	G	G	G	G	G	G	A	G	G	G	G	G	25	74	hypothetical protein	3,353,831	T -> I	2	ACC -> ATC
A	A	A	A	A	A	A	A	C	A	A	A	A	A	47	139	hypothetical protein	3,401,493	S -> A	1	TCG -> GCG
C	A	C	C	C	C	C	C	C	C	C	C	C	C	184	551	nicotinate (nicotinamide) nucleotide adenylyltransferase	3,408,407	Stop	2	TCG ->TAG
T	T	G	T	T	T	T	T	T	T	T	T	T	T	956	2,866	DNA-directed DNA polymerase III (polc)	3,416,139	M -> L	1	ATG -> CTG
G	G	G	G	G	G	A	G	G	G	G	G	G	G	124	371	hypothetical protein	3,462,427	A -> V	2	GCA -> GTA
A	A	A	A	A	A	A	A	A	A	A	A	A	C	421	1,263	NAD-dependent aldehyde dehydrogenase	3,565,002	D -> E	3	GAT -> GAG

pSymB nsSNPs

GR4	G1	G2	G3	G4	G5	G6	G7	G8	G9	G10	G11	G12	G13	CDS Codon Number	CDS Position	product	Position (original sequence)	Amino Acid Change	CDS Position Within Codon	Codon Change
G	G	G	G	A	G	G	G	G	G	A	G	G	G	114	340	ABC-type dipeptide/oligopeptide/nickel transportsystems, permease component	67,425	D -> N	1	GAC -> AAC
A	G	A	A	A	A	A	A	A	A	A	A	A	A	45	134	thiamine-phosphate pyrophosphorylase	103,278	V -> A	2	GTC -> GCC
G	G	G	G	G	A	G	G	G	G	G	G	G	G	6	16	hypothetical protein	107,638	A -> T	1	GCA -> ACA
C	C	C	C	C	T	C	C	C	C	C	C	C	C	367	1,099	urea ABC transporter, permease protein UrtC	131,058	H -> Y	1	CAC -> TAC
G	G	G	G	G	G	G	G	G	G	G	G	G	A	67	199	Pyridoxal phosphate biosynthesis protein	177,694	D -> N	1	GAT -> AAT
A	A	A	A	A	T	A	A	A	A	A	A	A	A	179	536	Membrane protein involved in colicin uptake	263,69	K -> M	2	AAG -> ATG
T	G	T	T	T	T	T	T	T	T	T	T	T	T	71	211	TRAP-type C4-dicarboxylate transport system, small permease component	373,687	F -> V	1	TTT -> GTT
A	A	A	A	A	A	A	A	A	C	A	A	A	A	489	1,466	ABC-type sugar transport system, ATPase component	427,945	E -> A	2	GAG -> GCG
T	T	T	T	T	T	T	T	G	T	T	T	T	T	257	770	hypothetical protein	437,069	Y -> S	2	TAC -> TCC
C	C	C	C	C	C	C	C	C	C	C	A	C	C	33	99	RND family efflux transporter, MFP subunit	440,238	F -> L	3	TTC -> TTA
T	T	T	T	T	T	T	T	T	T	T	T	G	T	204	610	xanthine dehydrogenase, small subunit	486,137	S -> R	1	AGC -> CGC
T	C	T	T	T	T	T	T	T	T	T	T	T	T	262	785	ABC-type amino acid transport system, permease component	525,224	V -> A	2	GTG -> GCG
C	C	C	C	C	C	C	C	T	C	C	C	C	C	134	401	phosphonoacetate hydrolase	669,373	R -> Q	2	CGG -> CAG
C	C	C	C	C	G	C	C	C	C	C	C	C	C	171	511	Na+/phosphate symporter	672,259	A -> P	1	GCG -> CCG
T	T	T	T	T	T	C	T	T	T	T	T	T	T	231	691	ABC-type sugar transport system, periplasmic component	693,997	S -> P	1	TCG -> CCG
C	C	C	C	C	G	C	C	C	C	C	C	C	C	115	344	hypothetical protein	707,439	P -> R	2	CCC -> CGC
G	G	G	G	G	G	G	A	G	G	G	G	G	G	475	1,423	Glycosyltransferase	748,793	L -> F	1	CTC -> TTC
G	G	G	G	G	G	A	G	G	G	G	G	G	G	170	508	ABC-type dipeptide transport system, periplasmiccomponent	768,557	V -> I	1	GTC -> ATC
A	A	A	A	A	A	A	A	G	A	A	A	A	A	119	356	Lactoylglutathione lyase and related lyase	783,43	F -> S	2	TTT -> TCT
A	A	A	G	A	A	A	A	A	A	A	A	A	A	26	76	phosphonate ABC transporter, permease protein PhnE	815,724	N -> D	1	AAT -> GAT
A	G	G	A	G	G	A	G	G	G	G	G	G	G	99	297	phosphonate metabolism protein/1,5-bisphosphokinase (PRPP-forming) PhnN	820,613	I -> M	3	ATA -> ATG
T	T	A	T	T	T	T	T	T	T	T	T	T	T	58	173	Putative translation initiation inhibitor, yjgF family	836,642	N -> I	2	AAT -> ATT
T	T	T	T	T	T	T	T	T	T	T	T	G	T	269	806	Periplasmic glycine betaine/choline-binding (lipo)protein of an ABC-type transport system (osmoprotectant binding protein)	848,677	E -> A	2	GAA -> GCA
G	G	G	G	G	G	G	G	G	G	G	G	G	C	486	1,457	NAD-dependent aldehyde dehydrogenase	854,075	P -> R	2	CCG -> CGG
C	G	G	G	G	G	G	G	G	G	G	G	G	G	184	552	Polysaccharide deacetylase	913,819	E -> D	3	GAG -> GAC
C	C	C	C	C	C	A	C	C	C	C	C	C	C	97	289	putative pyridoxal phosphate-dependent enzyme apparently involved in regulation of cell wall biogenesis	921,29	A -> S	1	GCC -> TCC
C	C	C	A	C	C	C	C	C	C	C	C	C	C	300	900	Hemolysin-type calcium-binding repeat (2 copies)	944,111	F -> L	3	TTC -> TTA
G	G	G	G	G	G	G	G	G	T	G	G	G	G	4	10	hypothetical protein	972,649	H -> N	1	CAC -> AAC
G	G	G	G	G	G	G	C	G	G	G	G	G	G	157	471	Acetyl/propionyl-CoA carboxylase, alpha subunit	976,279	I -> M	3	ATC -> ATG
G	G	G	G	G	A	G	G	G	G	G	G	G	G	23	67	putative metal-dependent hydrolase of the TIM-barrel fold protein	995,619	V -> I	1	GTT -> ATT
C	C	A	C	C	C	C	C	C	C	C	C	C	C	212	636	ABC-type amino acid transport/signal transduction systems, periplasmic component/domain protein	999,217	K -> N	3	AAG -> AAT
T	G	G	T	T	G	T	G	G	G	T	G	T	G	1,054	3,16	Ti-type conjugative transfer relaxase TraA	1,010,390	N -> H	1	AAT -> CAT
T	T	T	T	T	T	T	T	T	T	T	T	C	T	375	1,123	Glycosyltransferase	1,023,530	K -> E	1	AAG -> GAG
T	T	T	T	T	T	C	T	T	C	T	T	T	T	21	62	hypothetical protein	1,078,111	L -> P	2	CTG -> CCG
T	C	C	C	C	C	T	C	C	C	C	C	C	C	26	77	hypothetical protein	1,078,126	V -> A	2	GTA -> GCA
T	T	G	T	T	T	T	T	T	T	T	T	T	T	208	622	Ribose/xylose/arabinose/galactoside ABC-type transport systems, permease component	1,114,599	F -> V	1	TTT -> GTT
C	C	C	C	C	C	C	C	C	C	C	C	G	C	313	937	hypothetical protein	1,121,573	G -> R	1	GGA -> CGA
T	T	T	T	T	T	T	T	G	T	T	T	T	T	151	452	Methylase involved in ubiquinone/menaquinone biosynthesis	1,147,542	E -> A	2	GAG -> GCG
C	C	C	C	C	C	C	C	C	C	C	T	C	C	279	835	putative acyltransferase	1,165,490	G -> R	1	GGG -> AGG
A	T	T	A	T	T	A	T	T	T	T	T	T	T	31	92	Copper chaperone	1,177,123	D -> V	2	GAT -> GTT
G	C	C	C	C	C	G	C	C	C	C	C	C	C	55	163	Protein of unknown function (DUF1515)	1,217,777	L -> V	1	CTC -> GTC
G	G	G	G	G	G	G	G	G	G	G	G	G	C	157	469	Dehydrogenases with different specificities (related to short-chain alcohol dehydrogenases)	1,228,402	P -> A	1	CCA -> GCA
C	C	C	C	C	C	C	C	C	C	C	C	C	T	77	230	hypothetical protein	1,277,682	S -> F	2	TCC -> TTC
G	G	A	G	G	G	G	G	G	G	G	G	G	G	108	323	Protein of unknown function (DUF1236)	1,288,431	P -> L	2	CCA -> CTA
A	A	A	A	A	G	A	A	A	A	A	A	A	A	259	776	diguanylate cyclase (GGDEF) domain protein	1,345,325	D -> G	2	GAC -> GGC
C	C	C	C	C	C	T	C	C	C	C	C	C	C	83	248	hypothetical protein	1,349,665	T -> I	2	ACT -> ATT
A	A	A	A	C	A	A	A	A	A	C	A	A	A	10	29	NAD-dependent aldehyde dehydrogenase	1,356,633	Y -> S	2	TAT -> TCT
T	T	T	T	T	T	T	T	T	T	T	T	T	C	349	1,046	TRAP transporter, 4TM/12TM fusion protein	1,447,336	I -> T	2	ATC -> ACC
A	T	T	A	T	T	A	T	T	T	T	T	T	T	191	572	Threonine dehydrogenase and related Zn-dependentdehydrogenase	1,493,611	L -> Q	2	CTA -> CAA
T	T	T	T	T	T	T	T	T	T	G	T	T	T	80	238	PQQ-dependent catabolism-associated CXXCW motif protein	1,551,087	T -> P	1	ACG -> CCG
T	T	T	T	T	T	T	T	T	T	T	T	T	C	12	35	ABC-type uncharacterized transport systems, ATPase component	1,615,208	I -> T	2	ATC -> ACC
G	G	G	G	G	C	G	G	G	G	G	G	G	G	325	973	ABC-type uncharacterized transport systems, ATPase component	1,616,146	G -> R	1	GGC -> CGC
C	C	C	C	C	C	C	C	T	C	C	C	C	C	455	1,363	Thiamine pyrophosphate-requiring enzymes	1,647,718	V -> I	1	GTC -> ATC

pSymA nsSNPs

GR4	G1	G2	G3	G4	G5	G6	G7	G8	G9	G10	G11	G12	G13	CDS Codon Number	CDS Position	product	Position (original sequence)	Amino Acid Change	CDS Position Within Codon	Codon Change
A	C	C	A	C	C	A	C	C	C	C	C	C	C	508	1,523	TonB-dependent siderophore receptor	35,224	I -> S	2	ATC -> AGC
T	T	G	T	T	T	T	T	T	T	T	T	G	T	74	220	putative integral membrane protein	157,299	F -> V	1	TTT -> GTT
C	C	C	C	C	C	C	C	C	C	C	T	C	C	40	118	Response regulator	169,011	E -> K	1	GAA -> AAA
A	A	A	G	A	A	A	A	A	A	A	A	A	A	260	779	Spermidine/putrescine-binding periplasmic protein	185,21	K -> R	2	AAG -> AGG
G	G	G	G	T	G	G	G	G	G	G	G	G	G	253	757	Multidrug resistance efflux pump	235,66	V -> F	1	GTC -> TTC
G	G	G	G	G	G	G	G	C	G	G	G	G	G	9	26	putative conserved protein, contains double-stranded beta-helix domain	271,27	T -> S	2	ACC -> AGC
G	G	A	G	G	G	G	G	G	G	G	G	G	G	146	437	Transcriptional regulator	276,474	R -> Q	2	CGG -> CAG
T	T	T	T	T	C	T	T	T	T	T	T	T	T	116	347	Acetyltransferase (GNAT) family	280,228	N -> S	2	AAC -> AGC
A	A	A	A	A	A	A	A	C	A	A	A	A	A	115	344	Transcriptional regulator	302,251	I -> S	2	ATC -> AGC
A	A	A	A	A	A	A	A	A	A	A	A	A	G	142	425	putative ornithine cyclodeaminase	366,994	L -> P	2	CTT -> CCT
T	T	T	T	C	T	T	T	T	T	T	T	T	T	199	595	Transcriptional regulator	576,573	T -> A	1	ACA -> GCA
G	G	G	G	G	G	G	G	G	G	G	A	G	G	492	1,474	ABC-type dipeptide transport system, periplasmiccomponent	607,062	E -> K	1	GAG -> AAG
A	A	A	A	C	A	A	A	A	A	A	A	A	A	207	619	ABC-type sugar transport system, periplasmic component	613,958	K -> Q	1	AAG -> CAG
G	G	G	G	G	G	G	T	G	G	G	G	G	G	35	103	Dehydrogenases with different specificities (related to short-chain alcohol dehydrogenases)	630,249	A -> S	1	GCA -> TCA
C	C	C	C	C	T	C	C	C	C	C	C	C	C	233	697	Sugar phosphate isomerase/epimerase	662,267	G -> S	1	GGC -> AGC
G	G	G	G	G	G	G	G	G	G	G	G	G	C	349	1,045	hypothetical protein	675,032	D -> H	1	GAT -> CAT
C	C	C	C	C	G	C	C	C	C	C	C	C	C	789	2,365	type IV secretion/conjugal transfer ATPase, VirB4 family	687,057	P -> A	1	CCC -> GCC
G	A	A	A	A	A	A	A	A	A	A	A	A	A	356	1,067	Nitric oxide reductase large subunit	707,254	C -> Y	2	TGT -> TAT
C	C	C	C	C	C	C	C	C	C	C	G	C	C	30	89	periplasmic nitrate reductase, large subunit	724,608	A -> G	2	GCG -> GGG
C	C	C	C	C	C	C	C	C	C	C	C	C	G	507	1,521	cytochrome c oxidase accessory protein FixG	738,61	H -> Q	3	CAC -> CAG
T	T	T	T	T	T	T	T	C	T	T	T	T	T	19	55	Aerobic-type carbon monoxide dehydrogenase, small subunit CoxS/CutS-like protein	777,629	T -> A	1	ACG -> GCG
G	G	G	G	G	G	G	G	G	G	C	G	G	G	81	241	Response regulators consisting of a CheY-like receiver domain and a winged-helix DNA-binding domain protein	780,973	L -> V	1	CTG -> GTG
G	G	G	G	G	C	G	G	G	G	G	G	G	G	58	174	hypothetical protein	797,749	I -> M	3	ATC -> ATG
C	C	C	C	C	C	C	C	C	C	C	T	C	C	9	26	Outer membrane protein V	804,257	A -> V	2	GCC -> GTC
T	C	T	T	T	T	T	T	T	T	T	T	T	T	401	1,202	Arabinose efflux permease	819,57	Q -> R	2	CAG -> CGG
G	G	G	G	A	G	G	G	G	G	A	G	G	G	228	682	Dehydrogenases with different specificities (related to short-chain alcohol dehydrogenases)	827,157	A -> T	1	GCA -> ACA
G	G	G	G	G	G	G	G	G	G	G	G	G	A	194	580	Threonine dehydrogenase and related Zn-dependentdehydrogenase	848,946	G -> S	1	GGT -> AGT
G	G	G	G	G	G	G	G	G	G	C	G	G	G	23	68	Cation transport ATPase	852,088	P -> R	2	CCC -> CGC
C	C	C	C	C	C	C	C	C	C	T	C	C	C	42	124	Acyl-CoA synthetase (NDP forming)	858,524	G -> R	1	GGA -> AGA
A	G	G	G	G	G	G	G	G	G	G	G	G	G	136	407	cAMP-binding proteins - catabolite gene activator and regulatory subunit of cAMP-dependent protein kinase	861,442	I -> T	2	ATT -> ACT
C	C	C	C	C	T	C	C	C	C	C	C	C	C	23	67	Response regulators consisting of a CheY-like receiver domain and a winged-helix DNA-binding domain protein	862,116	R -> C	1	CGC -> TGC
T	C	T	T	T	T	T	T	T	T	T	T	T	T	49	145	hypothetical protein	902,628	Y -> H	1	TAC -> CAC
C	C	C	A	C	C	C	C	C	C	C	C	C	C	311	932	Nucleotidyltransferase/DNA polymerase involved in DNA repair	970,274	R -> L	2	CGC -> CTC
T	T	T	T	T	T	T	T	A	T	T	T	T	T	226	677	Sulfatase	1,041,689	Y -> F	2	TAT -> TTT
G	G	G	G	G	G	G	G	G	G	G	G	G	A	28	82	Co-chaperonin GroES (HSP10)	1,062,684	V -> I	1	GTC -> ATC
T	T	T	T	C	T	T	T	T	T	C	T	T	T	22	64	Protein of unknown function (DUF3141)	1,116,729	F -> L	1	TTC -> CTC
A	A	A	A	A	A	A	A	A	A	A	A	G	A	180	539	putative signal-transduction protein containing cAMP-binding and CBS domains	1,143,745	V -> A	2	GTC -> GCC
G	G	G	G	G	A	G	G	G	G	G	G	G	G	126	377	hypothetical protein	1,145,830	G -> D	2	GGC -> GAC
A	A	A	A	C	A	A	A	A	A	C	A	A	A	1	1	Small-conductance mechanosensitive channel	1,154,882	S -> R	1	AGC -> CGC
A	C	C	C	C	C	C	C	C	C	C	C	C	C	733	2,199	Serine phosphatase RsbU, regulator of sigma subunit	1,227,548	R -> S	3	AGA -> AGC
T	T	T	T	T	T	C	T	T	T	T	T	T	T	220	658	Alcohol dehydrogenase, class IV	1,268,178	F -> L	1	TTC -> CTC
C	C	C	C	C	G	C	C	C	C	C	C	C	C	227	681	ABC-type sugar transport system, periplasmic component	1,305,180	D -> E	3	GAC -> GAG
G	G	G	G	G	G	G	C	G	G	G	G	G	G	58	172	TRAP-type C4-dicarboxylate transport system, periplasmic component	1,330,565	A -> P	1	GCC -> CCC
G	G	G	G	G	G	G	G	G	G	G	T	G	G	12	34	TRAP-type C4-dicarboxylate transport system, small permease component	1,331,498	A -> S	1	GCT -> TCT
C	C	G	C	C	C	C	C	C	C	C	C	C	C	21	62	Lactate dehydrogenase and related dehydrogenase	1,373,122	R -> P	2	CGG -> CCG
