# Supplementary material for: The early events underlying genome evolution in a localized Sinorhizobium meliloti population
Source: BMC Genomics. 2016 Aug 5;17:556. doi: 10.1186/s12864-016-2878-9 (PMC4974801; doi:10.1186/s12864-016-2878-9)
Supplement: Additional file 6: Table S5. — iSNP string sequence. (PDF 283 kb) [file 12864_2016_2878_MOESM6_ESM.pdf]

S5 Table. iSNPs string sequence

Chromosome iSNPs

GR4	G1	G2	G3	G4	G5	G6	G7	G8	G9	G10	G11	G12	G13	Position (original sequence)	Reference 1021
C	C	C	C	C	C	C	C	T	C	C	C	C	C	185,773	
A	A	A	A	A	A	A	A	A	A	A	A	A	C	386,298	
A	A	A	A	G	A	A	A	A	A	A	A	A	A	489,302	
C	C	G	C	C	C	C	C	C	C	C	C	G	C	519,481	
T	T	T	T	T	T	T	T	T	T	T	A	T	T	658,366	SMc_TSS01744
G	G	G	G	G	G	G	G	G	A	G	G	G	G	670,981	
G	G	G	G	G	G	G	G	G	A	G	G	G	G	831,994	
T	T	T	T	T	T	T	T	T	C	T	T	T	T	892,962	
G	A	A	G	A	A	G	A	A	A	A	A	A	A	945,839	SMc_TSS02546
C	C	C	C	T	C	C	C	C	C	C	C	C	C	963,559	SMc_TSS02606
G	G	G	G	A	G	G	G	G	G	G	G	G	G	1,295,496	
A	A	A	A	A	A	A	A	A	A	C	A	A	A	1,443,712	
G	G	G	G	G	A	G	G	G	G	G	G	G	G	1,518,725	
A	A	A	A	A	A	A	A	A	G	A	A	A	A	1,584,308	SMc_TSS04769
G	G	G	G	G	G	G	C	G	G	G	G	G	G	1,637,995	
T	T	T	T	T	T	T	T	T	C	T	T	T	T	1,648,099	
T	T	T	T	T	T	T	T	T	C	T	T	T	T	1,655,241	
A	A	A	A	A	A	A	A	A	C	A	A	A	A	1,769,895	SMc_asRNA_1449
C	C	C	C	C	C	C	A	C	C	C	C	C	C	1,796,670	
G	G	G	G	G	G	G	G	G	G	T	G	G	G	1,796,810	
G	G	G	G	G	G	G	T	G	G	G	G	G	G	1,826,153	
C	C	C	C	C	C	C	C	C	C	C	C	G	C	1,832,411	
C	C	C	C	C	C	C	C	C	C	T	C	C	C	2,009,346	SMc_TSS06143
T	G	G	G	G	G	G	G	G	G	G	G	G	G	2,156,798	
C	C	C	C	C	C	C	C	C	C	C	C	C	G	2,164,303	
T	T	T	T	T	T	T	G	T	T	T	T	T	T	2,200,789	
C	C	C	C	G	C	C	C	C	C	G	C	C	C	2,227,079	
C	C	C	C	T	C	C	C	C	C	T	C	C	C	2,327,888	
C	C	C	C	C	C	C	C	C	C	C	T	C	C	2,461,498	SMc_TSS07257
A	A	A	A	A	A	A	A	A	G	A	A	A	A	2,608,090	
T	T	T	T	T	T	T	T	T	C	T	T	T	T	2,770,949	
A	T	A	A	A	A	A	A	A	A	A	A	A	A	2,885,096	SMc_TSS08189
A	A	A	A	A	A	A	A	A	A	A	A	T	A	2,952,010	SMc_TSS08369
C	A	A	A	A	A	A	A	A	A	A	A	A	A	2,976,408	
G	G	G	G	G	G	G	G	G	G	G	G	G	C	3,008,742	SMc_ncRNA_306
C	C	C	C	C	C	C	C	C	C	C	C	T	C	3,077,592	
T	T	T	T	T	T	T	T	T	T	C	T	T	T	3,217,240	SMc_asRNA_2028
T	T	T	T	T	T	T	T	C	T	T	T	T	T	3,301,157	
G	G	G	G	G	G	G	G	G	G	G	G	T	G	3,332,156	
C	C	C	C	T	C	C	C	C	C	C	C	C	C	3,579,400	
T	T	T	A	T	T	T	T	T	T	T	T	T	T	3,615,896	SMc_TSS10440

pSymB iSNPs

GR4	G1	G2	G3	G4	G5	G6	G7	G8	G9	G10	G11	G12	G13	Position (original sequence)	Reference 1021
T	T	T	T	T	T	C	T	T	T	T	T	T	T	61,021	
G	G	A	G	G	G	G	G	G	G	G	G	G	G	257,537	
C	C	C	A	C	C	C	C	C	C	C	C	C	C	350,451	SMb_TSS3056
C	T	T	C	T	T	C	T	T	T	T	T	T	T	464,016	
C	C	C	C	C	C	G	C	C	C	C	C	C	C	499,166	
T	T	T	T	T	C	T	T	T	T	T	T	T	T	539,051	
G	G	G	G	G	G	G	G	G	G	G	G	T	G	626,549	SMb_asRNA_2772
T	G	G	T	G	G	T	G	G	G	G	G	G	G	785,983	
G	G	G	G	G	G	G	G	G	A	G	G	G	G	801,055	SMb_asRNA_2688
T	T	T	T	T	T	T	C	T	T	T	T	T	T	842,49	
G	G	A	G	G	G	G	G	G	G	G	G	G	G	1,008,880	
T	G	G	G	T	G	G	G	G	G	T	G	G	G	1,013,670	
A	G	G	G	G	G	G	G	G	G	G	G	G	G	1,059,297	
T	G	G	T	G	G	T	G	G	G	G	G	T	G	1,069,197	
C	T	T	T	T	T	T	T	T	T	T	T	T	T	1,094,103	
G	G	G	G	G	G	G	G	G	A	G	G	G	G	1,108,326	
G	G	G	G	C	G	G	G	G	G	G	G	G	G	1,276,070	
G	G	G	G	G	G	G	G	G	G	G	A	G	G	1,430,024	
C	C	G	C	C	C	C	C	C	C	C	C	C	C	1,604,575	
T	T	T	T	T	T	T	T	C	T	T	T	T	T	1,690,068	

pSymA iSNPs

GR4	G1	G2	G3	G4	G5	G65	G7	G8	G9	G10	G11	G12	G13	Position (original sequence)	Reference 1021
C	C	C	C	C	T	C	T	C	C	C	C	C	C	47,695	
A	A	A	A	A	A	A	G	A	A	A	A	A	A	74,519	
A	A	A	A	A	A	A	A	A	G	A	A	A	A	82,237	
G	A	A	A	A	A	A	A	A	A	A	A	A	A	279,592	
A	A	A	T	A	A	A	A	A	A	A	A	A	A	295,795	
G	G	G	G	G	G	G	G	C	G	G	G	G	G	306,732	
A	A	A	A	A	A	T	A	A	A	A	A	A	A	335,157	
T	A	A	A	A	A	A	A	A	A	A	A	A	A	335,704	
C	C	C	C	C	C	C	T	C	C	C	C	C	C	393,868	SMa1828
A	A	A	A	C	A	A	A	A	A	C	C	A	A	405,496	
C	C	C	G	C	C	C	C	C	C	C	C	C	C	495,904	
T	T	T	T	T	T	T	T	C	T	T	T	T	T	499,116	
T	T	T	C	T	T	T	T	T	T	T	T	T	T	553,917	
G	T	T	T	T	T	T	T	T	T	T	T	T	T	569,644	SMa_TSS2118
A	A	A	A	A	A	A	A	A	G	A	A	A	A	658,940	
G	G	G	G	G	A	G	G	G	G	G	G	G	G	768,263	
T	C	C	C	C	C	C	C	C	C	C	C	C	C	774,652	
G	G	G	G	G	G	G	G	G	G	C	G	G	G	823,544	
C	C	C	C	C	C	C	C	C	G	C	C	C	C	974,925	
A	A	A	A	A	A	A	A	C	A	A	A	A	A	1,118,209	
C	T	C	C	C	C	C	C	C	C	C	C	C	C	1,311,201	SMa_ncRNA_13
A	A	A	A	A	T	A	T	A	A	A	A	A	A	1,349,525	

RNA features
mTSS
mTSS
mTSS
mTSS
cis-encoded antisense sRNA
mTSS
mTSS
pmTSS
mTSS
trans-encoded sRNA
cis-encoded antisense sRNA
mTSS

RNA features
mTSS
cis-encoded antisense sRNA
cis-encoded antisense sRNA

RNA features
Transcriptional regulator, LysR family
mTSS
trans-encoded sRNA
