# Supplementary material for: The early events underlying genome evolution in a localized Sinorhizobium meliloti population
Source: BMC Genomics. 2016 Aug 5;17:556. doi: 10.1186/s12864-016-2878-9 (PMC4974801; doi:10.1186/s12864-016-2878-9)
Supplement: Additional file 9: Table S6. — Concatenated genes carrying sSNPs. (PDF 72 kb) [file 12864_2016_2878_MOESM9_ESM.pdf]

S6 Table. Concatenated genes carrying sSNPs

Name	Length	locus_tag[a]	protein_id
Transcriptional regulator CDS	852	C770_GR4Chr0085	YP_007188669.1
Flp pilus assembly protein TadB CDS	1,011	C770_GR4Chr0151	YP_007188735.1
putative dehydrogenase-related protein CDS	1,065	C770_GR4Chr0199	YP_007188783.1
BA14K-like protein CDS	528	C770_GR4Chr0267	YP_007188844.1
exodeoxyribonuclease VII, large subunit CDS	1,581	C770_GR4Chr0306	YP_007188881.1
The (Largely Gram-negative Bacterial) Hydrophobe/Amphiphile Efflux-1 (HAE1) Family CDS	3,186	C770_GR4Chr0434	YP_007189009.1
Transcriptional regulator/sugar kinase CDS	1,23	C770_GR4Chr0485	YP_007189059.1
phosphate regulon sensor kinase PhoR CDS	1,263	C770_GR4Chr0505	YP_007189078.1
putative esterase of the alpha-beta hydrolase superfamily CDS	885	C770_GR4Chr0800	YP_007189367.1
tetraacyldisaccharide 4-kinase CDS	1,041	C770_GR4Chr0809	YP_007189376.1
PAS domain S-box/diguanylate cyclase (GGDEF) domain protein CDS	2,319	C770_GR4Chr0962	YP_007189523.1
NADH:ubiquinone oxidoreductase subunit 5 (chain L)/Multisubunit Na+/H+ antiporter, MnhA subu	2,376	C770_GR4Chr0995	YP_007189556.1
type I secretion membrane fusion protein, HlyD family CDS	1,206	C770_GR4Chr1108	YP_007189668.1
Branched-chain amino acid ABC-type transport system, permease component CDS	897	C770_GR4Chr1177	YP_007189737.1
Delta-aminolevulinic acid dehydratase CDS	1,014	C770_GR4Chr1216	YP_007189776.1
riboflavin synthase, alpha subunit CDS	621	C770_GR4Chr1223	YP_007189783.1
Protein-disulfide isomerase CDS	768	C770_GR4Chr1337	YP_007189893.1
hypothetical protein CDS	1,023	C770_GR4Chr1424	YP_007189975.1
Protein-L-isoaspartate carboxylmethyltransferase CDS	660	C770_GR4Chr1532	YP_007190083.1
ABC-type spermidine/putrescine transport systems, ATPase component CDS	1,11	C770_GR4Chr1547	YP_007190098.1
carbamoyl-phosphate synthase, large subunit CDS	3,492	C770_GR4Chr1601	YP_007190147.1
Trk-type K+ transport systems, membrane component CDS	1,455	C770_GR4Chr1666	YP_007190208.1
hypothetical protein CDS	591	C770_GR4Chr1667	YP_007190209.1
ABC-type uncharacterized transport system, permease component CDS	1,095	C770_GR4Chr1710	YP_007190251.1
hypothetical protein CDS	855	C770_GR4Chr1729	YP_007190269.1
putative oxidoreductases (related to aryl-alcohol dehydrogenases) CDS	1,029	C770_GR4Chr1976	YP_007190503.1
3-oxoacyl-(acyl-carrier-protein) synthase CDS	1,284	C770_GR4Chr1993	YP_007190520.1
putative integral membrane protein CDS	1,77	C770_GR4Chr2102	YP_007190628.1
ABC-type sugar transport systems, ATPase component CDS	1,041	C770_GR4Chr2106	YP_007190632.1
Acetyltransferase, including N-acetylase of ribosomal protein CDS	576	C770_GR4Chr2173	YP_007190699.1
TonB-dependent siderophore receptor CDS	2,211	C770_GR4Chr2281	YP_007190804.1
ABC-type sugar transport systems, permease component CDS	924	C770_GR4Chr2297	YP_007190820.1
Acyl dehydratase CDS	471	C770_GR4Chr2465	YP_007190983.1
hypothetical protein CDS	1,224	C770_GR4Chr2493	YP_007191011.1
Cation/multidrug efflux pump CDS	3,312	C770_GR4Chr2564	YP_007191082.1
ABC-type dipeptide transport system, periplasmiccomponent CDS	1,623	C770_GR4Chr2693	YP_007191209.1
hypothetical protein CDS	255	C770_GR4Chr2817	YP_007191324.1
hypothetical protein CDS	189	C770_GR4Chr2818	YP_007191325.1
Permeases of the drug/metabolite transporter (DMT) superfamily CDS	891	C770_GR4Chr2853	YP_007191359.1
Arabinose efflux permease CDS	1,302	C770_GR4Chr2897	YP_007191399.1
pyruvate kinase CDS	1,44	C770_GR4Chr2916	YP_007191418.1
Response regulator containing CheY-like receiver, AAA-type ATPase, and DNA-binding domains C	363	C770_GR4Chr2953	YP_007191454.1
succinyl-CoA synthetase, beta subunit CDS	1,197	C770_GR4Chr3192	YP_007191680.1
C-terminal peptidase (prc) CDS	1,323	C770_GR4Chr3248	YP_007191735.1
thiamine ABC transporter, permease protein CDS	1,608	C770_GR4Chr3348	YP_007191827.1
hypothetical protein CDS	1,401	C770_GR4Chr3379	YP_007191856.1
FAD/FMN-containing dehydrogenase CDS	1,422	C770_GR4Chr3380	YP_007191857.1
Choline dehydrogenase-related flavoprotein CDS	1,509	C770_GR4Chr3389	YP_007191866.1
DNA polymerase III, delta subunit CDS	1,032	C770_GR4Chr3429	YP_007191904.1
putative dehydrogenase-related protein CDS	1,245	C770_GR4pD0035	YP_007193760.1
ABC-type sugar transport system, permease component CDS	864	C770_GR4pD0077	YP_007193802.1
Ribose/xylose/arabinose/galactoside ABC-type transport systems, permease component CDS	1,047	C770_GR4pD0088	YP_007193813.1
thiamine biosynthesis protein ThiS CDS	198	C770_GR4pD0093	YP_007193818.1
Transcriptional regulator CDS	891	C770_GR4pD0194	YP_007193918.1
hypothetical protein CDS	1,149	C770_GR4pD0274	YP_007193998.1
cytochrome o ubiquinol oxidase, subunit I CDS	2,01	C770_GR4pD0298	YP_007194022.1
hypothetical protein CDS	1,278	C770_GR4pD0331	YP_007194051.1
4-aminobutyrate aminotransferase and related aminotransferase CDS	3,027	C770_GR4pD0489	YP_007194206.1
Glycine/D-amino acid oxidases (deaminating) CDS	1,401	C770_GR4pD0616	YP_007194331.1
ABC-type dipeptide/oligopeptide/nickel transportsystems, permease component CDS	924	C770_GR4pD0717	YP_007194430.1
ABC-type proline/glycine betaine transport systems, ATPase component CDS	939	C770_GR4pD0766	YP_007194477.1
amidohydrolase CDS	1,17	C770_GR4pD0797	YP_007194507.1
nucleotide sugar dehydrogenase CDS	1,35	C770_GR4pD0940	YP_007194650.1
ribose 5-phosphate isomerase B CDS	447	C770_GR4pD1231	YP_007194933.1
Protein of unknown function (DUF3365) CDS	759	C770_GR4pD1247	YP_007194949.1
NAD-dependent aldehyde dehydrogenase CDS	1,518	C770_GR4pD1335	YP_007195037.1
bifunctional S-(hydroxymethyl)glutathione dehydrogenase/class III alcohol dehydrogenase CDS	1,128	C770_GR4pD1419	YP_007195121.1
Aerobic-type carbon monoxide dehydrogenase, large subunit CoxL/CutL-like protein CDS	2,319	C770_GR4pD1462	YP_007195164.1
ABC-type uncharacterized transport system, permease component CDS	1,098	C770_GR4pD1468	YP_007195170.1
Erythromycin esterase-like protein CDS	1,977	C770_GR4pD1505	YP_007195207.1
ABC-type Fe3+-siderophore transport system, permease component CDS	1,044	C770_GR4pD1546	YP_007195244.1
Short-chain alcohol dehydrogenase of unknown specificity CDS	723	C770_GR4pC0033	YP_007192373.1
putative redox protein, regulator of disulfide bond formation CDS	513	C770_GR4pC0148	YP_007192486.1
GTPase subunit of restriction endonuclease CDS	2,247	C770_GR4pC0241	YP_007192573.1
ABC-type dipeptide transport system, periplasmiccomponent CDS	1,611	C770_GR4pC0374	YP_007192698.1
hypothetical protein CDS	651	C770_GR4pC0480	YP_007192802.1
Flp pilus assembly protein, ATPase CpaF CDS	1,431	C770_GR4pC0519	YP_007192841.1
Collagenase and related protease CDS	936	C770_GR4pC0688	YP_007193004.1
Ornithine carbamoyltransferase CDS	1,026	C770_GR4pC0866	YP_007193172.1
Ti-type conjugative transfer system protein TraG CDS	1,92	C770_GR4pC0965	YP_007193260.1
hypothetical protein CDS	471	C770_GR4pC1119	YP_007193406.1
Multidrug resistance efflux pump CDS	1,23	C770_GR4pC1137	YP_007193424.1
hypothetical protein CDS	246	C770_GR4pC1211	YP_007193496.1
Transcriptional regulator containing an amidase domain and an AraC-type DNA-binding HTH doma	960	C770_GR4pC1249	YP_007193532.1
Ribose/xylose/arabinose/galactoside ABC-type transport systems, permease component CDS	1,02	C770_GR4pC1316	YP_007193599.1
amine acid ABC transporter, permease protein, 3-TM region, His/Glu/Gln/Arg/opine family CDS	657	C770_GR4pC1401	YP_007193682.1

[a] GR4Chr: Chromosomal genes; GR4pD: pSymB genes; GR4pC: pSymA genes
