# Supplementary material for: The early events underlying genome evolution in a localized Sinorhizobium meliloti population
Source: BMC Genomics. 2016 Aug 5;17:556. doi: 10.1186/s12864-016-2878-9 (PMC4974801; doi:10.1186/s12864-016-2878-9)
Supplement: Additional file 10: Table S7. — Concatenated genes carrying nsSNPs. (PDF 77 kb) [file 12864_2016_2878_MOESM10_ESM.pdf]

S7 Table. Concatenated genes carrying nsSNPs

Name	Length	locus_tag[a]	protein_id
putative metal-binding integral membrane protein CDS	885	C770_GR4Chr0188	YP_007188772.1
N-acetylglucosamine-6-phosphate deacetylase CDS	1,161	C770_GR4Chr0207	YP_007188791.1
DNA polymerase III, subunit gamma and tau CDS	1,881	C770_GR4Chr0238	YP_007188816.1
Ketosteroid isomerase-like protein CDS	867	C770_GR4Chr0381	YP_007188956.1
Methyl-accepting chemotaxis protein CDS	2,367	C770_GR4Chr0425	YP_007189000.1
Capsule polysaccharide export protein CDS	1,326	C770_GR4Chr0568	YP_007189139.1
flagellar basal-body rod protein FlgF CDS	726	C770_GR4Chr0645	YP_007189214.1
FAD/FMN-containing dehydrogenase CDS	1,44	C770_GR4Chr0773	YP_007189340.1
hypothetical protein CDS	639	C770_GR4Chr0818	YP_007189385.1
putative NAD(P)H quinone oxidoreductase, PIG3 family CDS	1,002	C770_GR4Chr0955	YP_007189517.1
Dehydrogenases (flavoproteins) CDS	1,665	C770_GR4Chr1040	YP_007189601.1
Cu/Zn superoxide dismutase CDS	519	C770_GR4Chr1105	YP_007189665.1
Molybdopterin converting factor, large subunit CDS	468	C770_GR4Chr1178	YP_007189738.1
bifunctional exopolyphosphatase CDS	1,524	C770_GR4Chr1197	YP_007189757.1
hypothetical protein CDS	1,191	C770_GR4Chr1210	YP_007189770.1
hypothetical protein CDS	351	C770_GR4Chr1224	YP_007189784.1
3-oxoacyl-(acyl-carrier-protein) synthase III CDS	972	C770_GR4Chr1235	YP_007189795.1
Glycosyltransferase CDS	1,245	C770_GR4Chr1243	YP_007189802.1
putative CoA-binding protein CDS	429	C770_GR4Chr1262	YP_007189821.1
Cytochrome P450 CDS	1,248	C770_GR4Chr1265	YP_007189824.1
protein CrcB CDS	378	C770_GR4Chr1407	YP_007189959.1
Putative protein-S-isoprenylcysteine methyltransferase CDS	474	C770_GR4Chr1423	YP_007189974.1
hypothetical protein CDS	1,023	C770_GR4Chr1424	YP_007189975.1
hypothetical protein CDS	432	C770_GR4Chr1451	YP_007190002.1
NADH:flavin oxidoreductase, Old Yellow Enzyme family CDS	1,122	C770_GR4Chr1587	YP_007190135.1
hypothetical protein CDS	786	C770_GR4Chr1615	YP_007190161.1
hypothetical protein CDS	1,488	C770_GR4Chr1641	YP_007190186.1
5-nucleotidase/2,3-cyclic phosphodiesterase and related esterase CDS	1,974	C770_GR4Chr1663	YP_007190205.1
ABC-type oligopeptide transport system, periplasmic component CDS	1,815	C770_GR4Chr1682	YP_007190223.1
Molybdopterin biosynthesis enzyme CDS	1,221	C770_GR4Chr1704	YP_007190245.1
MoxR-like ATPase CDS	930	C770_GR4Chr1803	YP_007190333.1
phosphoribosylformylglycinamidine synthase II CDS	2,232	C770_GR4Chr1829	YP_007190359.1
hypothetical protein CDS	1,464	C770_GR4Chr1847	YP_007190377.1
putative flavoprotein CDS	573	C770_GR4Chr1878	YP_007190406.1
Dehydrogenases with different specificities (related to short-chain alcohol dehydrogenases) CDS	822	C770_GR4Chr1898	YP_007190425.1
bifunctional cobyrinic acid a,c-diamide synthase CDS	1,29	C770_GR4Chr1999	YP_007190526.1
hypothetical protein CDS	909	C770_GR4Chr2015	YP_007190542.1
putative addiction module antidote protein, CC2985 family CDS	258	C770_GR4Chr2022	YP_007190549.1
HAD-superfamily hydrolase, subfamily IIB CDS	1,713	C770_GR4Chr2105	YP_007190631.1
diguanylate cyclase (GGDEF) domain protein CDS	1,23	C770_GR4Chr2202	YP_007190727.1
lytic murein transglycosylase CDS	1,218	C770_GR4Chr2254	YP_007190778.1
L-threonine 3-dehydrogenase CDS	1,035	C770_GR4Chr2421	YP_007190939.1
Transcriptional activator of acetoin/glycerol metabolism CDS	957	C770_GR4Chr2457	YP_007190975.1
Outer membrane receptor for Fe3+-dicitrate CDS	2,799	C770_GR4Chr2487	YP_007191005.1
TonB-dependent heme/hemoglobin receptor family protein/TonB-dependent hemoglobin/transferrin/lactoferrin receptor family protein CDS	2,232	C770_GR4Chr2490	YP_007191008.1
putative transcriptional regulator CDS	345	C770_GR4Chr2505	YP_007191023.1
Uncharacterized protein conserved in bacteria (DUF2325) CDS	414	C770_GR4Chr2526	YP_007191044.1
ABC-type hemin transport system, ATPase component CDS	789	C770_GR4Chr2532	YP_007191050.1
urease, alpha subunit CDS	1,713	C770_GR4Chr2572	YP_007191090.1
ABC-type branched-chain amino acid transport systems, ATPase component CDS	888	C770_GR4Chr2585	YP_007191103.1
ABC-type dipeptide/oligopeptide/nickel transportsystems, permease component CDS	819	C770_GR4Chr2641	YP_007191159.1
Dehydrogenases with different specificities (related to short-chain alcohol dehydrogenases) CDS	795	C770_GR4Chr2651	YP_007191169.1
Glycerol dehydrogenase-related enzyme CDS	1,095	C770_GR4Chr2652	YP_007191170.1
Short-chain alcohol dehydrogenase of unknown specificity CDS	729	C770_GR4Chr2653	YP_007191171.1
ABC-type branched-chain amino acid transport systems, periplasmic component CDS	1,173	C770_GR4Chr2685	YP_007191201.1
ABC-type dipeptide/oligopeptide/nickel transportsystems, permease component CDS	951	C770_GR4Chr2697	YP_007191213.1
Membrane proteins related to metalloendopeptidase CDS	1,941	C770_GR4Chr2717	YP_007191225.1
Cell division protein CDS	1,017	C770_GR4Chr2768	YP_007191275.1
hypothetical protein CDS	1,443	C770_GR4Chr2795	YP_007191302.1
hypothetical protein CDS	903	C770_GR4Chr3014	YP_007191513.1
Inosine-uridine nucleoside N-ribohydrolase CDS	954	C770_GR4Chr3034	YP_007191533.1
bifunctional precorrin-6y C5,15-methyltransferase (decarboxylating), CbiE subunit/precorrin-6Y C5,15-methyltransferase (decarboxylating), CbiT subunit CDS	1,191	C770_GR4Chr3048	YP_007191545.1
Transcriptional regulator CDS	480	C770_GR4Chr3071	YP_007191568.1
ABC-type dipeptide transport system, periplasmiccomponent CDS	1,629	C770_GR4Chr3098	YP_007191595.1
hypothetical protein CDS	5,61	C770_GR4Chr3135	YP_007191623.1
Aminoglycoside phosphotransferase CDS	786	C770_GR4Chr3137	YP_007191625.1
hypothetical protein CDS	501	C770_GR4Chr3187	YP_007191675.1
hypothetical protein CDS	825	C770_GR4Chr3235	YP_007191723.1
nicotinate (nicotinamide) nucleotide adenylyltransferase CDS	564	C770_GR4Chr3244	YP_007191731.1
DNA-directed DNA polymerase III (polc) CDS	3,351	C770_GR4Chr3253	YP_007191740.1
hypothetical protein CDS	504	C770_GR4Chr3302	YP_007191786.1
NAD-dependent aldehyde dehydrogenase CDS	1,509	C770_GR4Chr3394	YP_007191871.1
ABC-type dipeptide/oligopeptide/nickel transportsystems, permease component CDS	1,128	C770_GR4pD0058	YP_007193783.1
thiamine-phosphate pyrophosphorylase CDS	606	C770_GR4pD0091	YP_007193816.1
hypothetical protein CDS	2,019	C770_GR4pD0097	YP_007193821.1
urea ABC transporter, permease protein UrtC CDS	1,176	C770_GR4pD0111	YP_007193835.1
Pyridoxal phosphate biosynthesis protein CDS	1,047	C770_GR4pD0153	YP_007193877.1
Membrane protein involved in colicin uptake CDS	945	C770_GR4pD0233	YP_007193957.1
TRAP-type C4-dicarboxylate transport system, small permease component CDS	477	C770_GR4pD0350	YP_007194070.1
ABC-type sugar transport system, ATPase component CDS	1,536	C770_GR4pD0403	YP_007194120.1
hypothetical protein CDS	1,395	C770_GR4pD0411	YP_007194128.1
RND family efflux transporter, MFP subunit CDS	1,407	C770_GR4pD0415	YP_007194132.1
xanthine dehydrogenase, small subunit CDS	1,488	C770_GR4pD0453	YP_007194170.1
ABC-type amino acid transport system, permease component CDS	1,65	C770_GR4pD0487	YP_007194204.1
phosphonoacetate hydrolase CDS	1,275	C770_GR4pD0607	YP_007194322.1
Na+/phosphate symporter CDS	1,671	C770_GR4pD0609	YP_007194324.1
ABC-type sugar transport system, periplasmic component CDS	891	C770_GR4pD0629	YP_007194344.1
hypothetical protein CDS	453	C770_GR4pD0641	YP_007194356.1
Glycosyltransferase CDS	2,682	C770_GR4pD0684	YP_007194399.1
ABC-type dipeptide transport system, periplasmiccomponent CDS	1,59	C770_GR4pD0698	YP_007194413.1
Lactoylglutathione lyase and related lyase CDS	933	C770_GR4pD0711	YP_007194426.1
phosphonate ABC transporter, permease protein PhnE CDS	963	C770_GR4pD0736	YP_007194449.1
phosphonate metabolism protein/1,5-bisphosphokinase (PRPP-forming) PhnN CDS	588	C770_GR4pD0740	YP_007194453.1
Putative translation initiation inhibitor, yjgF family CDS	390	C770_GR4pD0755	YP_007194466.1
Periplasmic glycine betaine/choline-binding (lipo)protein of an ABC-type transport system (osmoprotectant binding protein) CDS	918	C770_GR4pD0768	YP_007194479.1
NAD-dependent aldehyde dehydrogenase CDS	2,385	C770_GR4pD0774	YP_007194485.1
Polysaccharide deacetylase CDS	771	C770_GR4pD0821	YP_007194531.1
putative pyridoxal phosphate-dependent enzyme apparently involved in regulation of cell wall biogenesis CDS	1,191	C770_GR4pD0826	YP_007194536.1
Hemolysin-type calcium-binding repeat (2 copies) CDS	1,164	C770_GR4pD0842	YP_007194552.1
hypothetical protein CDS	555	C770_GR4pD0869	YP_007194579.1
Acetyl/propionyl-CoA carboxylase, alpha subunit CDS	1,989	C770_GR4pD0873	YP_007194583.1
putative metal-dependent hydrolase of the TIM-barrel fold protein CDS	837	C770_GR4pD0894	YP_007194604.1
ABC-type amino acid transport/signal transduction systems, periplasmic component/domain protein CDS	843	C770_GR4pD0897	YP_007194607.1
hypothetical protein CDS	558	C770_GR4pD0959	YP_007194669.1
hypothetical protein CDS	1,071	C770_GR4pD1150	YP_007194852.1
diguanylate cyclase (GGDEF) domain protein CDS	1,026	C770_GR4pD1213	YP_007194915.1
hypothetical protein CDS	729	C770_GR4pD1217	YP_007194919.1
NAD-dependent aldehyde dehydrogenase CDS	1,464	C770_GR4pD1224	YP_007194926.1
TRAP transporter, 4TM/12TM fusion protein CDS	2,499	C770_GR4pD1305	YP_007195007.1
Threonine dehydrogenase and related Zn-dependentdehydrogenase CDS	993	C770_GR4pD1351	YP_007195053.1
PQQ-dependent catabolism-associated CXXCW motif protein CDS	546	C770_GR4pD1413	YP_007195115.1
ABC-type uncharacterized transport systems, ATPase component CDS	1,527	C770_GR4pD1470	YP_007195172.1
Thiamine pyrophosphate-requiring enzymes CDS	1,818	C770_GR4pD1498	YP_007195200.1
TonB-dependent siderophore receptor CDS	2,241	C770_GR4pC0036	YP_007192376.1
putative integral membrane protein CDS	2,001	C770_GR4pC0147	YP_007192485.1
Spermidine/putrescine-binding periplasmic protein CDS	1,116	C770_GR4pC0179	YP_007192517.1
Multidrug resistance efflux pump CDS	1,035	C770_GR4pC0228	YP_007192560.1
Transcriptional regulator CDS	894	C770_GR4pC0274	YP_007192602.1
Acetyltransferase (GNAT) family CDS	372	C770_GR4pC0279	YP_007192607.1
Transcriptional regulator CDS	984	C770_GR4pC0298	YP_007192626.1
putative ornithine cyclodeaminase CDS	606	C770_GR4pC0367	YP_007192691.1
Transcriptional regulator CDS	924	C770_GR4pC0563	YP_007192883.1
ABC-type dipeptide transport system, periplasmiccomponent CDS	1,509	C770_GR4pC0591	YP_007192911.1
ABC-type sugar transport system, periplasmic component CDS	1,113	C770_GR4pC0598	YP_007192918.1
Dehydrogenases with different specificities (related to short-chain alcohol dehydrogenases) CDS	780	C770_GR4pC0613	YP_007192933.1
Sugar phosphate isomerase/epimerase CDS	852	C770_GR4pC0646	YP_007192964.1
hypothetical protein CDS	1,197	C770_GR4pC0658	YP_007192976.1
type IV secretion/conjugal transfer ATPase, VirB4 family CDS	2,397	C770_GR4pC0673	YP_007192989.1
Nitric oxide reductase large subunit CDS	1,347	C770_GR4pC0699	YP_007193015.1
periplasmic nitrate reductase, large subunit CDS	2,505	C770_GR4pC0720	YP_007193036.1
cytochrome c oxidase accessory protein FixG CDS	1,575	C770_GR4pC0734	YP_007193048.1
Aerobic-type carbon monoxide dehydrogenase, small subunit CoxS/CutS-like protein CDS	471	C770_GR4pC0776	YP_007193088.1
Response regulators consisting of a CheY-like receiver domain and a winged-helix DNA-binding domain protein CDS	720	C770_GR4pC0780	YP_007193092.1
hypothetical protein CDS	297	C770_GR4pC0802	YP_007193110.1
Outer membrane protein V CDS	861	C770_GR4pC0810	YP_007193118.1
Arabinose efflux permease CDS	1,326	C770_GR4pC0823	YP_007193131.1
Dehydrogenases with different specificities (related to short-chain alcohol dehydrogenases) CDS	915	C770_GR4pC0831	YP_007193139.1
Threonine dehydrogenase and related Zn-dependentdehydrogenase CDS	1,074	C770_GR4pC0852	YP_007193160.1
Cation transport ATPase CDS	2,652	C770_GR4pC0853	YP_007193161.1
Acyl-CoA synthetase (NDP forming) CDS	2,7	C770_GR4pC0859	YP_007193167.1
cAMP-binding proteins - catabolite gene activator and regulatory subunit of cAMP-dependent protein kinase CDS	750	C770_GR4pC0863	YP_007193169.1
Response regulators consisting of a CheY-like receiver domain and a winged-helix DNA-binding domain protein CDS	672	C770_GR4pC0864	YP_007193170.1
hypothetical protein CDS	1,368	C770_GR4pC0913	YP_007193210.1
Nucleotidyltransferase/DNA polymerase involved in DNA repair CDS	1,344	C770_GR4pC0986	YP_007193281.1
Sulfatase CDS	1,662	C770_GR4pC1063	YP_007193352.1
Co-chaperonin GroES (HSP10) CDS	297	C770_GR4pC1089	YP_007193378.1
Protein of unknown function (DUF3141) CDS	198	C770_GR4pC1148	YP_007193435.1
putative signal-transduction protein containing cAMP-binding and CBS domains CDS	672	C770_GR4pC1169	YP_007193456.1
hypothetical protein CDS	1,26	C770_GR4pC1172	YP_007193459.1
Small-conductance mechanosensitive channel CDS	1,443	C770_GR4pC1179	YP_007193466.1
Serine phosphatase RsbU, regulator of sigma subunit CDS	2,361	C770_GR4pC1247	YP_007193530.1
Alcohol dehydrogenase, class IV CDS	1,2	C770_GR4pC1291	YP_007193574.1
ABC-type sugar transport system, periplasmic component CDS	1,098	C770_GR4pC1326	YP_007193607.1
TRAP-type C4-dicarboxylate transport system, periplasmic component CDS	1,005	C770_GR4pC1353	YP_007193634.1
TRAP-type C4-dicarboxylate transport system, small permease component CDS	570	C770_GR4pC1354	YP_007193635.1
Lactate dehydrogenase and related dehydrogenase CDS	939	C770_GR4pC1398	YP_007193679.1

[a] GR4Chr: Chromosomal genes; GR4pD: pSymB genes; GR4pC: pSymA genes
