# Supplementary material for: The early events underlying genome evolution in a localized Sinorhizobium meliloti population
Source: BMC Genomics. 2016 Aug 5;17:556. doi: 10.1186/s12864-016-2878-9 (PMC4974801; doi:10.1186/s12864-016-2878-9)
Supplement: Additional file 11: Table S8. — Deletions (4-63 nts) in the GR4-type isolates. (PDF 66 kb) [file 12864_2016_2878_MOESM11_ESM.pdf]

S8 Table. Deletions in the GR4-type isolates

Replicon	Strain/Isolates	Deletions	nº bp	Coordinate positions	Locus	Name	Annotation	Mutation
**Chromosome**	GR4	1	15	671,852/671,853	GR4Chr0624	*icpA*/*mcpE*	Methyl-accepting internal chemotaxis protein	missing residues 178-182 (RQQHQ)
		2	11	3,530,664/3,530,665	GR4Chr3372	*expR*		frame-shift mutation
	G1	1	51	980,206 to 980,256	IGR			3´UTR of a hypothetical protein (GR4Chr0922) and a periplasmic component of an ABC-type branched-chain amino acid transport systems (GR4Chr0923)
		2	23	2,608,245 to 2,608,267	IGR			possible 5´UTR of an outer membrane receptor for Fe3+-dicitrate (GR4Chr2487)
	G2	1	6	951,122 to 951,127	GR4Chr0892		D-alanyl-D-alanine carboxypeptidase	missing residues 75-76 (PV)
	G5	1	13	1,503,118 to 1,503,130	IGR			5´UTR of a putative*folE*- GTP cyclohydrolase I
	G8	1	23	1,500,804 to 1,500,826	GR4Chr1438		putative esterase of the alpha-beta hydrolase superfamily	frame-shift mutation
	G9	1	15	1,072,278 to 1,077,292	GR4Chr1022		surface antigen	missing residues 24-28 (GTTSS)
	G11	1	63	1,603,257 to 1,603,319	IGR			3´UTR (mapped by RNAseq in strain 2011) of a hypothetical protein (GR4Chr1534)
**pSymB**	G3	1	4	1,116,635 to 1,116,638	GR4pD0996		ABC-type sugar transport system, periplasmic component	frame-shift mutation
	G10	1	13	1,154,926 to 1,154,938	IGR			IGR beetween 5´UTRs of Fatty acid desaturase (GR4pD1030) and ABC-type sugar transport system, periplasmic component (GR4pD1031)
**pSymA**	G4	1	16	161,998 to 162,013	IGR			CT/GA-MRI
	G5	1	10	162,036 to 162,045	IGR			CT/GA-MRI
	G9	1	15	162,032 to 162,045	IGR			CT/GA-MRI
	G13	1	8	162,038 to 162,045	IGR			CT/GA-MRI
		2	12	169,122 to 169,133	GR4pC0161		Response regulator	frame-shift mutation
		3	12	225,979 to 225,290	GR4pC0219		oligopeptide/dipeptide ABC transporter, ATP-binding protein, C-terminal domain protein	missing residues 110-113 (QDPM)
