# Supplementary material for: The early events underlying genome evolution in a localized Sinorhizobium meliloti population
Source: BMC Genomics. 2016 Aug 5;17:556. doi: 10.1186/s12864-016-2878-9 (PMC4974801; doi:10.1186/s12864-016-2878-9)
Supplement: Additional file 12: Table S9. — Small indels in the chromosome of the GR4-type isolates. (PDF 66 kb) [file 12864_2016_2878_MOESM12_ESM.pdf]

S9 Table. Chromosome indels

Isolate	Length	Change	Coverage	Polymorphism Type	Variant Frequency	Sequence	CDS Codon Number	CDS Position	product	Min (original sequence)	Max (original sequence)	Amino Acid Change	CDS Position Within Codon	Codon Change	Strand-Bias
**G1**	0		88	Insertion	100.0%	---	107	321	Tellurite resistance protein TerB	558,406	558,406	R -> RL	3	CGG -> CGG,TTG	50.0% -> 50.6%
**G1**	1	(C)5 -> (C)4	33	Deletion (tandem repeat)	100.0%	C				1,193,210	1,193,210				54.5%
**G1**	1	(A)4 -> (A)3	46	Deletion (tandem repeat)	100.0%	A				1,193,328	1,193,328				54.3%
**G1**	1		61	Deletion	100.0%	T				2,149,891	2,149,891				50.8%
**G1**	3	TTC -> AGG	7	Substitution	100.0%	TTC				2,608,265	2,608,267				62.5% -> 71.4%
**G1**	2	(CC)6 -> (CC)5	34	Deletion (tandem repeat)	97.1% -> 100.0%	CC				2,797,449	2,797,450				66.7% -> 67.6%
**G2**	0		77	Insertion	100.0%	---	107	321	Tellurite resistance protein TerB	558,406	558,406	R -> RL	3	CGG -> CGG,TTG	55.8%
**G2**	1	(C)5 -> (C)4	46	Deletion (tandem repeat)	100.0%	C				1,193,210	1,193,210				52.2%
**G2**	1	(A)4 -> (A)3	44	Deletion (tandem repeat)	100.0%	A				1,193,328	1,193,328				59.1%
**G2**	1	-C	73	Deletion	100.0%	C	98	294	hypothetical protein	2,696,005	2,696,005		3		54.8%
**G2**	1		67	Deletion	100.0%	T				2,776,231	2,776,231				52.2%
**G2**	2	(CC)6 -> (CC)5	48	Deletion (tandem repeat)	100.0%	CC				2,797,449	2,797,450				56.3%
**G3**	0		70	Insertion	100.0%	-	50	148	Sterol desaturase	558,093	558,093		1		55.7%
**G3**	0		55	Insertion	100.0%	---	107	321	Tellurite resistance protein TerB	558,406	558,406	R -> RL	3	CGG -> CGG,TTG	56.4%
**G3**	1	(C)5 -> (C)4	42	Deletion (tandem repeat)	100.0%	C				1,193,210	1,193,210				52.4%
**G3**	1	(A)4 -> (A)3	39	Deletion (tandem repeat)	100.0%	A				1,193,328	1,193,328				51.3%
**G4**	2	CC -> TT	72	Substitution	100.0%	CC	303	909	ATP-dependent helicase HrpB	75,286	75,287	EA -> ET	3	GAG,GCC -> GAA,AC	56.9%
**G4**	0		75	Insertion	100.0%	---	107	321	Tellurite resistance protein TerB	558,406	558,406	R -> RL	3	CGG -> CGG,TTG	55.3% -> 56.0%
**G4**	1	(C)5 -> (C)4	37	Deletion (tandem repeat)	100.0%	C				1,193,210	1,193,210				56.8%
**G4**	1	(A)4 -> (A)3	45	Deletion (tandem repeat)	100.0%	A				1,193,328	1,193,328				51.1%
**G4**	1	-C	72	Deletion	100.0%	C	322	965	Putative GTPases (G3E family)	2,925,703	2,925,703		2		51.4%
**G4**	1	(C)3 -> (C)2	90	Deletion (tandem repeat)	100.0%	C	39	115	phage major capsid protein, HK97 family	2,957,827	2,957,827		1		53.3%
**G4**	2	CC -> TT	59	Substitution	98.3% -> 100.0%	CC	379	1,137	hypothetical protein	1,278,931	1,278,932	GA -> GT	3	GGG,GCT -> GGA,AC	50.8%
**G4**	2	(CC)6 -> (CC)5	36	Deletion (tandem repeat)	97.2% -> 100.0%	CC				2,797,449	2,797,450				57.1% -> 58.3%
**G5**	0		78	Insertion	100.0%	---	107	321	Tellurite resistance protein TerB	558,406	558,406	R -> RL	3	CGG -> CGG,TTG	50.0%
**G5**	0	(T)2 -> (T)3	64	Insertion (tandem repeat)	100.0%	-				826,673	826,673				53.1%
**G5**	1	(C)5 -> (C)4	44	Deletion (tandem repeat)	100.0%	C				1,193,210	1,193,210				61.4%
**G5**	1	(A)4 -> (A)3	41	Deletion (tandem repeat)	100.0%	A				1,193,328	1,193,328				58.5%
**G5**	2	CC -> AG	8	Substitution	100.0%	CC				2,517,833	2,517,834				62.5% -> 66.7%
**G5**	2	TT -> GC	23	Substitution	100.0%	TT				2,518,259	2,518,260				56.5%
**G5**	0	(G)3 -> (G)4	12	Insertion (tandem repeat)	100.0%	-				2,518,305	2,518,305				66.7%
**G5**	2	(CC)6 -> (CC)5	36	Deletion (tandem repeat)	97.2% -> 100.0%	CC				2,797,449	2,797,450				68.6% -> 69.4%
**G6**	0		83	Insertion	100.0%	---	107	321	Tellurite resistance protein TerB	558,406	558,406	R -> RL	3	CGG -> CGG,TTG	53.7% -> 54.2%
**G6**	0	(G)2 -> (G)3	98	Insertion (tandem repeat)	100.0%	-	218	654	acetoacetyl-CoA synthase	758,279	758,279		3		51.0%
**G6**	1	(C)5 -> (C)4	56	Deletion (tandem repeat)	100.0%	C				1,193,210	1,193,210				60.7%
**G6**	1	(A)4 -> (A)3	58	Deletion (tandem repeat)	100.0%	A				1,193,328	1,193,328				60.3%
**G6**	1	(C)12 -> (C)11	24	Deletion (tandem repeat)	100.0%	C				2,797,450	2,797,450				83.3%
**G7**	0		69	Insertion	100.0%	---	107	321	Tellurite resistance protein TerB	558,406	558,406	R -> RL	3	CGG -> CGG,TTG	50.7%
**G7**	1	(C)5 -> (C)4	30	Deletion (tandem repeat)	100.0%	C				1,193,210	1,193,210				56.7%
**G7**	1	(A)4 -> (A)3	37	Deletion (tandem repeat)	100.0%	A				1,193,328	1,193,328				56.8%
**G7**	2	(CC)6 -> (CC)5	22	Deletion (tandem repeat)	100.0%	CC				2,797,449	2,797,450				59.1%
**G7**	2	CG -> GT	49	Substitution	100.0%	CG	389	1,166	Adenylate cyclase, family 3 (some proteins containing HAMP domain)	3,132,813	3,132,814	P -> R	2	CCG -> CGT	59.2%
**G8**	1	(G)7 -> (G)6	66	Deletion (tandem repeat)	100.0%	G				888,583	888,583				54.5%
**G8**	1	(C)5 -> (C)4	44	Deletion (tandem repeat)	100.0%	C				1,193,210	1,193,210				56.8%
**G8**	1	(A)4 -> (A)3	40	Deletion (tandem repeat)	100.0%	A				1,193,328	1,193,328				52.5%
**G8**	1	(G)5 -> (G)4	65	Deletion (tandem repeat)	100.0%	G				1,417,135	1,417,135				52.3%
**G8**	0		77	Insertion	100.0%	-				1,938,602	1,938,602				58.4%
**G8**	1	-C	57	Deletion	100.0%	C	90	270	putative transcriptional regulator	2,129,557	2,129,557		3		52.6%
**G8**	2	AT -> TA	71	Substitution	100.0%	AT	1	1	hypothetical protein	2,162,416	2,162,417		1		59.2%
**G8**	0		85	Insertion	98.9% -> 100.0%	---	107	321	Tellurite resistance protein TerB	558,406	558,406	R -> RL	3	CGG -> CGG,TTG	51.7% -> 52.9%
**G8**	2	(CC)6 -> (CC)5	34	Deletion (tandem repeat)	97.1% -> 100.0%	CC				2,797,449	2,797,450				57.6% -> 58.8%
**G9**	1	(C)5 -> (C)4	41	Deletion (tandem repeat)	100.0%	C				1,193,210	1,193,210				58.5%
**G9**	1	(A)4 -> (A)3	41	Deletion (tandem repeat)	100.0%	A				1,193,328	1,193,328				53.7%
**G9**	2	(CC)6 -> (CC)5	36	Deletion (tandem repeat)	100.0%	CC				2,797,449	2,797,450				50.0%
**G9**	0	(G)3 -> (G)4	87	Insertion (tandem repeat)	98.9%	-				2,264,348	2,264,348				50.0%
**G9**	0		64	Insertion	98.4% -> 100.0%	---	107	321	Tellurite resistance protein TerB	558,406	558,406	R -> RL	3	CGG -> CGG,TTG	57.1% -> 57.8%
**G10**	0		47	Insertion	100.0%	---	107	321	Tellurite resistance protein TerB	558,406	558,406	R -> RL	3	CGG -> CGG,TTG	51.0% -> 55.3%
**G10**	1	(C)5 -> (C)4	46	Deletion (tandem repeat)	100.0%	C				1,193,210	1,193,210				52.2%
**G10**	1	(A)4 -> (A)3	44	Deletion (tandem repeat)	100.0%	A				1,193,328	1,193,328				61.4%
**G10**	1	(C)3 -> (C)2	51	Deletion (tandem repeat)	100.0%	C	39	115	phage major capsid protein, HK97 family	2,957,827	2,957,827		1		58.8%
**G10**	2	(CC)6 -> (CC)5	33	Deletion (tandem repeat)	93.9% -> 100.0%	CC				2,797,449	2,797,450				60.6% -> 64.5%
**G11**	0		63	Insertion	100.0%	---	107	321	Tellurite resistance protein TerB	558,406	558,406	R -> RL	3	CGG -> CGG,TTG	58.7% -> 59.7%
**G11**	1	(C)5 -> (C)4	45	Deletion (tandem repeat)	100.0%	C				1,193,210	1,193,210				51.1%
**G11**	1	(A)4 -> (A)3	44	Deletion (tandem repeat)	100.0%	A				1,193,328	1,193,328				54.5%
**G11**	0	(C)2 -> (C)3	57	Insertion (tandem repeat)	100.0%	-	48	144	nitrile hydratase, beta subunit	2,244,656	2,244,656		3		56.1%
**G11**	2	(CC)6 -> (CC)5	25	Deletion (tandem repeat)	96.0% -> 100.0%	CC				2,797,449	2,797,450				60.0% -> 62.5%
**G12**	0		63	Insertion	100.0%	---	107	321	Tellurite resistance protein TerB	558,406	558,406	R -> RL	3	CGG -> CGG,TTG	56.5% -> 57.1%
**G12**	1	(C)6 -> (C)5	50	Deletion (tandem repeat)	100.0%	C				1,044,598	1,044,598				58.0%
**G12**	1	(C)5 -> (C)4	34	Deletion (tandem repeat)	100.0%	C				1,193,210	1,193,210				52.9%
**G12**	1	(A)4 -> (A)3	41	Deletion (tandem repeat)	100.0%	A				1,193,328	1,193,328				61.0%
**G12**	2	(CC)6 -> (CC)5	40	Deletion (tandem repeat)	100.0%	CC				2,797,449	2,797,450				52.5%
**G13**	0		78	Insertion	100.0%	---	107	321	Tellurite resistance protein TerB	558,406	558,406	R -> RL	3	CGG -> CGG,TTG	51.3% -> 52.6%
**G13**	1	(C)5 -> (C)4	51	Deletion (tandem repeat)	100.0%	C				1,193,210	1,193,210				60.8%
**G13**	1	(A)4 -> (A)3	45	Deletion (tandem repeat)	100.0%	A				1,193,328	1,193,328				53.3%
**G13**	0	(G)4 -> (G)5	48	Insertion (tandem repeat)	100.0%	-				2,297,572	2,297,572				52.1%
**G13**	1	(A)3 -> (A)2	52	Deletion (tandem repeat)	100.0%	A	258	772	Protein involved in cell division	2,600,980	2,600,980		1		61.5%
**G13**	2	(CC)6 -> (CC)5	45	Deletion (tandem repeat)	100.0%	CC				2,797,449	2,797,450				64.4%
**G13**	1	-C	52	Deletion	98.1%	C				2,896,122	2,896,122				51.0%
**G13**	2	TT -> AG	51	Substitution	98.1%	TT				2,896,125	2,896,126				52.0% -> 52.9%
