# Supplementary material for: The early events underlying genome evolution in a localized Sinorhizobium meliloti population
Source: BMC Genomics. 2016 Aug 5;17:556. doi: 10.1186/s12864-016-2878-9 (PMC4974801; doi:10.1186/s12864-016-2878-9)
Supplement: Additional file 13: Table S10. — Small indels in the pSymB of the GR4-type isolates. (PDF 58 kb) [file 12864_2016_2878_MOESM13_ESM.pdf]

S10 Table. pSymB indels

isolates	Length	Change	Coverage	Polymorphism Type	Variant Frequency	Sequence	CDS Codon Number	CDS Position	product	Min (original sequence)	Max (original sequence)	Amino Acid Change	CDS Position Within Codon	Codon Change	Strand-Bias
**G1**	0		49	Insertion	100.0%	---				28,796	28,796				53.1% -> 54.2%
**G1**	2	GG -> AA	23	Substitution	100.0%	GG	116	348	D-tagatose-bisphosphate aldolase, class II, non-catalytic subunit	698,084	698,085	TA -> TT	3	ACG,GCC -> ACA,ACC	60.9%
**G1**	2	GA -> TC	44	Substitution	100.0%	GA	629	1,885	Glycosyltransferase	748,33	748,331	S -> E	1	TCG -> GAG	54.5%
**G1**	3	TTA -> ACG	6	Substitution	100.0%	TTA	299	896	GSCFA family	1,141,148	1,141,150	IT -> TS	2	ATA,ACG -> ACG,TCG	57.1% -> 66.7%
**G2**	0		62	Insertion	100.0%	---				28,796	28,796				50.0%
**G2**	2	GA -> TC	45	Substitution	100.0%	GA	629	1,885	Glycosyltransferase	748,33	748,331	S -> E	1	TCG -> GAG	55.6%
**G3**	0		46	Insertion	100.0%	---				28,796	28,796				56.5%
**G3**	2	GA -> TC	43	Substitution	100.0%	GA	629	1,885	Glycosyltransferase	748,33	748,331	S -> E	1	TCG -> GAG	53.5%
**G4**	0		55	Insertion	100.0%	---				28,796	28,796				50.9% -> 51.9%
**G4**	2	GA -> TC	75	Substitution	100.0%	GA	629	1,885	Glycosyltransferase	748,33	748,331	S -> E	1	TCG -> GAG	54.5% -> 54.7%
**G4**	0		87	Insertion	100.0%	-	14	41	ABC-type sugar transport system, periplasmic component	1,235,697	1,235,697		2		50.6%
**G5**	0		66	Insertion	100.0%	---				28,796	28,796				51.5% -> 52.2%
**G5**	2	GG -> AA	49	Substitution	100.0%	GG	35	104	hypothetical protein	1,107,700	1,107,701	A -> V	2	GCC -> GTT	51.0%
**G5**	2	GA -> TC	66	Substitution	98.5% -> 100.0%	GA	629	1,885	Glycosyltransferase	748,33	748,331	S -> E	1	TCG -> GAG	56.1%
**G6**	0		59	Insertion	100.0%	---				28,796	28,796				56.7% -> 57.6%
**G6**	2	GA -> TC	67	Substitution	98.5% -> 100.0%	GA	629	1,885	Glycosyltransferase	748,33	748,331	S -> E	1	TCG -> GAG	50.7% -> 51.5%
**G7**	0		39	Insertion	100.0%	---				28,796	28,796				61.5%
**G7**	2	GA -> TC	41	Substitution	100.0%	GA	629	1,885	Glycosyltransferase	748,33	748,331	S -> E	1	TCG -> GAG	51.2% -> 52.4%
**G8**	0		76	Insertion	100.0%	---				28,796	28,796				53.9% -> 54.7%
**G8**	2	GA -> TC	65	Substitution	100.0%	GA	629	1,885	Glycosyltransferase	748,33	748,331	S -> E	1	TCG -> GAG	50.8% -> 51.5%
**G8**	1	(C)4 -> (C)3	57	Deletion (tandem repeat)	100.0%	C	349	1,047	Xanthine/uracil permease	1,450,991	1,450,991		3		57.9%
**G8**	1		71	Deletion	100.0%	T	24	71	hypothetical protein	1,676,831	1,676,831		2		53.5%
**G9**	0		53	Insertion	100.0%	---				28,796	28,796				56.6%
**G9**	2	GA -> TC	30	Substitution	100.0%	GA	629	1,885	Glycosyltransferase	748,33	748,331	S -> E	1	TCG -> GAG	60.0%
**G9**	1	(T)3 -> (T)2	62	Deletion (tandem repeat)	100.0%	T	176	526	Dehydrogenases with different specificities (related to short-chain alcohol dehydrogenases)	1,528,006	1,528,006		1		62.9%
**G10**	0		56	Insertion	100.0%	---				28,796	28,796				50.0% -> 50.9%
**G10**	2	GA -> TC	28	Substitution	100.0%	GA	629	1,885	Glycosyltransferase	748,33	748,331	S -> E	1	TCG -> GAG	60.7%
**G11**	1		69	Deletion	100.0%	G	158	473	putative protein-tyrosine phosphatase	1,584,865	1,584,865		2		55.1%
**G11**	0		64	Insertion	98.4% -> 100.0%	---				28,796	28,796				50.0% -> 50.8%
**G11**	2	GA -> TC	41	Substitution	97.6% -> 100.0%	GA	629	1,885	Glycosyltransferase	748,33	748,331	S -> E	1	TCG -> GAG	50.0% -> 51.2%
**G12**	0		46	Insertion	100.0%	---				28,796	28,796				58.7%
**G12**	2	GA -> TC	58	Substitution	100.0%	GA	629	1,885	Glycosyltransferase	748,33	748,331	S -> E	1	TCG -> GAG	50.9% -> 51.7%
**G13**	0		43	Insertion	100.0%	---				28,796	28,796				55.8%
**G13**	2	GA -> TC	44	Substitution	100.0%	GA	629	1,885	Glycosyltransferase	748,33	748,331	S -> E	1	TCG -> GAG	50.0% -> 51.1%
