# Supplementary material for: The early events underlying genome evolution in a localized Sinorhizobium meliloti population
Source: BMC Genomics. 2016 Aug 5;17:556. doi: 10.1186/s12864-016-2878-9 (PMC4974801; doi:10.1186/s12864-016-2878-9)
Supplement: Additional file 14: Table S11. — Small indels in the pSymA of the GR4-type isolates. (PDF 58 kb) [file 12864_2016_2878_MOESM14_ESM.pdf]

S11 Table. pSymA indels

Isolate	Length	Change	Coverage	Polymorphism Type	Variant Frequency	Sequence	CDS Codon Number	CDS Position	product	Min (original sequence)	Max (original sequence)	Amino Acid Change	CDS Position Within Codon	Codon Change	Strand-Bias
**G1**	0	(T)4 -> (T)5	89	Insertion (tandem repeat)	98.9%	-	24	71	hypothetical protein	4,355	4,355		2		58.0%
**G3**	1	-C	46	Deletion	100.0%	C				211,185	211,185				52.2%
**G3**	1		47	Deletion	100.0%	A	137	410	ABC-type transport system, involved in lipoprotein release, permease component	872,959	872,959		2		51.1%
**G3**	1	(C)12 -> (C)11	10	Deletion (tandem repeat)	100.0%	C				1,336,948	1,336,948				70.0%
**G4**	1	(C)3 -> (C)2	80	Deletion (tandem repeat)	100.0%	C	244	732	Protein of unknown function (DUF1173)	961,831	961,831		3		55.0%
**G4**	2	TT -> CC	90	Substitution	100.0%	TT	11	31	Small-conductance mechanosensitive channel	1,154,911	1,154,912	L -> P	1	TTG -> CCG	53.3%
**G7**	2	(CT)24 -> (CT	16	Deletion (tandem repeat)	100.0%	CT				162,044	162,045				56.3%
**G8**	3		76	Deletion	100.0%	ATC	37	109	protein-L-isoaspartate(D-aspartate) O-methyltransferase	543,426	543,428	D ->	1	GAT ->	51.3%
**G8**	1	-C	72	Deletion	100.0%	C				772,429	772,429				54.2%
**G8**	3	CTG -> TGC	58	Substitution	100.0%	CTG				1,118,240	1,118,238				56.9% -> 58.3%
**G8**	0		65	Insertion	100.0%	-				1,118,194	1,118,194				53.8%
**G8**	1	-C	67	Deletion	100.0%	C	53	159	ABC-type sugar transport system, periplasmic component	1,262,969	1,262,969		3		55.2%
**G8**	2	AT -> CA	61	Substitution	98.4% -> 100.0%	AT				1,118,227	1,118,226				57.4%
**G9**	3	CGC -> GAT	7	Substitution	100.0%	CGC				502,092	502,094				75.0% -> 85.7%
**G10**	2	TT -> CC	40	Substitution	100.0%	TT	11	31	Small-conductance mechanosensitive channel	1,154,911	1,154,912	L -> P	1	TTG -> CCG	62.5%
**G11**	3	TAA -> ACC	6	Substitution	100.0%	TAA				502,088	502,09				66.7% -> 71.4%
**G11**	2	TG -> AC	39	Substitution	100.0%	TG	89	267	Nitric oxide reductase activation protein	708,683	708,684	HA -> QP	3	CAT,GCG -> CAA,CC	59.0%
**G12**	3		53	Deletion	100.0%	CTC	368	1,104	hypothetical protein	461,372	461,374	LS -> L	3	CTC,TCA -> CTA	52.8%
**G13**	0	(G)2 -> (G)3	64	Insertion (tandem repeat)	98.4%	-	249	747	Bacterial protein of unknown function (DUF894)	265,45	265,45		3		60.3%
