# Supplementary material for: The early events underlying genome evolution in a localized Sinorhizobium meliloti population
Source: BMC Genomics. 2016 Aug 5;17:556. doi: 10.1186/s12864-016-2878-9 (PMC4974801; doi:10.1186/s12864-016-2878-9)
Supplement: Additional file 16: Figure S5. — Alignment of repeat sequences. Strain GR4 pSymA IGR positions 1,116,938 to 1,117,089 (repeat 1) and 1,145,069 to 1,145,218 (repeat 2) and those identified in G4 and G10 isolates. Discordant nucleotide positions are highlighted. (PDF 210 kb) [file 12864_2016_2878_MOESM16_ESM.pdf]

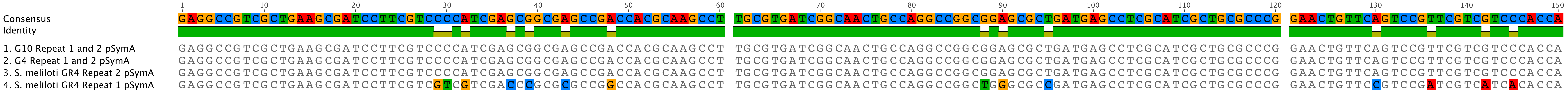
1 10 20 30 40 50 60 70 80 90 100 110 120 130 140 150
Consensus
GAGGCCGTCGCTGAAGCGATCCTTCGTCCCCATCGAGCGGCGAGCCGACCACGCAAGCCT TGCGTGATCGGCAACTGCCAGGCCGGCGGAGCGCTGATGAGCCTCGCATCGCTGCGCCCG GAACTGTTCAGTCCGTTCGTCGTCCCACCA
Identity
1. G10 Repeat 1 and 2 pSymA
GAGGCCGTCGCTGAAGCGATCCTTCGTCCCCATCGAGCGGCGAGCCGACCACGCAAGCCT TGCGTGATCGGCAACTGCCAGGCCGGCGGAGCGCTGATGAGCCTCGCATCGCTGCGCCCG GAACTGTTCAGTCCGTTCGTCGTCCCACCA
2. G4 Repeat 1 and 2 pSymA
GAGGCCGTCGCTGAAGCGATCCTTCGTCCCCATCGAGCGGCGAGCCGACCACGCAAGCCT TGCGTGATCGGCAACTGCCAGGCCGGCGGAGCGCTGATGAGCCTCGCATCGCTGCGCCCG GAACTGTTCAGTCCGTTCGTCGTCCCACCA
3. S. meliloti GR4 Repeat 2 pSymA
GAGGCCGTCGCTGAAGCGATCCTTCGTCCCCATCGAGCGGCGAGCCGACCACGCAAGCCT TGCGTGATCGGCAACTGCCAGGCCGGCGGAGCGCTGATGAGCCTCGCATCGCTGCGCCCG GAACTGTTCAGTCCGTTCGTCGTCCCACCA
4. S. meliloti GR4 Repeat 1 pSymA
GAGGCCGTCGCTGAAGCGATCCTTCGTCGTCGTCGACCCGCGCGCCGGCCACGCAAGCCT TGCGTGATCGGCAACTGCCAGGCCGGCTGGCGCCGATGAGCCTCGCATCGCTGCGCCCG GAACTGTTCCGTCCGATCGTCATCACACCA
