# Supplementary material for: The early events underlying genome evolution in a localized Sinorhizobium meliloti population
Source: BMC Genomics. 2016 Aug 5;17:556. doi: 10.1186/s12864-016-2878-9 (PMC4974801; doi:10.1186/s12864-016-2878-9)
Supplement: Additional file 19: Table S12. — Variants due to mobile elements in the GR4-type isolates. (PDF 77 kb) [file 12864_2016_2878_MOESM19_ESM.pdf]

Table S12. Variations in the GR4-type isolates caused by mobile elements

Mobile element	Strains/isolates	Product	Locus tag	Replicon	Positions
**GII intron RmInt1**	G4, G8 and G12	D-alanine esterification of lipoteichoic acid and wall teichoic acid	GR4pD0623	pSymB	689,290
	G3	3-hydroxyisobutyrate dehydrogenase	GR4Chr1817	Chromosome	1,885,446
**IS*Rm14* (IS66 family)**	GR4			pSymA	502,112-504,805
	GR4	hypothetical protein	GR4pC0935	pSymA	917,762-920,417
	G6	hypothetical protein	GR4pC0935	pSymA	917,762
	G5	IGR		pSymA	1,227,997
	G8	CheY-like receiver domain and a winged-helix DNA-binding domain protein	(GR4pC0939)	pSymA	923,796
	G10	IGR		pSymA	1,040,300
	G12	IGR		pSymA	881,553
**IS*Rm2011-2* (IS*Rm11*)**	G7	protein containing double-stranded beta-helix domain	GR4pC0266	pSymA	217,253
**Transposase-like (GR4pD1125 and GR4pD1126)**	G9	IGR		pSymA	853,534
**Transposase-like (GR4pD1125 and GR4pD1126)**	G13	IGR		pSymB	1,037.004
**Unknown (IS66 family)**	GR4			pSymB	1,138,150-1,140.963
	G6			pSymB	1,138,150
**Unknown (IS256 family)**	G2	ABC-type sugar transport system, periplasmic component	GR4pD1076	pSymB	1,199,503
**TRm19 (IS*Rm19*)**	GR4			Chromosome	2,237,307-2,240,887
	G4, G10			Chromosome	1,247,993
	G6			Chromosome	2,187,959
	G6			Chromosome	2,199.081
	G8			Chromosome	331,342
	G8			Chromosome	1,147,003
**TRm24 (IS*Rm24*)**	G4			Chromosome	1,708,773
**IS*Rm9* (IS21 family)**	G12			Chromosome	2,838,831
**IS*Rm9* (IS21 family)**	G9	IGR		pSymA	1,085,291
**yecA, GR4pB042 and GR4pB043 (IS66 family)**	G9	IGR		pSymA	1,025,679
**yecA, GR4pB042 and GR4pB043 (IS66 family)**	G4, G10	hypothetical protein	GR4pD0997	pSymB	1,118,350
**yecA, GR4pB042 and GR4pB043 (IS66 family)**	G6	IGR		Chromosome	2,485,653
